# Development, validation, and application of a multi-method for the determination of mycotoxins, plant growth regulators, tropane alkaloids, and pesticides in cereals by two-dimensional liquid chromatography tandem mass spectrometry

**DOI:** 10.1007/s00216-021-03239-1

**Published:** 2021-03-13

**Authors:** Ann-Kristin Rausch, Robert Brockmeyer, Tanja Schwerdtle

**Affiliations:** 1 Department of Food Chemistry, Institute of Nutritional Science, University of Potsdam, Arthur-Scheunert-Allee 114-116, 14558 Nuthetal, Germany; 2 Eurofins SOFIA GmbH, Rudower Chaussee 29, 12489 Berlin, Germany; 3 German Federal Institute for Risk Assessment, Max-Dohrn-Straße 8-10, 10589 Berlin, Germany

**Keywords:** 2D-LC-MS/MS, Multi-method, Mycotoxins, Modified mycotoxins, Pesticides, Cereals

## Abstract

Mycotoxins and pesticides regularly co-occur in agricultural products worldwide. Thus, humans can be exposed to both toxic contaminants and pesticides simultaneously, and multi-methods assessing the occurrence of various food contaminants and residues in a single method are necessary. A two-dimensional high performance liquid chromatography tandem mass spectrometry method for the analysis of 40 (modified) mycotoxins, two plant growth regulators, two tropane alkaloids, and 334 pesticides in cereals was developed. After an acetonitrile/water/formic acid (79:20:1, v/v/v) multi-analyte extraction procedure, extracts were injected into the two-dimensional setup, and an online clean-up was performed. The method was validated according to Commission Decision (EC) no. 657/2002 and document N° SANTE/12682/2019. Good linearity (*R*^2^ > 0.96), recovery data between 70-120%, repeatability and reproducibility values < 20%, and expanded measurement uncertainties < 50% were obtained for a wide range of analytes, including very polar substances like deoxynivalenol-3-glucoside and methamidophos. However, results for fumonisins, zearalenone-14,16-disulfate, acid-labile pesticides, and carbamates were unsatisfying. Limits of quantification meeting maximum (residue) limits were achieved for most analytes. Matrix effects varied highly (−85 to +1574%) and were mainly observed for analytes eluting in the first dimension and early-eluting analytes in the second dimension. The application of the method demonstrated the co-occurrence of different types of cereals with 28 toxins and pesticides. Overall, 86% of the samples showed positive findings with at least one mycotoxin, plant growth regulator, or pesticide.

**Supplementary Information:**

The online version contains supplementary material available at 10.1007/s00216-021-03239-1.

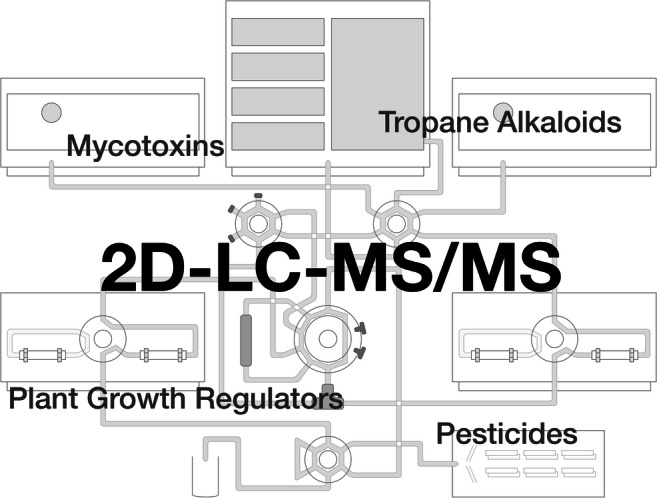

## Introduction

Cereal crops are major staple foods, while wheat accounts for more than half of the cereal production in the European Union (EU) [1]. To obtain a high yield, crops are often treated with pesticides and fertilizers. Due to their plant protection ability, they defend against crop losses, e.g., due to fungi, weeds, or insects [2]. Moreover, crops can be contaminated with toxic compounds. Especially under humid conditions, cereals are often infected with fungi, which can produce mycotoxins as toxic secondary products [3]. Further contamination sources represent plant growth regulators and tropane alkaloids. The latter contaminate cereals by co-harvested undesired weeds [4].

The application of pesticides and fertilizers to prevent crops from being attacked by undesired pests improves food production worldwide. Pesticides ensure practically one-third of the plant production. Nevertheless, heavy use has been criticized for possible pest resistance, health problems, and environmental impact [5, 6]. The European Commission (EC) has set maximum required limits (MRLs) for pesticides in or on food and feed of plant and animal origin in Commission Regulation (EC) no. 396/2005 to protect humans [7].

Mycotoxins are low-molecular-weight contaminants that can cause toxicological effects while their prevalence is almost unavoidable [3, 8]. The establishment of rapid, accurate, and reliable analytical methods has become essential to investigate their presence, conduct risk assessments, and remove them as far as possible from the food chain [9]. Maximum levels (MLs) for mycotoxins in food and feed are regulated in Commission Regulation (EC) no. 1881/2006 [10]. In contrast to free mycotoxins, modified mycotoxins are not yet regulated in the EU. These substances can be biosynthesized by the impact of the plant, animal, and fungi enzymes, formed during food processing, and co-occur with their free forms [9]. Modified mycotoxins are often not detectable in the routine analysis but contribute to toxicity as well [11].

Plant growth regulators chlormequat and mepiquat are quaternary ammonium pesticides approved for cereal cultivation [12]. They reduce the cell elongation, decrease the growth of stalks, and prevent lodging [13, 14]. MRLs are established in Commission Regulation (EC) no. 396/2005 [7].

Tropane alkaloids are natural toxins, occurring in particular in Brassicaceae and Solanaceae. Like mycotoxins, they are produced as secondary products and contaminate cereals during harvest or food and feed processing [4, 15]. Most studied toxins are atropine and scopolamine, for which maximum levels are established in Commission Recommendation (EU) no. 2015/976 [16] and Commission Regulation (EU) no. 2016/239 in certain cereal-based foods for infants and young children [17].

Because of the possible simultaneous contamination of grain with contaminants and residues, the concurrent analysis of these substances is necessary for food safety and food control.

A common method for sample preparation in pesticide analysis is the QuEChERS method, which stands for quick, easy, cheap, effective, rugged, and safe [18]. After an extraction, polar compounds are separated from unpolar analytes. Other clean-up procedures involve solid phase extraction (SPE), matrix solid phase dispersion (MSPD), or liquid-liquid extraction (LLE) [5, 18]. Mycotoxins are often analyzed after clean-up using SPE or immune-affinity columns (IAC) [19]. However, the sample preparation should be as simple and fast as possible when a wide range of analytes is measured in one chromatographic run.

While liquid chromatography (LC) methods are usually the method of choice for non-volatile or thermally labile analytes, gas chromatography (GC) methods are used for volatile, thermally stable compounds. To analyze a large number of substances, like the ones presented before, at the same time, a high performance liquid chromatography tandem mass spectrometry (HPLC-MS/MS) method is required, which offers sufficient peak capacity, high resolving power, and, ideally, carries out an online clean-up. Since one-dimensional liquid chromatography (1D-LC) cannot meet these requirements, the use of two-dimensional liquid chromatography (2D-LC) is a solution. 2D-LC offers selectivity and separation efficiency by coupling two independent columns. The peak capacity is enhanced drastically, resulting in an increased resolving power, which presents the most crucial advantage of 2D-LC setups. A fundamental distinction is made between heart-cutting (LC-LC) and comprehensive (LCxLC) liquid chromatography. In LC-LC, selected fractions are transferred from the first dimension to the second dimension, while in LCxLC, the whole effluent is analyzed through both separation stages [20–23]. Furthermore, 2D-LC approaches can be extended by a solid phase extraction for the storage of specific fractions.

Orthogonality is achieved by two different separation techniques in both dimensions. However, this constitutes the critical point of 2D-LC systems. The mobile phase of the fraction from the first dimension is often not optimal for separating the early eluting analytes on the second dimension. This may cause low retention, peak broadening, and decreased separation efficiency [24].

Kittlaus et al. (2013) developed a 2D-LC system to analyze more than 300 pesticides [25]. This system used a modified LC-LC approach. It is based on the use of hydrophilic interaction liquid chromatography (HILIC) as the first and reversed phase (RP) chromatography as the second dimension. The early-eluting unpolar analytes from the HILIC column are trapped in a kind of packed loop interface in one single fraction and thus separated from the interfering matrix on the HILIC column. The trapping simultaneously facilitates the necessary exchange of the organic solvent from the first dimension, which offers a too high elution strength for the second dimension. Polar analytes from the HILIC column are analyzed by MS/MS directly after trapping of the unpolar ones is finished, and lastly, separation of the trapped compounds is carried out on the RP column. This approach provides an online clean-up step, which results in short and time-saving sample preparation for the analysis of multiple analyte classes [25].

In the past, further 2D-LC methods were introduced not only for the analysis of pesticide residues but also for the simultaneous quantification of contaminants. Urban et al. (2019) analyzed 370 pesticides, two tropane alkaloids, 30 pyrrolizidine alkaloids, two plant growth regulators, and nine mycotoxins by a 2D-LC-MS/MS method [26]. In the same year, Kresse et al. published a 2D-LC-MS/MS method for the simultaneous determination of 350 pesticides, 16 mycotoxins, two tropane alkaloids, and two plant growth regulators [27]. A concept for the analysis of pesticides and polar pesticides by 2D-LC high resolution mass spectrometry (HRMS) was presented by Jost and Habedank (2020) [28]. 2D methods, coupled to GC, are used to analyze pesticides and contaminants concurrently as well [28–31]. Even though 2D-LC methods for the analysis of mycotoxins are published not as often as for pesticides, they are still an emerging trend [29–31].

This study aimed to develop and validate a 2D-LC-MS/MS multi-method. Based on the system designed by Kittlaus et al. (2013), 24 free and 16 modified mycotoxins, two plant growth regulators, two tropane alkaloids, and 334 pesticides were analyzed. The focus was set on the integration of relevant toxic modified mycotoxins. To our knowledge, this is the first 2D-LC-MS/MS method that includes such a high number of pesticides and mycotoxins at the same time. Most modified, as well as free mycotoxins, were integrated into a 2D-LC method for the first time.

## Materials and methods

### Reagents and chemicals

Methanol (MeOH) and acetonitrile (ACN) were purchased from Merck KGaA (Darmstadt, Germany) in HPLC grade. Ammonium formate (AFNH_4_) was obtained from Sigma-Aldrich (Steinheim, Germany). Th. Geyer (Renningen, Germany) supplied formic acid (FA). Purified water was produced using a Milli-Q® apparatus (Integral ultrapure water [type 1], 18.2 MΩ∙cm, Merck KGaA, Darmstadt, Germany).

### Standards

Mycotoxin reference standards were supplied by Sigma-Aldrich (Steinheim, Germany), LGC Standards (Wesel, Germany), HPC Standards (Cunnersdorf, Germany), ASCA (Berlin, Germany), Cfm Oskar Tropitzsch (Marktredwitz, Germany), WITEGA Laboratorien Berlin-Adlershof (Berlin, Germany), Toronto Research Chemicals (Toronto, ON, Canada), and Romer Labs Diagnostic (Tulln, Austria). Hydrolyzed fumonisin standards were prepared as described previously in Rausch et al. (2020) [32]. Plant growth regulators and tropane alkaloids were purchased from LGC Standards (Wesel, Germany) and Honeywell (Seelze, Germany). All pesticide and internal standards (ISTD) were obtained from Sigma-Aldrich (Steinheim, Germany), LGC Standards (Wesel, Germany), and HPC Standards (Cunnersdorf, Germany). Analyte-dependent stock solutions were prepared at different concentration levels. A combined multi-standard working solution, containing all analytes (except deoxynivalenol-3-glucoside), was prepared in ACN (acidified with FA, 0.05%) and stored at 5 °C (see Supplementary Information (ESM) Table S1). Deoxynivalenol-3-glucoside was prepared as an additional working solution with a concentration of 50 μg/mL since the reference solution was only available at this concentration. Deuterated internal standards (ISTD) zearalenone dimethyl ether-d6, chlormequat-d4, mepiquat-d4, carbendazim-d4, imidacloprid-d4, diazinon-d10, and diuron-d6 were combined in an ISTD working solution (see ESM Table S2). Propamocarb-d7 was used as an injection control standard.

### Sample preparation

Using an ultra-centrifugal mill ZM 200 (Retsch, Haan, Germany), samples were ground by cryogenic milling. A total of 2.50 ± 0.02 g of the sample was weighed into a 50 mL disposable screw-capped polypropylene centrifuge tube (VWR, Darmstadt, Germany). A volume of 10 mL of an extraction mixture of ACN/water/FA (79:20:1, v/v/v) was added. The tube was shaken vigorously, and the matrix was allowed to soak for 15 min at room temperature. Afterward, the sample was spiked with 20 μL of the ISTD solution and mixed using the Reax top vortex mixer (VWR, Darmstadt, Germany). Extraction was carried out by rotating the sample for 30 min using a Reax 2 overhead shaker (Heidolph Instruments, Schwabach, Germany). After that, the extract was centrifuged for 3 min at 1902×*g* (Centrifuge 5810, Eppendorf, Hamburg, Germany). An aliquot of the supernatant was filtered through Chromafil RC-20/15 filters (MACHEREY-NAGEL, Düren, Germany), followed by dilution with ACN, spiked with propamocarb-d7 (10 μg/L), to the same extent. The final extract was transferred into a plastic vial (VWR, Darmstadt, Germany).

### 2D-LC-MS/MS analysis

**Fig. 1:**
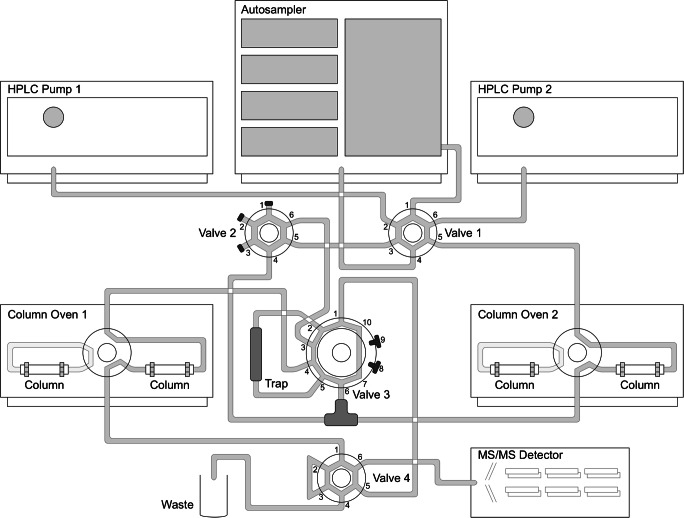
2D-LC-MS/MS system

The 2D-LC-MS/MS analysis was performed using a 1260 Infinity II HPLC system equipped with two pumps, two degassers, and two column ovens (Agilent, Waldbronn, Germany). For the coupling of both pumps and column ovens, three 6-port and one 10-port valves were used. The HPLC was coupled to a 5500 triple quadrupole mass spectrometer provided with an electrospray ionization (ESI) interface (Sciex, Darmstadt, Germany). A detailed setup of the 2D-LC-MS/MS system is shown in Fig. 1. Chromatographic separation was performed using different columns, mobile phases, and gradients for both dimensions. In the first dimension, analytes were separated on a HILIC column, namely a YMC-Pack Diol-NP 5 μm, 120 Å, 100 × 2.1 mm (Dinslaken, Germany) equipped with a 5 μm, 120 Å, 10 × 2.1 mm guard column cartridge. Mobile phase A (water, 0.1% FA, 10 mM ammonium formate) and mobile phase B (ACN/water [90:10, v/v], 0.1% FA, 10 mM ammonium formate) were optimized. The gradient elution applied is summarized in Table 1. A trap column consisting of three columns connected in series was installed for coupling the first and second dimension by storing the analytes for the second dimension in the packed loop interface during the first dimension analysis. The three columns used were ZORBAX SB-C8 5 μm, 80 Å, 12 × 4.6 mm (Agilent, Waldbronn, Germany). Second dimension separation was performed on two columns connected in series, a Raptor FluoroPhenyl 2.7 μm, 50 × 2.1 mm and Raptor Biphenyl 2.7 μm, 50 × 2.1 mm, equipped with a FluoroPhenyl 2.7 μm, 5 × 2.1 mm guard column cartridge (Restek, Bad Homburg, Germany). Mobile phase A (water, 0.001% FA, 5 mm ammonium formate) and mobile phase B (MeOH, 0.001% FA, 5 mM ammonium formate) were applied. The gradient used for the second dimension is displayed in Table 1 as well. Furthermore, valve phases and switching times are listed. Flow rates varied during both dimensions. While in the first dimension, a constant flow rate of 200 μL/min (pump 2) was chosen, in the second dimension, a flow rate of 300 μL/min (pump 1) was applied in the first phase of equilibration. In the next phase, collecting the early eluting analytes from the HILIC column on the trap column, the flow rate of pump 1 was increased to 2000 μL/min. A total of 100% of mobile phase A were used and mixed with the eluate of pump 2 using a mixing t-piece. During the third phase, analysis of (first dimension) HILIC analytes, the flow rate of pump 1 was set to 0 μL/min, and in the last phase, the initial flow rate of 300 μL/min for pump 1 was re-established for analysis of the RP analytes. The injection volume was 10 μL. Column temperatures of both column ovens were set to 40 °C. The total run time was 25 min.

**Table 1:** Gradient elution system for 2D-LC-MS/MS analysis

Phase	Pump 2 (HILIC)^a^, 40 °C, 500 bar			Pump 1 (RP)^b^, 40 °C, 590 bar		
	Time [min]	*B* [%]	Flow rate [μL/min]	Time [min]	*B* [%]	Flow rate [μL/min]
1	0.00	100	200	0.00	5	300
2				1.20	5	300
				1.30	0	2000
3				2.15	0	2000
				2.20	0	0
	2.50	100	200			
	3.00	90	200	3.00	0	0
	3.80	20	200			
				7.45	0	0
4				7.50	5	300
	7.60	20	200			
	7.80	100	200			
				8.20	50	300
				13.00	70	300
				15.50	85	300
				18.50	100	300
				20.50	100	300
				21.00	5	300
	25.00	100	200	25.00	5	300

^a^Mobile phase A: water, 0.1% FA, 10 mm ammonium formate; mobile phase B: ACN/water (90:10, v/v), 0.1% FA, 10 mM ammonium formate

^b^Mobile phase A: water, 0.001% FA, 5 mm ammonium formate; mobile phase B: MeOH, 0.001% FA, 5 mM ammonium formate

The mass spectrometer was performed in the scheduled multiple reaction mode (sMRM) in positive and negative ESI modes, scanning the two most intensive transitions for each analyte. The settings of the ion source were set as follows: source temperature (500 °C), curtain gas (40 psi), ion source gas 1 (60 psi), ion source gas 2 (65 psi), ion spray voltage in positive/negative ionization mode (4500 V/−4500 V), spray and collision cell gas (nitrogen). Optimized analyte-dependent MS/MS parameters, including declustering potentials (DPs), collision energies (CEs), collision exit potentials (CXPs), and retention times (RTs), are listed in ESM (Table S3).

### Method validation

The multi-method was validated according to the criteria set in Commission Decision (EC) no. 657/2002 [33] and Regulation (EC) no. 401/2006 [34] for mycotoxins, and guidelines defined in document N° SANTE/12682/2019 [35]. The calculated validation parameters were the following: linearity, the limit of quantification (LOQ), recovery, precision, and measurement uncertainty. Furthermore, matrix effects (MEs) were assessed and compensated for using standard addition for routine sample analysis. Wheat was selected as a representative matrix for cereal validation material since it is known as being vulnerable to contamination with mycotoxins, plant growth regulators, and tropane alkaloids, as well as pesticides. Blank samples were spiked with the multi-standard working solution and deoxynivalenol-3-glucoside at different concentration levels (see ESM Table S4). Validation experiments were carried out on three different days.

The linearity was assessed by determination of the calibration curve equations and coefficients of determination (*R*^2^). The LOQ was set as the lowest spiking point and calculated based on the signal-to-noise (S/N) ratios of at least 10, according to the International Union of Pure and Applied Chemistry (IUPAC). For all regulated substances, LOQs should meet the MLs set in Commission Regulation (EC) no. 1881/2006 for contaminants [10] and Commission Regulation (EC) no. 396/2005 for pesticides [7]. Absolute recoveries (*R*_E_, *n* = 6) were expressed as the mean recovery determined by ratios of matrix-spiked to matrix-matched standards at different concentration levels. Repeatability (RSDr) and within-laboratory reproducibility (RSDR) were obtained by calculation of the relative standard deviations (RSDs) of a set of six replicates three-times on the same day or at different days, respectively. The expanded measurement uncertainty (MU, *k* = 2) was calculated based on intra-laboratory validation data and a confidence level of 95%. MEs were assessed by the ratios of matrix-matched to solvent-only standards. Matrix suppression was visualized in negative values, while matrix enhancement was presented in positive values.

### Analysis of cereal samples

By using the standard addition approach, a set of 36 samples were screened for all analytes. More precisely, wheat, barley, rice, oat, spelt, and rye samples were investigated for the co-occurrence of contaminants and pesticides. Samples were spiked with known concentrations of the multi-standard working solution and deoxynivalenol-3-glucoside at different concentrations. An ISTD solution mix was added to all samples at the beginning of the sample preparation. The signal intensities of the ISTDs must be in the range of 0.8 – 1.2 between samples and spiked samples. The results were adjusted by this factor.

### Data analysis

Analyst software version 1.6.3 and MultiQuant software version 3.0.3 (Sciex, Darmstadt, Germany) were applied for data acquisition and evaluation. Furthermore, Microsoft Excel 2010 (Microsoft Co., Redmond, WA, USA) and custom software developed in Node.JS (Linux Foundation, San Francisco, CA, USA) were used for data processing.

## Results and discussion

### Method development

#### Development of chromatographic separation by 2D-LC-MS/MS

Using both positive and negative ionization modes, analytes were optimized, and the two most intensive transitions were determined. As shown in ESM Table S3, most analytes were analyzed in positive ESI mode. Since some mycotoxins revealed a significantly higher ionization response in the negative mode, polarity switching took place.

Next, a 2D-LC-MS/MS system was installed since the study aimed to analyze pesticides and mycotoxins in one chromatographic run without classical clean-up steps in sample preparation. Furthermore, a 1D-LC method would not have provided a sufficient resolution for all analytes resulting in co-elution and unresolved peaks. For this approach, a complex setup, as displayed in Fig. 1, was assembled. This procedure allowed the separation of matrix compounds as well as the maximization of the peak capacity and resolving power for a huge amount of analytes. The two-dimensional method consists of four different phases. In the first phase, samples are injected and the columns are equilibrated. In the next phase, unpolar analytes elute from the HILIC column and are collected on a trap column. Thus, a high flow of mobile phase A consisting of water is used because the fraction contains a high amount of organic solvent, which has to be changed before the RP analysis starts. In the third phase, the remaining very polar substances on the HILIC column are analyzed by eluting directly into the MS/MS. The last phase starts immediately after the elution of the HILIC analytes. The unpolar substances stored on the trap interface are separated on an RP-column combination. During this phase, remaining matrix compounds on the HILIC column are directed into the waste. This allows the replacement of complex sample preparation by separation of the interfering matrix in an online clean-up.

The two-dimensional LC-MS/MS method offers many parts that can be developed and optimized. In Table 2, all evaluated columns for the first and second dimensions, as well as trap columns, are listed. In the first step of method development, we tested different kinds of polar stationary phases for the first dimension (mainly HILIC columns). The aim of using a HILIC column was to purify the extracts by a highly polar mobile phase, which results in the compensation of classical liquid-liquid extraction steps in sample preparation [25]. The columns differed significantly in their retention ability. Most columns revealed late eluting peaks with a broad peak shape. Since the aim was to keep the measurement of the first dimension as short as possible, columns with retention of more than 8 min were excluded.

**Table 2:** Tested columns during 2D-LC-MS/MS method development

2D-LC setup	Name	Dimension
1. Dimension	YMC-Pack Diol-NP	100 × 2.1 mm; 5 μm; 120 Å
	Raptor HILIC-Si	100 × 2.1 mm; 2.7 μm; 90 Å
	Cortecs UPLC HILIC	100 × 2.1 mm; 1.6 μm; 90 Å
	Obelisc R	100 × 2.1 mm; 5 μm; 120 Å
	SeQuant ZIC-HILIC	150 × 2.1 mm; 5 μm; 200 Å
	Hypercarb	100 × 2.1 mm; 5 μm; 250 Å
	Acquity UPLC BEH HILIC	50 × 2.1 mm; 1.7 μm; 130 Å
	Luna Omega Sugar	100 × 2.1 mm; 3 μm; 100 Å
	Torus DEA	100 × 2.1 mm; 1.7 μm; 130 Å
	Raptor Polar X	100 × 2.1 mm; 2.7 μm; 130 Å
Trap	ZORBAX SB-C8	3 times 12.5 × 4.6 mm; 5 μm
	ZORBAX SB-C8	12.5 × 4.6 mm; 5 μm
	ZORBAX SB-C8	50 × 4.6 mm; 5 μm
	ZORBAX Extend-C18	12.5 × 4.6 mm; 5 μm
	ZORBAX Eclipse XDB-Phenyl	12.5 × 4.6 mm; 5 μm
	ZORBAX SB-AQ	12.5 × 4.6 mm; 5 μm
2. Dimension	Raptor FluoroPhenyl + Raptor Biphenyl	Each 50 × 2.1 mm; 2.7 μm; 100 Å
	Synergi Fusion-RP	100 × 2.0 mm; 2.5 μm; 90 Å
	Raptor FluoroPhenyl	100 × 2.1 mm; 2.7 μm; 100 Å
	Raptor Biphenyl	100 × 2.1 mm; 2.7 μm; 100 Å

Furthermore, fumonisins were neither trapped nor analyzed on most columns due to strong retention. The only column that was able to analyze all polar substances within 7.5 min was the YMC Pack Diol-NP. As this column does not allow for the chromatographic separation of fumonisins B2 and B3, an additional confirmation method is necessary when fumonisin B2/B3 is detected using the advanced screening method. A total of 359 (95%) of the analytes showed low retention and eluted at the beginning of the chromatographic HILIC run, while 19 analytes (5%) were measured on this column. Next, the gradient system was revised, followed by mobile phase development. Ammonium salt and acid concentrations were optimized. Acidic conditions showed better results for carboxylic acids like daminozide, while unacidified eluents revealed higher intensities of fumonisins. Due to the high amount of analytes of the multi-method and thus many different chemical properties, the developed method has always to be a compromise. Therefore, an ammonium formate concentration of 10 mM and acid content of 0.1% FA were chosen for both mobile phases [A: water; B: ACN/water (90:10, v/v)]. In the next step, different trapping times and materials were tested. Different settings, more precisely trapping until minutes 1.95, 2.0, 2.05, 2.1, and 2.15, were used to optimize the switching point between phase 2 and phase 3. After 1.95, 2.0, and 2.05 min, changing the valve positions led to split peaks of polar substances like, e.g., methamidophos or glucosylated modified mycotoxins. Trapping until minutes 2.1 and 2.15 revealed less peak splitting. However, with increasing trapping time, aminocarb no longer showed retention on the trap column. Overall, the best results were obtained by switching the valves after 2.15 min. These tests were carried out with a ZORBAX SB-C8 trap column. In the next step, other trapping materials were tested, and the retention capacity was compared. The SB-C8 column showed the highest retention ability and best results compared to other traps. However, since the retention of a few analytes could be even better, we compared using one SB-C8 trap column with three SB-C8 trap columns connected in series and a 50 mm trap column of the same type. The connection of the three columns led to a better enrichment of the analytes. For example, aldoxycarb, methomyl, and omethoate were not saved as well using only one trap column, but they were stored optimally by using three trap columns. Compared to the 50 mm trap column, the same retention capacity was obtained for all analytes except for the mycotoxin deoxynivalenol, which showed more unsatisfactory results. Since deoxynivalenol is ubiquitous in nature and one of the most contaminating mycotoxins, the use of the 50 mm column was excluded, even if it constitutes a more stable system. Because the fumonisins showed very strong retention on the HILIC column used, an approach was briefly followed to retain them after the HILIC analysis (phase 3) and thus to incorporate two trap phases into the method. However, due to excessive flow on the trap column, some saved analytes were already flowing into the waste, and the approach was not further optimized. Lastly, the analysis of the analytes trapped on the second dimension was optimized. For this, different columns, gradients, and mobile phases were tested. The focus was set on the separation of isomers, peak shapes, and peak areas, which revealed the best results using the connection of two Raptor columns, namely Raptor FluoroPhenyl and Biphenyl. Variations between all tested columns were observed, especially for the peak shapes. Increased peak broadening was observed for polar toxins eluting first due to the slightly incompatible injection solvent. The gradient and mobile phase composition was finally optimized. Different combinations of water and organic solvents with different acid and ammonium salt concentrations were applied. Mobile phases consisting of water and MeOH, both added with 5 mM ammonium formate and 0.001% FA, were finally used.

The main advantage of using the developed method, a 2D-LC approach combined with solid phase extraction, posed the simultaneous analysis of different chemical groups with varying properties. Especially the high polarity range of the investigated analytes and enormous peak capacity was covered by the developed 2D-LC setup. Besides, the applied online clean-up led to a reduced sample preparation, which is essential for routine analysis. Multi-methods based on a QuEChERS extraction approach [36–39] or the analytical procedure proposed by Klein and Alder (2003) [40] are comparatively time-consuming but have their advantages in sample purification and thus resulting matrix effects. Furthermore, the reduction in analysis time is noteworthy. Even if there are already LC-MS/MS methods published that record a combination of distinct chemical classes in one measurement [36, 39, 41], these still represent the minority. Such other multi-class methods, covering the analysis of pesticides, mycotoxins, and partially plant growth regulators and antibiotics, rest on 1D-LC-MS/MS methods, which offer high sensitivity and selectivity [39, 41]. Nevertheless, various analyte groups such as pesticides and mycotoxins are measured often in individual multi-methods, so several analytical runs per sample are necessary. At the same time, disadvantages arise using 2D-LC methods. Besides the high costs and particular care of the instrumentation, limitations in the methods sensitivity, analysis time of the RP dimension, and solvent compatibility affecting the methods validation data may occur. Overall, comparing extremely high-throughput methods like the presented method with focused approaches based on particular groups of compounds reveals that fast results are possible with the first mentioned screening methods. When gaining positive findings, the latter methods are beneficial for confirmation purposes with, for instance, lower LOQs.

#### Optimization of extraction process

The extraction process is a critical step in method development for multi-methods since a wide range of different polarities and chemical compounds are included. The aim of this process was to develop a simple and fast sample preparation without complex purification steps. The extraction tests were carried out using different solvent combinations containing ACN, water, and partly FA. First, the following extraction solvents were tested: ACN/water (80:20, v/v) and ACN/water/FA (75:20:5, v/v/v; 79:20:1, v/v/v; 74:25:1, v/v/v). Using solvents without FA led to low recovery rates of, e.g., fumonisins, which have tricarboxylic side chains, while a high content of FA revealed poor recovery rates of acid-labile compounds like, e.g., benfuracarb or carbosulfan. An extraction solvent containing 1% FA showed the best recovery rates overall. Next, QuEChERS salts were applied after 20 min extraction for further sample purification. Comparison of recovery rates revealed the exclusion of a QuEChERS-based extraction method because low recoveries for a huge amount of compounds, such as polar mycotoxins deoxynivalenol-3-glucoside or nivalenol, were obtained. The use of sodium sulfate for water binding was also tested, but the approach was discarded due to lower recoveries and no improvements of the HILIC peak shapes. Furthermore, various syringe filters and dilutions were tested after extraction. Both transitions of tiocarbazil showed interferences when using syringe filters, even if the filters were rinsed before. The different syringe filters showed identical results for all analytes, wherefore Chromafil RC-20/15 filters were selected, and a higher LOQ for tiocarbazil was accepted. A dilution of the final extract with ACN (1:1) was chosen since the water content of the extract was too high for early eluting peaks in HILIC chromatography, and further sample purification was performed with this step.

Further optimization of the sample preparation process was carried out. For example, dilutions for matrix-matched samples to determine recovery rates were compared using plastic reaction vessels and screw-capped glass vials. Since carbamate esters such as desmedipham and phenmedipham had a short half-life in the used not acidified working solution and were no longer detectable after several hours in the mix, optimization of the working solution was implemented. Therefore, experiments were carried out to determine the extent to which the spiking mix must be acidified. Acidification with 0.05% and 0.1% FA showed that the analytes were stable for 24 h. Finally, a content of 0.05% acid was chosen because a higher FA content led to rapid hydrolysis of dimefox, while also dichlorvos showed a lower signal intensity. Even though poor results for the modified mycotoxin zearalenone-14,16-disulfate were obtained (recoveries < 10%), a simple extraction process using ACN/water/FA (79:20:1, v/v/v) as the optimum extraction solvent and a working solution containing 0.05% FA were finally chosen for the validation.

### Method validation

**Fig. 2:**
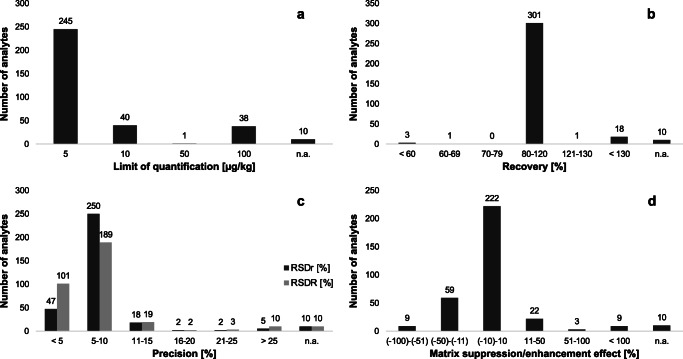
Validation data for pesticides at spiking level no. 7 in wheat. **a** Limits of quantification. **b** Recoveries (*n* = 6). **c** Repeatabilities (RSDr), within-laboratory reproducibilities (RSDR). **d** Matrix suppression/enhancement effects

The developed 2D-LC-MS/MS method was validated for wheat by spiking blank samples on multiple levels. The results for linearity are shown in ESM Table S5 and Table S6 for contaminants and pesticides, respectively. Good linearity was obtained for all analytes with an *R*^2^ > 0.96. Results for contaminants were slightly better than for pesticides. Based on S/N ratios, LOQs were calculated. Due to the relatively high dilution of analytes in 2D-LC setups, sensitivity is a critical factor in evaluating the method’s performance. Since the described 2D-LC application used a kind of solid phase extraction and concentration of the sample can be done, a negative effect on the sensitivity is not expected. As given in Table 3, LOQs for mycotoxins, plant growth regulators, and tropane alkaloids ranged from 1 to 300 μg/kg and were far below MLs set in European regulations and recommendations [7, 10, 16]. Significance differences in the LOQs between free and modified forms were observed for fumonisins and zearalenone, whereas modified forms revealed poorer sensitivities. LOQ data for pesticides are presented in Fig. 2. The LOQ was set at 5 μg/kg for 245 (73%) of the investigated analytes while 40 (12%) analytes reached a LOQ of 10 μg/kg, one (0.3%) analyte of 50 μg/kg, and 38 analytes (11%) of 100 μg/kg. For the majority of analytes, the LOQs were low enough to enforce the MRLs for pesticides established in the European Regulation [7].

**Table 3:** Limits of quantification (LOQ), recoveries (*R*_E_, *n* = 6), repeatabilities (RSDr), within-laboratory reproducibilities (RSDR), and matrix suppression/enhancement effects (MSE) for mycotoxins, plant growth regulators, and tropane alkaloids in wheat

Analyte	LOQ [μg/kg]	*R*_E_ (RSD) [%]^a^	RSDr [%]^a^	RSDR [%]^a^	MSE [%]^b^
Aflatoxin B1	1	90 (10)	11.5	6.94	− 13
Aflatoxin B2	1	91 (10)	10.2	14.4	− 5
Aflatoxin G1	1	108 (8)	10.8	10.7	18
Aflatoxin G2	1	94 (12)	9.32	12.3	− 1
Altenuene	20	94 (9)	12.1	9.24	7
Alternariol	1	98 (5)	8.33	11.1	− 12
Alternariol monomethyl ether	1	105 (5)	5.36	7.08	− 10
Citrinin	50	57 (6)	8.20	13.3	− 55
Deoxynivalenol	50	93 (7)	7.31	9.99	25
Deoxynivalenol-3-glucoside	50	87 (7)	7.87	6.61	− 25
15-Acetyldeoxynivalenol	50	85 (16)	12.4	10.1	− 8
3-Acetyldeoxynivalenol	50	107 (10)	11.8	13.7	− 12
Diacetoxyscirpenol	5	90 (7)	9.21	6.70	− 4
Fumonisin B1	10	79 (17)	12.9	105	221
Hydrolyzed fumonisin B1	100	91 (9)	8.73	8.44	− 1
Fumonisin B2	10	76 (12)	8.24	48.9	249
Hydrolyzed fumonisin B2	100	93 (5)	9.95	5.24	− 56
Fumonisin B3	10	89 (7)	6.67	49.1	296
Fusarenon X	300	104 (16)	10.7	11.7	− 2
HT-2 toxin	50	96 (6)	9.61	10.9	− 7
Neosolaniol	5	93 (9)	11.6	13.8	7
Nivalenol	200	99 (4)	6.69	6.74	− 49
Ochratoxin A	3	89 (6)	7.07	9.79	650
Sterigmatocystin	1	104 (8)	9.87	8.26	4
T-2 toxin	10	98 (7)	7.77	9.42	1
Tentoxin	5	101 (12)	9.54	12.8	− 12
Zearalenone	10	107 (5)	4.19	8.18	− 7
Zearalenone-14-glucoside	50	85 (8)	7.57	10.0	274
Zearalenone-14-sulfate	50	84 (8)	7.28	9.04	5
Zearalenone-14,16-disulfate	100	7 (10)	10.6	13.4	− 7
α-Zearalenol	10	102 (5)	5.24	7.25	− 5
α-Zearalenol-14-glucoside	50	100 (12)	13.3	15.7	1058
α-Zearalenol-14-sulfate	10	79 (7)	9.54	9.28	− 8
β-Zearalenol	10	97 (4)	8.11	8.92	− 8
β-Zearalenol-14-glucoside	50	80 (11)	16.0	16.5	1574
β-Zearalenol-14-sulfate	10	70 (8)	15.0	11.1	−2
Zearalanone	10	104 (6)	6.34	7.57	− 7
Zearalanone-14-glucoside	10	92 (11)	8.16	8.33	267
α-Zearalanol	100	101 (6)	6.18	7.80	− 11
β-Zearalanol	100	95 (9)	8.34	13.7	− 5
Chlormequat	5	91 (5)	7.40	3.60	− 58
Mepiquat	5	95 (4)	10.2	4.08	− 33
Atropine	5	103 (4)	11.5	6.52	− 57
Scopolamine	5	105 (3)	8.96	4.95	− 47

^a^Evaluated at spiking level of LOQ

^b^Evaluated at spiking level no. 7

Recovery rates were determined on multiple concentration levels. Results for contaminants and plant growth regulators evaluated at their respective LOQs are shown in Table 3. The lowest recoveries were assessed for zearalenone-14,16-disulfate and citrinin with values of 7% and 57%, respectively. The remaining analytes showed recoveries within the target range of 70–110%, and regulated analytes were within limits given in Commission Regulation (EC) no. 401/2006 [34] and the document N° SANTE/12682/2019 [35]. The results for pesticides are displayed in Fig. 2. A total of 301 (90%) analytes revealed recoveries evaluated at spiking level no. 7 (500 μg/kg for aminopyralid/dodine, 100 μg/kg for the remaining pesticides) within limits of 70–120% set in SANTE guidelines [35]. Acid-labile compounds displayed recoveries < 60%, while mainly carbamates showed values < 130%.

The repeatability and within-laboratory reproducibility were tested at different concentration levels. In Table 3, precision data for contaminants and plant growth regulators are shown at the lowest spiking point. Good results (RSDr/RSDR < 20%) for all analytes were reached except for fumonisins. Fumonisins B1, B2, and B3 revealed RSDr values (< 15%) within limits of Commission Regulation (EC) no. 401/2006 [34] but concerning RSDR data of 105% for fumonisin B1 and 48% for both fumonisins B2 and B3 (within limits of < 60%). The method was precise on the respective days but showed large fluctuations within the three days of processing. Correction by internal standards could support circumventing the problem of insufficient precision. However, this approach is not attainable when analyzing such a high number of analytes as in the presented method. Pesticide precision data are presented in Fig. 2. According to SANTE, values up to 20% are acceptable. Similar to the recovery results, most analytes reached these targets, more precisely 317 (95%) and 311 (93%) analytes for RSDr and RSDR, respectively. Analytes that showed higher recoveries, mostly carbamates, also displayed higher relative standard deviations.

The expanded measurement uncertainty was calculated at the respective LOQs. For contaminants and plant growth regulators, data ranged from 10 to 297% (see ESM Table S5). For pesticides, MU values varied between 8 and 250% (see ESM Table S6). Since the MU is determined based on precision data, fumonisins and carbamates showed significantly higher values. One possible reason for the high values of the MU of the carbamates may be the irreproducible degradation of carbamates into their respective metabolites. Carbamates can be metabolized to sulfones and sulfoxides. Neither the MU of the free carbamates nor the respective sulfones or sulfoxides revealed results within specified limits. Overall, 301 (91%) analytes reached the default criterion of < 50% established in SANTE document [35].

While matrix impacts the lifetime of columns and source of mass spectrometers, also precise quantification may be adversely influenced [42]. MEs have been investigated for all analytes. As displayed in Table 3, the matrix suppression/enhancement (MSE) effect of contaminants and plant growth regulators ranged highly from −58 to +1574%. Matrix suppression was mainly observed in the third phase of HILIC analysis. In comparison, strong matrix enhancement was observed for already as critically described fumonisins and multiply glucosylated zearalenone forms. For pesticides, MSE effects are subdivided into several groups (Fig. 2). MSE values ± 10% are considered to be not affected by matrix components. No matrix effects, observed for 222 (66%) analytes, may result from the advanced separation of analytes and matrix compounds using 2D-LC-MS/MS. While strong matrix suppression was determined for amitraz and amitraz-amidin, matrix enhancement was monitored particularly for early eluting analytes of the RP analysis. Overall, pronounced MEs were expected since LC-ESI-MS/MS was used. ESI sources are compared to other ionization modes such as atmospheric pressure chemical ionization (APCI) or atmospheric pressure photoionization (APPI), more prone to MEs [43, 44]. MEs constitute a major bottleneck in multi-analyte methods, where compensation by, e.g., stable isotope dilution assays (SIDA) or matrix-matched calibration (MMC) is only applicable in a limited manner. In comparison to other two-dimensional studies, reduced matrix effects were achieved for most analytes by optimal online clean-up in the second phase of 2D-LC and chromatographic separation of purified analytes eluting from the RP column in phase three [26, 27]. While Mahdjoubi et al. (2020) observed only signal suppression for investigated mycotoxins in cereal samples using the positive ESI mode [45], Juan et al. (2016) came to similar results revealing signal suppression for all mycotoxins except for ZEN and enniatins [46]. Comparing the matrix effects of mycotoxins presented here with a recently published LC-MS/MS method using the same mass spectrometer by Rausch et al. (2020) reveals slight differences for mentioned fumonisins and glucose-conjugated zearalenone forms [37]. The results suggest that the abnormally high matrix effects may have their origin in the 2D-LC setup. MEs for plant growth regulators and tropane alkaloids were assimilable with the literature [47–49].

Numerical values of LOQs, recoveries, precisions, and matrix effects for all pesticides are listed in ESM Tables S6, S7, and S8, respectively. Further recoveries and precisions for mycotoxins, plant growth regulators, and tropane alkaloids are given in ESM Table S9. In Table S10, a final overview of the validation results is presented. Analytes that are considered in-house validated are listed, while those analytes where validation parameters were outside the range are marked with their respective unsatisfactory validation parameter.

### Application to samples

The validated method was applied on 36 cereal samples (18 wheat, seven barley, three rice, five oats, one spelt, two rye samples). Due to especially co-eluting matrix components during HILIC analysis and thus analyte-dependent MEs, quantification was carried out using the standard addition technique. As shown in Table 4, samples were contaminated with eleven mycotoxins, one plant growth regulator, and 16 pesticides. For mycotoxins, *Alternaria* toxins, type B trichothecenes, and zearalenone were mostly detected in wheat, barley, rice, and oats. Besides deoxynivalenol-3-glucoside, no other modified mycotoxins were found. In two samples, ochratoxin A was analyzed in concentrations above the maximum levels set in Commission Regulation (EC) no. 1881/2006 [10]. While the plant growth regulator chlormequat was found in five samples (wheat and oats), pesticides revealed positive findings in 15 samples. The application of pesticides could be observed mainly in wheat, barley, and rice. The greatest number of pesticides was identified in two barley samples. While one rye sample contained diethyltoluamide, oats and spelt were free from residues. In general, the investigated cereals were often contaminated with different types of triazoles and piperonyl butoxide. The latter was detected in the highest concentrations up to 461 μg/kg. Teflubenzuron, tricyclazole, and triflumuron were quantified in concentrations higher than 10 μg/kg, which did not meet regulations established in Commission Regulation (EC) no. 396/2005 [7].

**Table 4:** Survey results of contaminants and pesticides in cereals

	Rate^a^ and range^b^ of contamination [μg/kg]					
Analyte	Wheat	Barley	Rice	Oats	Spelt	Rye
Alternariol	2/18 (1.1, 1.1)	4/7 (7.9–16)	1/3 (7)	3/5 (6.5–46)	1/1 (> LIN)^c^	
Alternariol monomethyl ether	2/18 (1.3, 4.7)	3/7 (2.2–3.3)	1/3 (13)	3/5 (1.1–5.7)	1/1 (> LIN)^c^	
Deoxynivalenol	1/18 (640)	3/7 (672–1034)	1/3 (153)	1/5 (171)		
Deoxynivalenol-3-glucoside	3/18 (60–259)	4/7 (111–252)				
HT-2 toxin		1/7 (59)		1/5 (52)		
Nivalenol		1/7 (453)		1/5 (521)		
Ochratoxin A	1/18 (10)			1/5 (5.1)		
Sterigmatocystin	1/18 (18)		2/3 (1.1, 3.4)	1/5 (> LIN)^c^		
T-2 toxin		1/7 (19)		1/5 (17)		
Tentoxin	6/18 (5.5–27)	4/7 (13–15)		3/5 (8.2–21)	1/1 (11)	2/2 (12, 24)
Zearalenone		2/7 (17, 22)	1/3 (38)	1/5 (21)	1/1 (25)	
Chlormequat	4/18 (6.7–213)			1/5 (23)		1/2 (34)
Azoxystrobin	2/18 (7.4, 9.9)		1/3 (5.4)			
Carbendazim		4/7 (17–44)				
Cyproconazole			1/3 (89)			
Diethyltoluamide						1/2 (27)
Diflubenzuron		2/7 (7.6, 7.7)				
Epoxiconazole	1/18 (5.7)	1/7 (5.7)	1/3 (16)			
Imidacloprid			1/3 (31)			
Malathion	1/18 (44)					
Piperonyl butoxide	3/18 (6.2–461)	4/7 (5.7–11)	1/3 (6.2)			
Propiconazole		4/7 (8.2–11)	1/3 (22)			
Pyraclostrobin		2/7 (5.5, 5.8)				
Tebuconazole	3/18 (17–42)	5/7 (11–27)	1/3 (20)			
Teflubenzuron			1/3 (11)			
Thiamethoxam			1/3 (7.5)			
Tricyclazole			1/3 (20)			
Triflumuron		4/7 (16–24)				

^a^Number of samples contaminated with the listed analyte/number of matrix samples analyzed

^b^Concentration range of contamination levels of the listed analyte

^c^Contamination level higher than linear range

Overall, wheat, barley, rice, and oats samples were contaminated mainly with at least one analyte. In contrast, spelt and rye were contaminated with only four and three substances, respectively. In conclusion, co-occurrence of both mycotoxins and pesticides was detected primarily in wheat and barley samples using the proposed method under the experimental conditions applied (see ESM Table S11). Five samples were free of investigated contaminants and residues. A total of 25% of the investigated mycotoxins, 50% of the plant growth regulators, and 4.8% of the pesticides were quantified in the cereals.

## Conclusion

Increased global crop production and the application of pesticides and fertilizers demand fast analytical methods to analyze multiple analyte classes. Therefore, a 2D-LC-MS/MS multi-method for quantifying 40 (modified) mycotoxins, two plant growth regulators, two tropane alkaloids, and 334 pesticides has been developed, validated, and applied to cereal samples. Simple sample preparation was the basis for an online clean-up using the 2D-LC setup. Matrix components are separated from analytes in the first dimension on a HILIC column. Unpolar substances elute directly and are saved on a trap column. Next, few highly polar analytes elute directly into the MS and are detected, while the majority of matrix compounds show still retention on the HILIC column. The remaining saved analytes are analyzed after switching the mobile phase composition in the second dimension on an RP column combination. The developed method showed overall good validation data for most analytes within limits set in Commission Decision (EC) no. 657/2002 [33] and document N° SANTE/12682/2019 [35]. A few analytes, more precisely fumonisins and a few groups of pesticides like carbamates, did not reach established criteria. Nevertheless, the results suggest that the developed analytical method along the fast sample preparation is a useful tool for high-throughput routine screening. Not only a broad range of pesticides are analyzed simultaneously but also both free and modified food- and feed-relevant mycotoxins, as well as plant growth regulators and tropane alkaloids. Most modified mycotoxins were included in a multi-method containing both pesticides and contaminants for the first time. Even though confirmatory analyses may be necessary from time to time, the method is time- and cost-saving by studying the co-occurrence of nearly 380 analytes in one analytical run. The analysis of real samples confirmed the natural occurrence of contaminants and the use of pesticides in agriculture. While mycotoxins revealed positive findings in all investigated cereal matrices, pesticides were mainly detected in wheat, barley, and rice. In a few samples, concentrations of ochratoxin A and three pesticides slightly exceeded maximum (residue) levels.

## Supplementary information

ESM 1 (PDF 770 kb)

## Abbreviations

1D-LC: One-dimensional liquid chromatography
2D-LC: Two-dimensional liquid chromatography
ACN: Acetonitrile
AFNH_4_: Ammonium formate
APCI: Atmospheric pressure chemical ionization
APPI: Atmospheric pressure photoionization
CE: Collision energy
CXP: Collision exit potential
DP: Declustering potential
EC: European Commission
ESI: Electrospray ionization
EU: European Union
FA: Formic acid
GC: Gas chromatography
HILIC: Hydrophilic interaction liquid chromatography
HPLC: High performance liquid chromatography
HRMS: High resolution mass spectrometry
IAC: Immune-affinity columns
ISTD: Internal standard
IUPAC: International Union of Pure and Applied Chemistry
LC-LC: Heart-cutting liquid chromatography
LCxLC: Comprehensive liquid chromatography
LLE: Liquid-liquid extraction
LOQ: Limit of quantification
ME: Matrix effect
MeOH: Methanol
ML: Maximum level
MRL: Maximum residue level
MSPD: Matrix solid phase dispersion
MS/MS: Tandem mass spectrometry
MU: Measurement uncertainty
*R*^2^: Coefficients of determination
*R*_E_: Recovery
RP: Reversed phase
RSD: Relative standard deviation
RSDr: Repeatability
RSDR: Within-laboratory reproducibility
RT: Retention time
sMRM: Scheduled multiple reaction mode
S/N: Signal-to-noise
SPE: Solid phase extraction
QuEChERS: Quick, easy, cheap, effective, rugged, safe
