# Supplementary material for: Development, validation, and application of a multi-method for the determination of mycotoxins, plant growth regulators, tropane alkaloids, and pesticides in cereals by two-dimensional liquid chromatography tandem mass spectrometry
Source: Anal Bioanal Chem. 2021 Mar 13;413(11):3041–54. doi: 10.1007/s00216-021-03239-1 (PMC8044062; doi:10.1007/s00216-021-03239-1)
Supplement: Supplementary file 1 — (PDF 770 kb) [file 216_2021_3239_MOESM1_ESM.pdf]

Supporting Information – Analytical and Bioanalytical Chemistry

Development, Validation, and Application of a Multi-Method for the Determination of Mycotoxins, Plant Growth Regulators, Tropane Alkaloids, and Pesticides in Cereals by Two-Dimensional Liquid Chromatography Tandem Mass Spectrometry

Ann-Kristin RAUSCH[a,b*], Robert BROCKMEYER[b], Tanja SCHWERDTLE[ac]

[a] Department of Food Chemistry, Institute of Nutritional Science, University of Potsdam, Arthur-Scheunert-Allee 114-116, 14558 Nuthetal, Germany

[b] Eurofins SOFIA GmbH, Rudower Chaussee 29, 12489 Berlin, Germany

[c] General Federal Institute for Risk Assessment, Max-Dohrn-Straße 8-10, 10589 Berlin, Germany

[*] Corresponding author: ann-kristin.rausch@uni-potsdam.de

E-Mail addresses:

robertbrockmeyer@eurofins.de (R. Brockmeyer), tanja.schwerdtle@uni-potsdam.de (T. Schwerdtle)

Supporting Information Description

Tables of concentration of analytes in multi-standard working solution; concentration of internal standards in working solution; MS/MS parameters and chromatographic retention time; spiking levels of validation; evaluated levels, calibration range, R^2, and measurement uncertainty for contaminants; evaluated levels, calibration range, R^2, and measurement uncertainty for pesticides; recoveries, repeatabilities, reproducibilities for pesticides; matrix suppression/enhancement effects for pesticides; recoveries, repeatabilities, reproducibilities for contaminants; overall validation results; detailed survey results.

Supplementary Material

Table S1 Concentration of Analytes in Combined Multi-Standard Working Solution.

Analyte	Concentration [µg/mL]
aflatoxin B1	1
aflatoxin B2	1
aflatoxin G1	1
aflatoxin G2	1
altenuene	20
alternariol	1
alternariol monomethyl ether	1
citrinin	50
deoxynivalenol	50
15-acetyldeoxynivalenol	50
3-acetyldeoxynivalenol	50
diacetoxyscirpenol	5
fumonisin B1	10
hydrolyzed fumonisin B1	100
fumonisin B2	10
hydrolyzed fumonisin B2	100
fumonisin B3	10
fusarenon X	200
HT-2 toxin	50
neosolaniol	5
nivalenol	200
ochratoxin A	3
sterigmatocystin	1
T-2 toxin	10
tentoxin	5
zearalenone	10
zearalenone-14-glucoside	50

Analyte	Concentration [µg/mL]
zearalenone-14-sulfate	50
zearalenone-14,16-disulfate	100
α-zearalenol	10
α-zearalenol-14-glucoside	50
α-zearalenol-14-sulfate	10
β-zearalenol	10
β-zearalenol-14-glucoside	50
β-zearalenol-14-sulfate	10
zearalanone	50
zearalanone-14-glucoside	10
α-zearalanol	100
β-zearalanol	100
chlormequat	5
mepiquat	5
atropine	5
scopolamine	5
pesticide mix “red”, per compound	5
pesticide mix “yellow”, per compound	5
pesticide mix “orange”, per compound	5
pesticide mix “purple”, per compound	5
pesticide mix “blue”, per compound	5
pesticide mix “green”, per compound	5
pesticide mix “black 1”, per compound	5
pesticide mix “black 2”, per compound	5
daminozide	5
aminopyralid	50
dodine	50

Table S2 Concentration of Internal Standards (ISTD) in Combined Working Solution.

ISTD	Concentration [µg/mL]
zearalenone dimethyl ether-d6	5
chlormequat-d4	5
mepiquat-d4	5
carbendazim-d4	2.5
imidacloprid-d4	2.5
diazinon-d10	5
diuron-d6	2.5

Table S3 MS/MS Parameters [Declustering Potential (DP), Collision Energy (CE), Collision Exit Potential (CXP)] and Retention Time (RT) for 378 Analytes and 8 Internal Standards; Quant.: Quantifier.

Analyte	ESI mode	Quant. ion	Precursor ion [m/z]	Product ion [m/z]	DP [V]	CE [V]	CXP [V]	RT [min]
carbendazim-d4	ESI +		196	164	71	27	12	12.04
carbendazim-d4	ESI +		196	136	71	45	22	12.07
diuron-d6	ESI +		239	78	30	35	8	13.27
diuron-d6	ESI +		241	78	30	35	8	13.33
diazinon-d10	ESI +		315	154	31	33	14	16.24
diazinon-d10	ESI +		315	170	31	33	14	16.30
imidacloprid-d4	ESI +		260	179	36	21	17	12.16
imidacloprid-d4	ESI +		260	213	51	21	15	12.19
propamocarb-d7	ESI +		196	103	21	27	16	3.67
propamocarb-d7	ESI +		196	151	21	21	12	3.66
zearalenone dimethyl ether-d6	ESI +		353	221	71	25	26	17.23
zearalenone dimethyl ether-d6	ESI +		353	317	71	19	42	17.25
chlormequat-d4	ESI +		126	58	41	43	6	3.85
mepiquat-d4	ESI +		117	61	16	36	12	5.09
aflatoxin B1	ESI +		313	285	191	31	24	17.24
aflatoxin B1	ESI +	x	313	241	191	49	22	17.24
aflatoxin B2	ESI +	x	315	287	181	37	32	16.56
aflatoxin B2	ESI +		315	259	181	41	20	16.56
aflatoxin G1	ESI +	x	329	243	74	38	13	16.26
aflatoxin G1	ESI +		329	311	61	31	10	16.25
aflatoxin G2	ESI +	x	331	189	11	59	16	15.48
aflatoxin G2	ESI +		331	245	64	42	13	15.49
altenuene	ESI +	x	293	239	76	29	18	12.83
altenuene	ESI +		293	257	76	21	30	12.83
3-acetyldeoxynivalenol	ESI +		339	203	75	25	10	12.03
3-acetyldeoxynivalenol	ESI +	x	339	231	75	22	10	12.04
15-acetyldeoxynivalenol	ESI +		356	321	16	17	24	11.98
15-acetyldeoxynivalenol	ESI +	x	356	137	16	21	12	11.98
deoxynivalenol	ESI +		297	203	100	20	10	11.10
deoxynivalenol	ESI +	x	297	249	86	15	26	11.10
deoxynivalenol-3-glucoside	ESI +		459	297	51	13	10	4.06
deoxynivalenol-3-glucoside	ESI +		476	297	46	19	10	4.06
diacetoxyscirpenol	ESI +	x	384	307	36	17	10	13.22
diacetoxyscirpenol	ESI +		384	247	36	21	8	13.22
fumonisin B1	ESI +		722	334	26	55	16	7.30
fumonisin B1	ESI +	x	722	352	26	49	20	7.29
fumonisin B2	ESI +	x	706	336	16	51	12	7.24
fumonisin B2	ESI +		706	318	16	53	10	7.24
fumonisin B3	ESI +	x	706	336	31	49	20	7.24
fumonisin B3	ESI +		706	354	31	45	32	7.24
fusarenon X	ESI +	x	372	229	20	20	10	11.49

Analyte	ESI mode	Quant. ion	Precursor ion [m/z]	Product ion [m/z]	DP [V]	CE [V]	CXP [V]	RT [min]
fusarenon X	ESI +		372	175	51	25	18	11.49
hydrolyzed fumonisin B1	ESI +	x	406	370	111	27	14	7.24
hydrolyzed fumonisin B1	ESI +		406	388	111	25	14	7.24
hydrolyzed fumonisin B2	ESI +		390	372	61	27	12	4.59
hydrolyzed fumonisin B2	ESI +	x	390	336	61	33	20	4.59
hydrolyzed fumonisin B3	ESI +		390	354	51	27	24	4.59
hydrolyzed fumonisin B3	ESI +		390	336	51	31	22	4.59
HT-2 toxin	ESI +	x	442	263	41	17	13	13.56
HT-2 toxin	ESI +		442	215	41	15	13	13.57
neosolaniol	ESI +		400	185	61	27	16	11.64
neosolaniol	ESI +	x	400	215	61	25	18	11.64
sterigmatocystin	ESI +		325	281	41	51	18	17.67
sterigmatocystin	ESI +	x	325	310	41	35	28	17.67
T-2 toxin	ESI +		484	215	76	25	26	14.85
T-2 toxin	ESI +	x	484	245	51	19	13	14.85
tentoxin	ESI +		415	58	131	73	8	14.12
tentoxin	ESI +	x	415	312	131	29	30	14.12
zearalenone-14-glucoside	ESI +		498	319	36	19	10	14.13
zearalenone-14-glucoside	ESI +		498	283	36	35	10	14.13
zearalenone-14-sulfate	ESI +	x	416	301	96	25	24	13.41
zearalenone-14-sulfate	ESI +		416	319	96	15	28	13.41
α-zearalenol-14-glucoside	ESI +		500	285	41	31	10	13.26
α-zearalenol-14-glucoside	ESI +	x	500	321	41	17	10	13.26
β-zearalenol-14-glucoside	ESI +		500	303	66	23	10	12.47
β-zearalenol-14-glucoside	ESI +	x	500	321	66	17	24	12.47
zearalanone	ESI +		321	303	96	19	10	15.98
zearalanone	ESI +	x	321	115	96	73	14	15.98
zearalanone-14-glucoside	ESI +	x	500	303	71	33	10	14.11
zearalanone-14-glucoside	ESI +		500	321	71	17	10	14.11
α-zearalanol	ESI +		323	305	61	13	30	14.60
α-zearalanol	ESI +	x	323	149	61	35	12	14.59
β-zearalanol	ESI +		323	189	56	31	20	13.82
β-zearalanol	ESI +	x	323	305	56	11	30	13.82
atropine	ESI +	x	290	124	80	30	10	3.55
atropine	ESI +		290	93	80	50	10	3.56
scopolamine	ESI +	x	305	156	80	24	10	3.39
scopolamine	ESI +		305	138	80	32	10	3.39
chlormequat	ESI +	x	122	58	41	43	6	3.87
chlormequat	ESI +		124	58	41	43	6	3.87
mepiquat	ESI +	x	114	98	40	36	12	5.08
mepiquat	ESI +		114	58	51	37	10	5.09
3,4,5-trimethacarb	ESI +		194	122	81	35	6	13.72
3,4,5-trimethacarb	ESI +	x	194	137	80	15	6	13.72
acephate	ESI +	x	184	143	16	13	8	11.00
acephate	ESI +		184	95	16	31	8	11.00
acetamiprid	ESI +	x	223	126	50	27	6	12.51
acetamiprid	ESI +		223	90	26	49	10	12.51

Analyte	ESI mode	Quant. ion	Precursor ion [m/z]	Product ion [m/z]	DP [V]	CE [V]	CXP [V]	RT [min]
acetochlor	ESI +	x	270	224	31	15	12	15.83
acetochlor	ESI +		272	226	31	15	12	15.82
alachlor	ESI +	x	270	162	64	29	9	15.90
alachlor	ESI +		270	238	64	15	10	15.90
aldicarb	ESI +	x	208	116	20	13	6	12.32
aldicarb	ESI +		208	89	21	21	6	12.33
aldicarb-sulfoxide	ESI +	x	224	132	36	15	12	11.09
aldicarb-sulfoxide	ESI +		224	89	36	25	10	11.09
aldoxycarb	ESI +		240	148	20	19	8	11.38
aldoxycarb	ESI +	x	240	166	20	17	10	11.37
ametoctradin	ESI +		276	149	91	51	12	16.25
ametoctradin	ESI +	x	276	176	91	51	4	16.25
ametryn	ESI +	x	228	186	50	25	10	14.17
ametryn	ESI +		228	96	46	35	10	14.17
amidosulfuron	ESI +		370	218	36	33	16	12.39
amidosulfuron	ESI +	x	370	261	31	25	16	12.38
aminocarb	ESI +		209	137	31	35	12	13.18
aminocarb	ESI +	x	209	152	30	19	8	13.18
aminopyralid	ESI +		209	163	41	29	12	5.16
aminopyralid	ESI +	x	209	191	41	19	14	5.16
amitraz	ESI +		294	122	51	41	10	18.40
amitraz	ESI +	x	294	163	30	21	8	18.40
amitraz-amide	ESI +	x	150	105	61	25	8	12.42
amitraz-amide	ESI +		150	107	61	29	10	12.42
amitraz-amidin	ESI +	x	163	106	36	45	10	2.81
amitraz-amidin	ESI +		163	77	36	55	10	2.80
ancymidol	ESI +	x	257	135	36	37	12	13.21
ancymidol	ESI +		257	92	36	73	16	13.21
atrazine	ESI +		216	104	21	37	10	13.20
atrazine	ESI +	x	216	174	35	25	10	13.20
atrazine-desethyl-desisopropyl	ESI +		188	104	70	33	6	11.87
atrazine-desethyl-desisopropyl	ESI +	x	188	146	36	25	12	11.88
avermectin B1a	ESI +	x	890	567	61	19	8	18.79
avermectin B1a	ESI +		890	305	51	35	14	18.79
avermectin B1b	ESI +	x	877	291	60	35	16	18.65
avermectin B1b	ESI +		877	553	61	21	16	18.65
azaconazole	ESI +		300	159	41	41	14	14.57
azaconazole	ESI +	x	300	231	41	25	18	14.57
azamethiphos	ESI +	x	325	183	16	21	10	13.92
azamethiphos	ESI +		325	112	16	40	10	13.92
aziprotryne	ESI +		226	125	21	19	8	14.56
aziprotryne	ESI +	x	226	156	21	21	10	14.56
azoxystrobin	ESI +	x	404	344	31	29	12	16.60
azoxystrobin	ESI +		404	372	50	19	20	16.59
benalaxyl	ESI +		326	148	36	31	14	16.91
benalaxyl	ESI +	x	326	208	21	21	10	16.91
bendiocarb	ESI +	x	224	109	11	21	10	12.87

Analyte	ESI mode	Quant. ion	Precursor ion [m/z]	Product ion [m/z]	DP [V]	CE [V]	CXP [V]	RT [min]
bendiocarb	ESI +		224	167	20	13	8	12.86
benfuracarb	ESI +	x	411	190	56	17	14	17.95
benfuracarb	ESI +		411	195	15	31	10	17.95
benodanil	ESI +		324	231	46	33	20	13.86
benodanil	ESI +	x	324	76	76	83	2	13.86
benomyl	ESI +	x	291	160	20	45	14	16.55
benomyl	ESI +		291	192	11	17	16	16.55
bensulfuron-methyl	ESI +		411	119	41	51	12	16.03
bensulfuron-methyl	ESI +	x	411	149	65	27	8	16.03
benthiavalicarb-isopropyl	ESI +	x	382	180	50	35	10	15.03
benthiavalicarb-isopropyl	ESI +		382	197	86	27	14	15.03
bitertanol	ESI +		338	269	36	15	16	16.44
bitertanol	ESI +	x	338	70	15	25	10	16.43
boscalid	ESI +		343	140	96	27	8	15.23
boscalid	ESI +	x	343	307	75	27	18	15.23
bromacil	ESI +	x	261	205	87	19	10	12.61
bromacil	ESI +		263	207	87	19	10	12.62
bromuconazole	ESI +	x	378	159	66	37	8	16.39
bromuconazole	ESI +		378	70	66	35	10	16.38
bupirimate	ESI +		317	108	51	35	6	16.24
bupirimate	ESI +	x	317	166	45	33	8	16.24
buprofezin	ESI +	x	306	116	31	21	6	17.45
buprofezin	ESI +		306	201	26	17	16	17.45
butachlor	ESI +		312	162	61	31	14	17.59
butachlor	ESI +	x	312	238	61	15	8	17.58
butocarboxim	ESI +	x	208	116	15	9	4	12.32
butocarboxim	ESI +		208	75	26	17	14	12.32
butocarboxim-sulfoxide	ESI +	x	207	132	20	11	6	10.96
butocarboxim-sulfoxide	ESI +		207	75	36	19	14	10.91
butoxycarboxim	ESI +	x	223	106	31	15	8	11.35
butoxycarboxim	ESI +		240	106	20	19	4	11.35
buturon	ESI +	x	237	84	55	21	4	13.36
buturon	ESI +		237	99	51	63	8	13.36
cadusafos	ESI +	x	271	159	90	19	9	16.46
cadusafos	ESI +		271	215	90	12	10	16.46
carbaryl	ESI +		202	127	95	43	7	13.20
carbaryl	ESI +	x	202	145	95	16	7	13.19
carbendazim	ESI +		192	132	16	41	2	12.03
carbendazim	ESI +	x	192	160	31	23	10	12.03
carbofuran	ESI +		222	123	25	29	6	13.05
carbofuran	ESI +	x	222	165	25	17	8	13.05
carbofuran-3-hydroxy	ESI +	x	238	163	16	23	14	11.80
carbofuran-3-hydroxy	ESI +		238	181	56	17	12	11.80
carbosulfan	ESI +	x	381	118	50	25	6	18.77
carbosulfan	ESI +		381	160	61	21	14	18.77
carboxin	ESI +	x	236	143	40	21	8	13.52
carboxin	ESI +		236	93	56	47	8	13.51

Analyte	ESI mode	Quant. ion	Precursor ion [m/z]	Product ion [m/z]	DP [V]	CE [V]	CXP [V]	RT [min]
chlorantraniliprole	ESI +		484	286	51	19	10	14.79
chlorantraniliprole	ESI +	x	484	453	51	27	10	14.79
chlorbromuron	ESI +		293	182	65	23	10	14.24
chlorbromuron	ESI +	x	293	204	56	29	14	14.24
chlorfluazuron	ESI +		540	158	71	29	12	18.25
chlorfluazuron	ESI +	x	540	383	70	31	12	18.25
chloridazon	ESI +	x	222	104	81	33	20	12.01
chloridazon	ESI +		222	92	70	35	4	12.00
chloroxuron	ESI +	x	291	218	66	33	10	15.14
chloroxuron	ESI +		291	72	65	41	4	15.15
chlorpropham	ESI +		214	154	31	25	14	14.17
chlorpropham	ESI +	x	214	172	44	12	7	14.17
chlorsulfuron	ESI +	x	358	141	65	23	6	12.46
chlorsulfuron	ESI +		358	167	51	25	12	12.47
chlortoluron	ESI +	x	213	72	31	31	6	13.12
chlortoluron	ESI +		215	72	31	31	6	13.12
cinidon-ethyl	ESI +	x	394	348	56	27	10	19.05
cinidon-ethyl	ESI +		394	77	56	89	14	19.05
cinosulfuron	ESI +		414	157	36	31	10	13.75
cinosulfuron	ESI +	x	414	183	50	23	10	13.75
clethodim	ESI +	x	360	164	44	25	8	16.63
clethodim	ESI +		360	268	44	20	8	16.63
clodinafop-propargyl	ESI +	x	350	266	55	21	14	16.86
clodinafop-propargyl	ESI +		350	91	41	41	10	16.86
clofentezine	ESI +	x	303	102	36	53	2	17.22
clofentezine	ESI +		303	138	60	21	6	17.22
clomazone	ESI +	x	240	125	40	27	6	14.64
clomazone	ESI +		240	89	40	65	4	14.64
clothianidin	ESI +	x	250	132	26	27	12	11.70
clothianidin	ESI +		250	169	26	19	14	11.70
cyanazine	ESI +	x	241	104	33	36	9	12.59
cyanazine	ESI +		241	214	55	23	12	12.59
cyazofamid	ESI +	x	325	108	36	19	6	16.45
cyazofamid	ESI +		325	261	56	15	14	16.45
cymoxanil	ESI +		199	111	16	25	2	12.18
cymoxanil	ESI +	x	199	128	36	13	10	12.18
cyproconazole	ESI +		292	125	46	39	8	14.77
cyproconazole	ESI +	x	292	70	46	33	10	14.76
cyprodinil	ESI +	x	226	108	46	37	10	16.05
cyprodinil	ESI +		226	77	81	63	10	16.05
cyprofuram	ESI +	x	280	69	81	23	12	14.29
cyprofuram	ESI +		282	69	81	27	10	14.28
cyromazine	ESI +	x	167	60	56	29	10	3.24
cyromazine	ESI +		167	85	56	27	6	3.24
daminozide	ESI +		161	61	48	19	4	3.75
daminozide	ESI +	x	161	143	48	17	12	3.75
demeton	ESI +	x	259	61	11	45	4	14.53

Analyte	ESI mode	Quant. ion	Precursor ion [m/z]	Product ion [m/z]	DP [V]	CE [V]	CXP [V]	RT [min]
demeton	ESI +		259	89	11	15	6	14.53
demeton-S-methyl	ESI +		231	61	26	45	12	13.25
demeton-S-methyl	ESI +	x	231	89	36	13	16	13.25
demeton-S-methyl-sulfone	ESI +		263	109	91	37	6	11.64
demeton-S-methyl-sulfone	ESI +	x	263	169	75	21	8	11.65
desmedipham	ESI +		318	154	21	33	10	14.15
desmedipham	ESI +	x	318	182	31	19	12	14.15
desmetryn	ESI +	x	214	172	66	25	12	13.47
desmetryn	ESI +		214	82	66	47	6	13.46
diazinon	ESI +	x	305	169	35	29	8	16.32
diazinon	ESI +		305	97	26	49	16	16.33
dichlorvos	ESI +	x	221	109	101	25	13	12.58
dichlorvos	ESI +		221	127	101	23	7	12.58
diclobutrazol	ESI +		328	159	56	51	10	15.25
diclobutrazol	ESI +	x	328	70	56	49	12	15.24
diethofencarb	ESI +	x	268	180	26	23	10	14.38
diethofencarb	ESI +		268	226	45	15	12	14.38
diethyltoluamide	ESI +	x	192	119	61	27	8	14.01
diethyltoluamide	ESI +		192	91	61	43	6	14.01
difenoconazole	ESI +		406	337	41	24	10	17.60
difenoconazole	ESI +	x	406	251	55	37	14	17.59
difenoxuron	ESI +	x	287	123	50	25	6	14.42
difenoxuron	ESI +		287	72	16	47	6	14.42
diflubenzuron	ESI +		311	141	112	44	8	15.62
diflubenzuron	ESI +	x	311	158	112	19	9	15.63
diflufenican	ESI +	x	395	246	66	49	6	17.01
diflufenican	ESI +		395	266	70	33	8	17.01
dimefox	ESI +	x	155	110	38	25	8	11.72
dimefox	ESI +		155	135	38	24	8	11.73
dimefuron	ESI +	x	339	167	75	29	8	14.29
dimefuron	ESI +		339	72	61	48	6	14.29
dimethenamid	ESI +	x	276	168	16	33	8	15.13
dimethenamid	ESI +		276	244	31	23	20	15.13
dimethoate	ESI +		230	125	68	27	7	11.94
dimethoate	ESI +	x	230	199	68	12	8	11.94
dimethomorph	ESI +		388	165	51	49	10	16.24
dimethomorph	ESI +	x	388	301	60	27	18	16.23
dimetilan	ESI +		241	196	66	15	10	12.42
dimetilan	ESI +	x	241	72	80	25	4	12.41
dimoxystrobin	ESI +		327	116	21	21	12	16.35
dimoxystrobin	ESI +	x	327	205	21	13	12	16.35
dinotefuran	ESI +		203	129	51	17	8	11.28
dinotefuran	ESI +	x	203	157	51	11	10	11.28
disulfoton	ESI +		275	61	41	43	12	16.77
disulfoton	ESI +	x	275	89	20	17	4	16.78
disulfoton-sulfone	ESI +		307	115	41	31	8	14.35
disulfoton-sulfone	ESI +	x	307	171	41	17	20	14.34

Analyte	ESI mode	Quant. ion	Precursor ion [m/z]	Product ion [m/z]	DP [V]	CE [V]	CXP [V]	RT [min]
disulfoton-sulfoxide	ESI +		291	157	21	31	14	14.08
disulfoton-sulfoxide	ESI +	x	291	185	21	19	14	14.08
diuron	ESI +	x	233	72	66	31	10	13.29
diuron	ESI +		235	72	41	37	6	13.29
dodine	ESI +	x	228	43	101	49	6	2.54
dodine	ESI +		228	57	101	39	4	2.53
emamectin B1a	ESI +	x	887	158	51	49	14	18.48
emamectin B1a	ESI +		886	82	96	119	6	18.47
emamectin B1b	ESI +		873	158	51	49	14	18.30
emamectin B1b	ESI +	x	873	82	116	107	6	18.30
epoxiconazole	ESI +		330	101	50	63	4	16.07
epoxiconazole	ESI +	x	330	75	21	91	6	16.07
ethiofencarb	ESI +		226	107	30	21	4	13.31
ethiofencarb	ESI +	x	226	169	31	11	13	13.31
ethiofencarb-sulfone	ESI +	x	275	107	25	25	6	11.79
ethiofencarb-sulfone	ESI +		275	201	36	17	18	11.79
ethiofencarb-sulfoxide	ESI +	x	242	107	55	23	4	11.74
ethiofencarb-sulfoxide	ESI +		242	185	51	23	20	11.74
ethiprole	ESI +	x	397	255	86	51	20	14.24
ethiprole	ESI +		397	351	86	31	20	14.24
ethofumesate	ESI +		287	121	51	23	10	15.00
ethofumesate	ESI +	x	287	259	51	15	14	15.00
ethofumesate-2-keto	ESI +		257	149	61	31	4	13.20
ethofumesate-2-keto	ESI +	x	257	177	61	23	12	13.20
ethoprofos	ESI +		243	131	36	29	10	15.37
ethoprofos	ESI +	x	243	173	36	21	14	15.36
etofenprox	ESI +		394	107	16	53	10	19.31
etofenprox	ESI +	x	394	135	26	31	8	19.31
etoxazole	ESI +	x	361	113	111	83	22	18.11
etoxazole	ESI +		360	141	80	37	8	18.12
famoxadone	ESI +		392	238	11	25	14	17.20
famoxadone	ESI +	x	392	331	11	15	10	17.20
fenamidone	ESI +		312	236	66	21	16	15.03
fenamidone	ESI +	x	312	92	66	33	6	15.03
fenamiphos	ESI +	x	304	202	46	49	18	15.66
fenamiphos	ESI +		304	217	46	33	12	15.66
fenamiphos-sulfone	ESI +	x	336	107	86	81	10	13.37
fenamiphos-sulfone	ESI +		336	108	86	49	8	13.37
fenamiphos-sulfoxide	ESI +		320	108	81	55	8	13.20
fenamiphos-sulfoxide	ESI +	x	320	156	81	43	14	13.21
fenarimol	ESI +		331	139	51	51	12	15.45
fenarimol	ESI +	x	331	268	61	31	8	15.45
fenazaquin	ESI +		307	147	41	25	8	18.58
fenazaquin	ESI +	x	307	161	65	31	8	18.59
fenbuconazole	ESI +		337	125	55	37	6	16.31
fenbuconazole	ESI +	x	337	70	61	33	4	16.31
fenhexamid	ESI +		302	55	66	59	2	14.91

Analyte	ESI mode	Quant. ion	Precursor ion [m/z]	Product ion [m/z]	DP [V]	CE [V]	CXP [V]	RT [min]
fenhexamid	ESI +	x	302	97	100	33	4	14.90
fenobucarb	ESI +	x	208	152	31	13	10	13.82
fenobucarb	ESI +		208	95	51	21	8	13.82
fenoxaprop-P-ethyl	ESI +		362	121	56	37	10	17.69
fenoxaprop-P-ethyl	ESI +	x	362	288	60	23	16	17.70
fenoxycarb	ESI +	x	302	116	81	17	6	16.22
fenoxycarb	ESI +		302	88	80	29	4	16.22
fenpiclonil	ESI +	x	237	202	26	31	14	14.07
fenpiclonil	ESI +		254	202	16	35	10	14.06
fenpropidin	ESI +		274	147	65	37	8	15.86
fenpropidin	ESI +	x	274	117	66	75	8	15.86
fenpropimorph	ESI +		304	117	81	71	6	16.95
fenpropimorph	ESI +	x	304	147	60	39	8	16.95
fenpyroximate	ESI +		422	135	16	41	6	18.83
fenpyroximate	ESI +	x	422	366	26	23	20	18.83
fensulfothion	ESI +		309	253	76	23	26	14.75
fensulfothion	ESI +	x	309	281	76	19	30	14.75
fensulfothion-PO-sulfone	ESI +	x	309	253	81	25	18	12.88
fensulfothion-PO-sulfone	ESI +		309	281	81	19	30	12.87
fensulfothion-PO-sulfoxide	ESI +		293	115	61	35	22	14.21
fensulfothion-PO-sulfoxide	ESI +	x	293	97	61	51	18	14.22
fensulfothion-PS-sulfone	ESI +		325	191	61	33	12	14.98
fensulfothion-PS-sulfone	ESI +	x	325	269	61	23	16	14.98
fenthion	ESI +	x	279	169	76	25	18	16.71
fenthion	ESI +		279	247	71	25	18	16.71
fenthion-oxon	ESI +	x	263	216	71	29	16	14.72
fenthion-oxon	ESI +		263	231	71	21	18	14.72
fenthion-PO-sulfone	ESI +		312	295	16	15	8	12.47
fenthion-PO-sulfone	ESI +	x	312	104	16	41	6	12.47
fenthion-PO-sulfoxide	ESI +	x	279	104	66	41	6	12.33
fenthion-PO-sulfoxide	ESI +		279	264	61	27	18	12.33
fenthion-PS-sulfone	ESI +	x	311	109	91	39	6	14.44
fenthion-PS-sulfone	ESI +		311	125	91	31	8	14.44
fenthion-PS-sulfoxide	ESI +		295	109	86	47	6	14.25
fenthion-PS-sulfoxide	ESI +	x	295	280	86	25	20	14.25
fenuron	ESI +		165	120	21	23	10	11.83
fenuron	ESI +	x	165	72	35	27	10	11.84
flazasulfuron	ESI +	x	408	182	55	25	10	14.19
flazasulfuron	ESI +		408	83	41	65	10	14.19
flonicamid	ESI +	x	230	148	61	37	10	11.43
flonicamid	ESI +		230	203	61	25	12	11.44
florasulam	ESI +	x	360	129	85	29	6	13.00
florasulam	ESI +		377	129	51	35	12	13.00
fluazifop-P-butyl	ESI +		384	282	65	27	16	17.49
fluazifop-P-butyl	ESI +	x	384	328	61	21	16	17.50
fluazuron	ESI +	x	506	349	61	31	10	17.88
fluazuron	ESI +		508	351	66	31	10	17.88

Analyte	ESI mode	Quant. ion	Precursor ion [m/z]	Product ion [m/z]	DP [V]	CE [V]	CXP [V]	RT [min]
flucycloxuron	ESI +		484	132	71	47	22	18.50
flucycloxuron	ESI +	x	484	289	31	17	10	18.50
fludioxonil	ESI +		266	158	56	46	8	14.30
fludioxonil	ESI +	x	266	229	56	19	8	14.30
flufenacet	ESI +		364	152	31	27	8	15.84
flufenacet	ESI +	x	364	194	25	17	10	15.84
flufenoxuron	ESI +		489	141	106	57	6	17.92
flufenoxuron	ESI +	x	489	158	100	27	8	17.92
fluometuron	ESI +		233	160	36	37	8	12.80
fluometuron	ESI +	x	233	72	46	37	4	12.80
fluopicolide	ESI +	x	383	173	71	31	14	15.29
fluopicolide	ESI +		385	175	36	35	14	15.28
flurochloridone	ESI +	x	312	292	61	31	8	15.51
flurochloridone	ESI +		312	89	71	37	8	15.51
flurprimidol	ESI +	x	313	269	86	49	16	14.24
flurprimidol	ESI +		313	270	86	33	16	14.25
flusilazole	ESI +		316	165	71	41	28	16.12
flusilazole	ESI +	x	316	247	50	25	14	16.12
flutriafol	ESI +		302	109	41	43	10	13.69
flutriafol	ESI +	x	302	123	55	39	6	13.69
formetanate	ESI +		222	65	46	63	12	3.15
formetanate	ESI +	x	222	165	21	23	10	3.16
fosthiazate	ESI +		284	104	75	27	4	13.89
fosthiazate	ESI +	x	284	228	81	15	12	13.89
fuberidazole	ESI +	x	185	157	40	31	8	12.53
fuberidazole	ESI +		185	65	26	59	4	12.53
furathiocarb	ESI +		383	195	65	23	10	17.97
furathiocarb	ESI +	x	383	252	46	19	12	17.97
halofenozide	ESI +	x	331	275	15	11	10	14.27
halofenozide	ESI +		331	105	11	23	8	14.28
haloxyfop	ESI +	x	362	288	105	35	11	13.60
haloxyfop	ESI +		362	316	105	24	11	13.60
haloxyfop-2-ethoxyethyl	ESI +		434	288	81	35	12	17.52
haloxyfop-2-ethoxyethyl	ESI +	x	434	316	81	25	12	17.52
haloxyfop-methyl	ESI +		376	288	86	33	12	16.98
haloxyfop-methyl	ESI +	x	376	316	100	23	18	16.98
hexaconazole	ESI +		314	70	35	48	11	15.52
hexaconazole	ESI +	x	316	70	35	50	8	15.51
hexaflumuron	ESI +		478	141	53	62	6	17.10
hexaflumuron	ESI +	x	478	158	53	29	6	17.10
hexazinone	ESI +	x	253	171	30	21	8	13.57
hexazinone	ESI +		253	71	71	47	12	13.57
hexythiazox	ESI +		353	168	56	33	10	18.21
hexythiazox	ESI +	x	353	228	26	23	6	18.21
imazalil	ESI +	x	297	159	41	31	10	16.26
imazalil	ESI +		297	201	86	25	18	16.26
imibenconazole	ESI +	x	411	125	36	47	10	17.96

Analyte	ESI mode	Quant. ion	Precursor ion [m/z]	Product ion [m/z]	DP [V]	CE [V]	CXP [V]	RT [min]
imibenconazole	ESI +		413	125	36	39	10	17.95
imidacloprid	ESI +		256	175	85	27	9	12.18
imidacloprid	ESI +	x	256	209	85	22	10	12.17
indoxacarb	ESI +		528	203	76	51	15	17.76
indoxacarb	ESI +	x	528	56	76	55	10	17.76
iodosulfuron-methyl	ESI +		508	141	41	35	14	13.32
iodosulfuron-methyl	ESI +	x	508	167	50	27	8	13.31
iprovalicarb	ESI +	x	321	119	60	23	6	14.85
iprovalicarb	ESI +		321	203	71	13	10	14.85
isoprocarb	ESI +		194	152	51	13	12	13.18
isoprocarb	ESI +	x	194	95	27	22	12	13.18
isoprothiolane	ESI +	x	291	189	31	31	14	15.73
isoprothiolane	ESI +		291	231	31	17	16	15.73
isoproturon	ESI +		207	165	60	19	10	13.38
isoproturon	ESI +	x	207	72	44	25	10	13.38
isoxaben	ESI +	x	333	150	41	55	10	15.76
isoxaben	ESI +		333	165	41	25	12	15.76
isoxaflutole	ESI +	x	360	251	81	21	24	14.94
isoxaflutole	ESI +		377	360	26	13	12	14.94
isoxathion	ESI +	x	314	105	46	21	12	17.16
isoxathion	ESI +		314	170	41	19	12	17.16
lenacil	ESI +	x	235	153	70	21	8	13.61
lenacil	ESI +		235	82	26	49	14	13.61
linuron	ESI +	x	249	160	80	23	8	13.98
linuron	ESI +		249	182	91	21	8	13.98
lufenuron	ESI +		511	158	55	27	8	17.39
lufenuron	ESI +	x	513	158	71	29	14	17.39
malaoxon	ESI +	x	315	127	61	19	8	13.42
malaoxon	ESI +		315	99	61	37	16	13.42
malathion	ESI +		331	285	41	11	8	15.65
malathion	ESI +	x	331	127	25	17	6	15.64
mandipropamid	ESI +		412	328	61	21	10	16.00
mandipropamid	ESI +	x	412	356	61	15	10	16.00
mepanipyrim	ESI +	x	224	106	38	37	8	15.54
mepanipyrim	ESI +		224	77	51	49	4	15.54
metalaxyl	ESI +	x	280	160	71	31	8	14.48
metalaxyl	ESI +		280	220	66	19	12	14.48
metamitron	ESI +		203	104	56	29	12	12.09
metamitron	ESI +	x	203	175	60	29	12	12.10
metazachlor	ESI +		278	134	26	27	10	14.34
metazachlor	ESI +	x	278	210	21	16	12	14.34
metconazole	ESI +		320	70	31	45	6	15.96
metconazole	ESI +	x	322	70	36	49	6	15.96
methabenzthiazuron	ESI +	x	222	150	51	51	10	13.94
methabenzthiazuron	ESI +		222	165	25	23	8	13.94
methacrifos	ESI +		241	125	26	27	6	14.34
methacrifos	ESI +	x	241	209	26	11	12	14.34

Analyte	ESI mode	Quant. ion	Precursor ion [m/z]	Product ion [m/z]	DP [V]	CE [V]	CXP [V]	RT [min]
methamidophos	ESI +		142	125	66	17	10	11.00
methamidophos	ESI +	x	142	94	51	21	8	11.00
methidathion	ESI +	x	303	145	21	13	10	15.19
methidathion	ESI +		303	85	26	27	10	15.19
methiocarb	ESI +		226	121	31	27	2	14.48
methiocarb	ESI +	x	226	169	31	15	4	14.46
methiocarb-sulfone	ESI +		275	258	21	13	14	12.33
methiocarb-sulfone	ESI +	x	275	201	21	19	16	12.33
methiocarb-sulfoxide	ESI +	x	242	107	36	59	18	11.73
methiocarb-sulfoxide	ESI +		242	185	36	19	16	11.74
metholachlor	ESI +		284	252	30	19	14	16.00
metholachlor	ESI +	x	286	254	16	19	12	16.00
methomyl	ESI +	x	163	106	11	15	2	11.63
methomyl	ESI +		163	88	45	13	6	11.64
methoprotryn	ESI +	x	272	198	46	33	12	14.43
methoprotryn	ESI +		272	240	46	27	18	14.43
methoxyfenozide	ESI +	x	369	133	41	31	10	15.43
methoxyfenozide	ESI +		369	149	50	23	8	15.43
metobromuron	ESI +	x	259	170	70	25	8	13.29
metobromuron	ESI +		261	172	51	25	12	13.28
metolcarb	ESI +	x	166	109	75	17	6	12.41
metolcarb	ESI +		166	94	26	35	18	12.42
metoxuron	ESI +	x	229	156	40	31	8	12.43
metoxuron	ESI +		229	72	21	35	6	12.43
metrafenone	ESI +		409	209	26	21	16	17.51
metrafenone	ESI +	x	409	227	26	31	18	17.51
metribuzin	ESI +		215	187	45	25	10	12.88
metribuzin	ESI +	x	215	84	31	29	6	12.88
metsulfuron-methyl	ESI +		382	167	36	21	12	12.64
metsulfuron-methyl	ESI +	x	382	199	45	27	10	12.64
molinate	ESI +	x	188	126	21	19	8	14.98
molinate	ESI +		188	83	21	25	8	14.98
monocrotophos	ESI +	x	224	193	26	11	12	11.44
monocrotophos	ESI +		224	98	46	17	12	11.44
monolinuron	ESI +		215	126	75	25	6	13.01
monolinuron	ESI +	x	215	148	56	19	8	13.01
monuron	ESI +	x	199	126	26	35	10	12.42
monuron	ESI +		199	72	45	29	4	12.43
napropamide	ESI +		272	129	45	21	6	16.25
napropamide	ESI +	x	272	171	66	29	12	16.25
neburon	ESI +	x	275	114	36	21	10	15.10
neburon	ESI +		277	88	126	25	14	15.10
nicosulfuron	ESI +		411	182	61	28	14	13.29
nicosulfuron	ESI +	x	411	213	61	23	14	13.29
novaluron	ESI +	x	493	141	66	69	12	16.82
novaluron	ESI +		493	158	66	27	18	16.82
nuarimol	ESI +		315	139	36	51	10	14.64

Analyte	ESI mode	Quant. ion	Precursor ion [m/z]	Product ion [m/z]	DP [V]	CE [V]	CXP [V]	RT [min]
nuarimol	ESI +	x	315	252	50	31	10	14.64
ofurace	ESI +		282	160	46	29	8	14.52
ofurace	ESI +	x	282	254	55	17	14	14.52
omethoate	ESI +	x	214	125	46	29	10	11.01
omethoate	ESI +		214	183	60	16	13	11.01
orbencarb	ESI +		258	100	41	19	10	16.65
orbencarb	ESI +	x	260	100	36	21	16	16.65
oxadixyl	ESI +		296	132	6	47	10	13.63
oxadixyl	ESI +	x	296	219	6	21	14	13.62
oxamyl	ESI +	x	237	72	15	21	10	11.51
oxamyl	ESI +		237	90	21	13	4	11.51
oxamyl-oxime	ESI +		163	72	26	17	4	11.27
oxamyl-oxime	ESI +	x	163	90	26	23	4	11.27
oxydemeton-methyl	ESI +		247	109	61	35	6	11.35
oxydemeton-methyl	ESI +	x	247	169	31	21	14	11.35
paclobutrazol	ESI +		294	125	21	49	6	14.31
paclobutrazol	ESI +	x	294	70	50	39	10	14.30
paraoxon-ethyl	ESI +	x	276	220	56	21	16	14.15
paraoxon-ethyl	ESI +		276	248	56	15	18	14.15
paraoxon-methyl	ESI +		248	109	51	35	10	12.95
paraoxon-methyl	ESI +	x	248	202	56	25	10	12.95
penconazole	ESI +	x	284	159	55	39	8	15.74
penconazole	ESI +		284	70	41	29	12	15.73
pencycuron	ESI +		329	125	55	33	6	16.93
pencycuron	ESI +	x	329	89	46	75	10	16.93
pendimethalin	ESI +	x	282	194	26	23	10	18.21
pendimethalin	ESI +		282	212	10	15	10	18.21
pentanochlor	ESI +	x	240	142	41	25	10	14.92
pentanochlor	ESI +		240	43	51	47	12	14.93
phenmedipham	ESI +	x	301	136	56	25	10	14.27
phenmedipham	ESI +		301	168	56	15	8	14.27
phorate	ESI +		261	47	16	47	4	16.48
phorate	ESI +	x	261	75	16	17	6	16.48
phorate-sulfone	ESI +	x	293	171	61	17	12	14.20
phorate-sulfone	ESI +		293	97	61	49	18	14.20
phorat-sulfoxide	ESI +	x	277	143	51	25	10	13.96
phorat-sulfoxide	ESI +		277	199	51	15	10	13.95
phosmet	ESI +		318	133	31	49	10	15.94
phosmet	ESI +	x	318	160	50	19	14	15.94
phosphamidon	ESI +		300	127	36	27	10	12.90
phosphamidon	ESI +	x	300	227	71	19	18	12.90
phoxim	ESI +	x	299	125	42	15	12	17.05
phoxim	ESI +		299	129	42	17	6	17.05
picoxystrobin	ESI +	x	368	145	50	27	8	16.61
picoxystrobin	ESI +		368	205	41	15	12	16.62
piperonyl butoxide	ESI +	x	356	149	26	49	12	17.68
piperonyl butoxide	ESI +		356	177	26	23	12	17.68

Analyte	ESI mode	Quant. ion	Precursor ion [m/z]	Product ion [m/z]	DP [V]	CE [V]	CXP [V]	RT [min]
pirimicarb	ESI +		239	182	16	21	12	14.00
pirimicarb	ESI +	x	239	72	30	31	10	14.01
pirimicarb-desmethyl	ESI +		225	168	16	19	8	12.70
pirimicarb-desmethyl	ESI +	x	225	72	25	25	10	12.71
pirimicarb-desmethylformamido	ESI +		253	225	16	15	10	13.92
pirimicarb-desmethylformamido	ESI +	x	253	56	31	65	4	13.92
primisulfuron-methyl	ESI +	x	469	199	71	25	10	14.24
primisulfuron-methyl	ESI +		469	254	85	25	12	14.24
prochloraz	ESI +		376	266	31	23	18	17.30
prochloraz	ESI +	x	376	308	30	17	16	17.30
promecarb	ESI +	x	208	109	25	21	6	14.29
promecarb	ESI +		208	151	56	13	14	14.29
prometon	ESI +	x	226	142	35	29	8	13.80
prometon	ESI +		226	184	41	27	16	13.81
prometryn	ESI +	x	242	158	51	35	14	14.84
prometryn	ESI +		242	200	36	29	16	14.84
propamocarb	ESI +	x	189	102	30	23	4	3.65
propamocarb	ESI +		189	74	26	33	14	3.64
propargite	ESI +		368	175	21	23	14	18.13
propargite	ESI +	x	368	231	21	15	14	18.13
propazine	ESI +	x	230	146	45	29	8	13.84
propazine	ESI +		230	188	51	25	12	13.84
propham	ESI +		180	120	11	25	12	13.09
propham	ESI +	x	180	138	30	13	8	13.09
propiconazole	ESI +	x	342	159	40	33	8	16.63
propiconazole	ESI +		344	161	36	47	14	16.64
propoxur	ESI +	x	210	111	25	19	6	12.79
propoxur	ESI +		210	168	11	11	10	12.79
propoxycarbazone	ESI +	x	416	116	25	41	6	12.03
propoxycarbazone	ESI +		416	199	11	27	12	12.03
proquinazid	ESI +	x	373	289	31	35	8	18.19
proquinazid	ESI +		373	331	31	21	10	18.19
prosulfocarb	ESI +	x	252	128	61	23	14	17.02
prosulfocarb	ESI +		252	91	55	30	12	17.01
prosulfuron	ESI +		420	141	70	27	6	13.57
prosulfuron	ESI +	x	420	167	61	25	10	13.57
pymetrozine	ESI +	x	218	105	46	31	8	11.47
pymetrozine	ESI +		218	79	51	47	12	11.47
pyraclostrobin	ESI +		388	164	50	27	10	17.58
pyraclostrobin	ESI +	x	388	194	20	19	10	17.58
pyraflufen-ethyl	ESI +	x	413	261	51	45	16	16.74
pyraflufen-ethyl	ESI +		413	339	51	29	10	16.74
pyrethrin I	ESI +		329	133	41	23	12	18.09
pyrethrin I	ESI +	x	329	161	41	13	14	18.09
pyrethrin II	ESI +	x	373	161	41	15	14	17.75
pyrethrin II	ESI +		390	161	56	19	8	17.75
pyridate	ESI +	x	379	207	20	21	10	19.07

Analyte	ESI mode	Quant. ion	Precursor ion [m/z]	Product ion [m/z]	DP [V]	CE [V]	CXP [V]	RT [min]
pyridate	ESI +		381	209	6	21	10	19.07
pyridate-metabol (6-chloro-3-phenylpyridazin-4-ol)	ESI +	x	207	104	66	31	12	12.04
pyridate-metabol (6-chloro-3-phenylpyridazin-4-ol)	ESI +		207	77	66	43	12	12.04
pyrimethanil	ESI +	x	200	107	46	33	6	14.48
pyrimethanil	ESI +		200	82	61	35	16	14.47
pyrimidifen	ESI +		378	184	46	33	16	17.90
pyrimidifen	ESI +	x	380	186	41	33	16	17.90
pyriproxyfen	ESI +		322	185	26	29	16	18.02
pyriproxyfen	ESI +	x	322	96	30	21	4	18.01
quizalofop-ethyl	ESI +	x	373	299	85	25	16	17.93
quizalofop-ethyl	ESI +		375	301	61	29	10	17.93
rabenzazole	ESI +	x	213	157	62	42	10	13.51
rabenzazole	ESI +		213	172	62	31	10	13.51
rimsulfuron	ESI +	x	432	182	50	29	8	13.70
rimsulfuron	ESI +		432	325	50	21	15	13.71
rotenone	ESI +	x	395	192	71	31	18	18.24
rotenone	ESI +		395	213	51	33	18	18.24
sebuthylazine	ESI +		230	132	61	31	8	13.81
sebuthylazine	ESI +	x	230	174	35	25	8	13.81
sethoxydim	ESI +	x	328	178	26	29	6	16.92
sethoxydim	ESI +		328	282	30	17	16	16.92
silafluofen	ESI +	x	426	168	11	47	16	19.72
silafluofen	ESI +	x	426	287	11	15	22	19.72
simazine	ESI +		202	124	26	25	12	12.61
simazine	ESI +	x	202	132	46	29	12	12.61
simazine-desethyl	ESI +		174	146	41	30	10	11.56
simazine-desethyl	ESI +	x	174	68	41	41	6	11.55
simeconazole	ESI +	x	294	135	31	31	10	14.55
simeconazole	ESI +		294	70	31	35	12	14.55
spinosyn A	ESI +	x	733	142	65	37	8	18.80
spinosyn A	ESI +		733	98	66	75	4	18.80
spinosyn D	ESI +	x	747	142	80	39	8	19.16
spinosyn D	ESI +		747	98	76	79	6	19.16
spirodiclofen	ESI +		411	313	61	17	10	18.38
spirodiclofen	ESI +	x	411	71	36	30	10	18.38
spiromesifen	ESI +	x	273	187	66	25	12	18.14
spiromesifen	ESI +		273	255	66	21	8	18.14
spirotetramat	ESI +		374	302	51	25	10	16.21
spirotetramat	ESI +	x	374	330	56	23	10	16.21
spiroxamine	ESI +		298	100	41	41	12	15.93
spiroxamine	ESI +	x	298	144	55	27	6	15.93
tebuconazole	ESI +		308	125	21	47	10	15.63
tebuconazole	ESI +	x	308	70	46	37	6	15.62
tebufenozide	ESI +		353	133	50	23	6	15.86
tebufenozide	ESI +	x	353	297	16	13	20	15.85
tebufenpyrad	ESI +	x	334	117	51	47	6	17.13

Analyte	ESI mode	Quant. ion	Precursor ion [m/z]	Product ion [m/z]	DP [V]	CE [V]	CXP [V]	RT [min]
tebufenpyrad	ESI +		334	145	66	39	10	17.13
teflubenzuron	ESI +	x	381	141	51	55	12	17.28
teflubenzuron	ESI +		381	158	41	25	12	17.28
tetraethyl diphosphate	ESI +	x	291	179	66	31	12	12.69
tetraethyl diphosphate	ESI +		291	99	66	55	18	12.69
terbacil	ESI +		161	144	49	25	10	12.70
terbacil	ESI +	x	161	88	49	33	8	12.71
terbufos	ESI +	x	289	103	51	13	6	17.18
terbufos	ESI +		289	57	51	31	4	17.18
terbufos-sulfone	ESI +	x	338	115	16	45	8	15.18
terbufos-sulfone	ESI +		338	171	16	23	14	15.17
terbufos-sulfoxide	ESI +		305	131	21	39	10	15.01
terbufos-sulfoxide	ESI +	x	305	187	21	17	16	15.01
terbuthylazine	ESI +		230	132	61	31	8	14.10
terbuthylazine	ESI +	x	230	174	35	23	8	14.10
terbuthylazine-desethyl	ESI +	x	202	146	36	23	10	12.54
terbuthylazine-desethyl	ESI +		204	148	41	23	10	12.54
terbutryn	ESI +	x	242	186	35	25	10	15.08
terbutryn	ESI +		242	96	41	43	16	15.08
tetraconazole	ESI +	x	372	159	56	47	10	15.23
tetraconazole	ESI +		372	70	36	47	10	15.23
thiabendazole	ESI +		202	131	61	43	10	12.51
thiabendazole	ESI +	x	202	175	75	37	14	12.51
thiabendazole-5-hydroxy	ESI +	x	218	191	71	35	10	11.46
thiabendazole-5-hydroxy	ESI +		218	147	66	43	10	11.46
thiacloprid	ESI +	x	253	126	95	29	6	13.05
thiacloprid	ESI +		253	90	41	49	8	13.05
thiametoxam	ESI +		292	181	36	31	10	11.72
thiametoxam	ESI +	x	292	211	46	19	18	11.73
thiazafluron	ESI +	x	241	184	16	25	16	12.50
thiazafluron	ESI +		241	91	46	39	16	12.50
thifensulfuron-methyl	ESI +	x	388	167	50	21	8	12.75
thifensulfuron-methyl	ESI +		388	205	51	37	20	12.76
thiodicarb	ESI +		355	108	26	21	12	15.35
thiodicarb	ESI +	x	355	88	40	21	14	15.36
thiofanox	ESI +	x	219	57	30	17	8	13.33
thiofanox	ESI +		219	61	16	15	12	13.33
thiofanox-sulfone	ESI +	x	251	57	56	23	10	11.84
thiofanox-sulfone	ESI +		251	76	56	11	14	11.85
thiofanox-sulfoxide	ESI +	x	235	104	56	13	18	11.68
thiofanox-sulfoxide	ESI +		235	57	56	27	10	11.69
thiometon	ESI +		247	61	26	49	8	14.06
thiometon	ESI +	x	247	89	26	17	4	14.06
thionazin	ESI +		249	113	31	29	10	14.13
thionazin	ESI +	x	249	97	31	37	8	14.13
thiophanate-ethyl	ESI +		371	151	46	25	8	14.72
thiophanate-ethyl	ESI +	x	371	325	46	21	8	14.71

Analyte	ESI mode	Quant. ion	Precursor ion [m/z]	Product ion [m/z]	DP [V]	CE [V]	CXP [V]	RT [min]
thiophanate-methyl	ESI +	x	343	151	46	25	8	13.32
thiophanate-methyl	ESI +		343	311	36	15	10	13.33
tiocarbazil	ESI +		280	100	36	21	8	17.80
tiocarbazil	ESI +	x	280	91	36	45	6	17.80
triadimefon	ESI +	x	294	197	41	23	16	15.05
triadimefon	ESI +		294	225	36	19	10	15.05
triadimenol	ESI +	x	296	70	16	21	4	14.34
triadimenol	ESI +		298	70	16	25	8	14.43
triamiphos	ESI +	x	295	44	86	67	4	14.11
triamiphos	ESI +		295	92	86	53	8	14.11
triasulfuron	ESI +		402	141	41	29	10	13.93
triasulfuron	ESI +	x	402	167	46	25	12	13.92
triazamate	ESI +	x	315	226	36	17	14	15.75
triazamate	ESI +		315	184	36	43	16	15.75
triazophos	ESI +	x	314	119	46	47	6	16.10
triazophos	ESI +		314	162	46	27	14	16.10
tribenuron-methyl	ESI +	x	396	155	65	21	8	16.08
tribenuron-methyl	ESI +		396	181	51	29	16	16.08
trichlorfon	ESI +		257	221	66	15	6	11.67
trichlorfon	ESI +	x	259	109	71	25	8	11.66
tricyclazole	ESI +		190	136	71	37	10	13.75
tricyclazole	ESI +	x	190	163	71	31	14	13.75
tridemorph	ESI +		298	130	70	35	6	17.59
tridemorph	ESI +	x	298	98	56	41	8	17.59
trietazine	ESI +		230	132	26	29	12	14.96
trietazine	ESI +	x	230	99	26	33	12	14.96
trifloxystrobin	ESI +		409	186	30	25	4	17.46
trifloxystrobin	ESI +	x	409	206	30	21	10	17.46
trifloxysulfuron	ESI +	x	438	156	41	39	10	14.43
trifloxysulfuron	ESI +		438	182	41	27	12	14.43
triflumizole	ESI +	x	346	278	20	17	16	16.92
triflumizole	ESI +		346	73	6	23	16	16.91
triflumizole-metabole FM-6-1	ESI +		295	215	126	33	16	13.78
triflumizole-metabole FM-6-1	ESI +	x	295	278	126	21	20	13.78
triflumuron	ESI +	x	359	139	51	47	12	16.17
triflumuron	ESI +		359	156	46	25	12	16.17
triflusulfuron-methyl	ESI +		493	238	40	29	20	15.93
triflusulfuron-methyl	ESI +	x	493	264	60	29	14	15.93
triforine	ESI +		435	390	26	27	12	13.70
triforine	ESI +	x	437	392	51	15	10	13.70
triticonazole	ESI +		318	70	50	33	4	15.20
triticonazole	ESI +	x	320	70	36	33	10	15.21
uniconazole	ESI +		292	125	46	43	10	14.96
uniconazole	ESI +	x	292	70	45	37	10	14.96
vamidothion	ESI +		288	118	36	31	6	11.94
vamidothion	ESI +	x	288	146	30	17	8	11.95
vamidothion-sulfone	ESI +	x	320	178	61	21	12	11.50

Analyte	ESI mode	Quant. ion	Precursor ion [m/z]	Product ion [m/z]	DP [V]	CE [V]	CXP [V]	RT [min]
vamidothion-sulfone	ESI +		320	58	61	65	12	11.50
vamidothion-sulfoxide	ESI +		304	169	46	23	12	10.93
vamidothion-sulfoxide	ESI +	x	304	201	46	17	14	10.93
zoxamide	ESI +	x	336	187	41	37	12	15.84
zoxamide	ESI +		338	189	36	33	12	15.84
diuron-d6	ESI -		237	186	-40	-26	-15	13.27
alternariol	ESI -		257	212	-165	-40	-17	13.65
alternariol	ESI -	x	257	215	-165	-34	-29	13.64
alternariol monomethyl ether	ESI -		271	256	-5	-32	-25	16.30
alternariol monomethyl ether	ESI -	x	271	255	-50	-44	-21	16.30
citrinin	ESI -	x	249	175	-190	-40	-13	12.13
citrinin	ESI -		249	205	-190	-24	-19	12.14
3-acetyldeoxynivalenol	ESI -		383	307	-40	-13	-10	12.02
3-acetyldeoxynivalenol	ESI -		383	247	-40	-18	-8	12.02
deoxynivalenol	ESI -		341	265	-40	-16	-9	11.05
deoxynivalenol-3-glucoside	ESI -	x	503	427	-75	-28	-13	4.03
nivalenol	ESI -	x	357	281	-40	-18	-10	2.47
nivalenol	ESI -		357	203	-40	-24	-7	2.47
ochratoxin A	ESI -		402	211	-70	-40	-7	15.17
ochratoxin A	ESI -	x	402	167	-70	-50	-8	15.17
zearalenone	ESI -	x	317	131	-10	-42	-7	16.04
zearalenone	ESI -		317	175	-10	-32	-17	16.04
zearalenone-14-sulfate	ESI -		397	175	-80	-48	-19	13.38
zearalenone-14-sulfate	ESI -		397	317	-80	-30	-35	13.38
zearalenone-14,16-disulfate	ESI -	x	477	397	-65	-20	-13	11.43
zearalenone-14,16-disulfate	ESI -		238	80	-75	-22	-13	11.42
α-zearalenol	ESI -		319	160	-150	-42	-19	14.87
α-zearalenol	ESI -	x	319	174	-150	-36	-15	14.86
α-zearalenol-14-sulfate	ESI -		399	174	-100	-52	-17	12.66
α-zearalenol-14-sulfate	ESI -	x	399	319	-100	-34	-37	12.66
β-zearalenol	ESI -		319	160	-5	-40	-15	14.10
β-zearalenol	ESI -	x	319	174	-5	-38	-23	14.10
β-zearalenol-14-sulfate	ESI -		399	275	-85	-44	-29	12.00
β-zearalenol-14-sulfate	ESI -	x	399	319	-85	-34	-9	12.00

Table S4 Spiking Levels of Validation of Mycotoxins, Plant Growth Regulators, Tropane Alkaloids, and Pesticides.

Level	Replicates	Mycotoxins (x-times estimated LOQ)	Deoxynivalenol-3-glucoside [mg/kg]	Plant growth regulators [mg/kg]	Tropane alkaloids [mg/kg]	Pesticides [mg/kg]	Aminopyralid, dodine [mg/kg]
1	6	1	0.05	0.005	0.005	0.005	0.05
2	6	1.5	0.0075	0.0075	0.0075	0.0075	0.0075
3	6	2	0.1	0.01	0.01	0.01	0.1
4	1	4	0.2	0.02	0.02	0.02	0.2
5	1	10	0.5	0.05	0.05	0.05	0.5
6	1	15	0.75	0.075	0.075	0.075	0.75
7	6	20	1	0.1	0.1	0.1	1
8	6	40	-	0.2	0.2	0.2	2
9	1	70	-	0.35	0.35	0.35	3.5
10	1	100	-	0.5	0.5	0.5	5

Table S5 Evaluated Levels, Calibration Range, R^2, and Measurement Uncertainty (MU, k = 2) for Mycotoxins, Plant Growth Regulators, and Tropane Alkaloids in Wheat.

Analyte	Evaluated levels	Calibration range [µg/kg]	R^2	MU [%][a]
aflatoxin B1	7	1 - 40	0.9997	20
aflatoxin B2	7	1 - 40	0.9993	41
aflatoxin G1	7	1 - 40	0.9971	30
aflatoxin G2	7	1 - 40	0.9999	35
altenuene	7	20 - 800	0.9999	26
alternariol	7	1 - 70	0.9975	31
alternariol monomethyl ether	7	1 - 70	0.9998	20
citrinin	7	50 - 2000	0.9943	38
deoxynivalenol	7	50 - 2000	0.9998	28
deoxynivalenol-3-glucoside	7	50 - 1000	0.9996	19
15-acetyldeoxynivalenol	7	50 - 2000	0.9994	28
3-acetyldeoxynivalenol	7	50 - 2000	0.9992	39
diacetoxyscirpenol	7	5 - 200	0.9994	19
fumonisin B1	7	10 - 400	0.9996	297
hydrolyzed fumonisin B1	7	100 - 4000	0.9967	24
fumonisin B2	7	10 - 400	0.9961	138
hydrolyzed fumonisin B2	7	100 - 4000	0.9995	15
fumonisin B3	7	10 - 400	0.9969	139
fusarenon X	7	300 - 8000	0.9992	19
HT-2 toxin	7	50 - 2000	0.9999	31
neosolaniol	7	5 - 200	0.9988	39
nivalenol	7	200 - 8000	0.9996	19
ochratoxin A	7	3 - 120	0.9998	28
sterigmatocystin	7	1 - 40	0.9999	23
T-2 toxin	7	10 - 400	0.9998	27
tentoxin	7	5 - 200	0.9978	36
zearalenone	7	10 - 400	0.9988	23
zearalenone-14-glucoside	7	50 - 2000	0.9997	28
zearalenone-14-sulfate	7	50 - 2000	0.9999	26
zearalenone-14,16-disulfate	7	100 - 4000	0.9996	38
α-zearalenol	7	10 - 400	0.9992	21
α-zearalenol-14-glucoside	7	50 - 2000	0.9983	44
α-zearalenol-14-sulfate	7	10 - 400	0.9942	26
β-zearalenol	7	10 - 400	0.9975	25
β-zearalenol-14-glucoside	7	50 - 2000	0.9996	47
β-zearalenol-14-sulfate	7	10 - 400	0.9953	32
zearalanone	7	50 - 2000	0.9921	21
zearalanone-14-glucoside	7	10 - 400	0.9998	24
α-zearalanol	7	100 - 4000	0.9994	22
β-zearalanol	7	100 - 4000	0.9983	39
chlormequat	7	5 - 350	0.9998	10
mepiquat	7	5 - 350	0.9998	12
atropine	7	5 - 200	0.9991	18
scopolamine	7	5 - 200	0.9992	14

[a]Evaluated at spiking level of LOQ.

Table S6 Limits of Quantification (LOQ), Evaluated Levels, Calibration Range, R^2, and Measurement Uncertainty (MU, k = 2) for Pesticides in Wheat.

Analyte	LOQ [µg/kg]	Evaluated levels	Calibration range [µg/kg]	R^2	MU [%][a]
3,4,5-trimethacarb	5	6	5 - 200	0.9998	35
acephate	10	6	10 - 200	0.9962	18
acetamiprid	5	6	5 - 200	0.9964	17
acetochlor	10	6	10 - 200	0.9930	32
alachlor	100	4	100 - 500	0.9962	16
aldicarb	5	6	5 - 200	0.9993	23
aldicarb-sulfoxide	5	6	5 - 200	0.9999	16
aldoxycarb	5	6	5 - 200	0.9993	25
ametoctradin	5	6	5 - 200	0.9993	36
ametryn	5	6	5 - 100	0.9992	15
amidosulfuron	5	6	5 - 75	0.9999	23
aminocarb	5	6	5 - 200	0.9992	29
aminopyralid	50	6	50 - 1000	0.9999	16
amitraz	100	4	100 - 500	0.9995	86
amitraz-amide	10	6	10 - 200	0.9991	22
amitraz-amidin	5	6	5 - 200	0.9969	22
ancymidol	10	6	10 - 200	0.9892	26
atrazine	5	6	5 - 200	0.9997	15
atrazine-desethyl-desisopropyl	5	6	5 - 100	0.9991	18
avermectin B1a[b]	100	4	100 - 500	0.9989	42
avermectin B1b[b]	n.a.	n.a.	n.a.	n.a.	n.a.
azaconazole	5	6	5 - 200	0.9985	28
azamethiphos	5	6	5 - 100	0.9971	29
aziprotryne	5	6	5 - 200	0.9996	40
azoxystrobin	5	6	5 - 100	0.9993	12
benalaxyl	5	6	5 - 100	0.9996	13
bendiocarb	10	6	10 - 200	0.9906	86
benfuracarb	10	6	10 - 350	0.9971	76
benodanil	5	6	5 - 100	0.9999	24
benomyl	n.a.	n.a.	n.a.	n.a.	n.a.
bensulfuron-methyl	5	6	5 - 200	0.9899	23
benthiavalicarb-isopropyl	5	6	5 - 200	0.9989	27
bitertanol	10	6	10 - 350	0.9999	34
boscalid	5	6	5 - 200	0.9999	24
bromacil	5	6	5 - 200	0.9993	22
bromuconazole	10	6	10 - 350	0.9998	18
bupirimate	5	6	5 - 200	0.9992	26
buprofezin	5	6	5 - 200	0.9999	22
butachlor	10	6	10 - 350	0.9995	35
butocarboxim	5	6	5 - 200	0.9999	22
butocarboxim-sulfoxide	5	6	5 - 200	0.9998	16
butoxycarboxim	5	6	5 - 200	0.9999	21

Analyte	LOQ [µg/kg]	Evaluated levels	Calibration range [µg/kg]	R^2	MU [%][a]
buturon	5	6	5 - 200	0.9985	34
cadusafos	5	6	5 - 200	0.9987	23
carbaryl	5	6	5 - 200	0.9961	202
carbendazim	5	6	5 - 100	0.9936	12
carbofuran	5	6	5 - 100	0.9947	64
carbofuran-3-hydroxy	5	6	5 - 200	0.9996	188
carbosulfan	5	6	5 - 75	0.9856	100
carboxin	5	6	5 - 100	0.9978	15
chlorantraniliprole	5	6	5 - 200	0.9994	26
chlorbromuron	5	6	5 - 200	0.9985	18
chlorfluazuron	5	6	5 - 200	0.9999	29
chloridazon	5	6	5 - 200	0.9961	33
chloroxuron	5	6	5 - 200	0.9996	30
chlorpropham	100	4	100 - 500	0.9991	31
chlorsulfuron	5	6	5 - 200	0.9986	28
chlortoluron	5	6	5 - 200	0.9968	13
cinidon-ethyl	10	6	10 - 350	0.9999	23
cinosulfuron	5	6	5 - 200	0.9946	20
clethodim	5	6	5 - 200	0.9997	28
clodinafop-propargyl	5	6	5 - 100	0.9971	16
clofentezine	5	6	5 - 200	0.9999	28
clomazone	5	6	5 - 200	0.9964	30
clothianidin	10	6	10 - 350	0.9918	24
cyanazine	5	6	5 - 200	0.9999	38
cyazofamid	10	6	10 - 350	0.9943	21
cymoxanil	100	4	100 - 500	0.9967	61
cyproconazole	5	6	5 - 200	0.9995	17
cyprodinil	10	6	10 - 350	0.9997	27
cyprofuram	5	6	5 - 200	0.9975	20
cyromazine	5	6	5 - 200	0.9996	46
daminozide	100	4	100 - 500	0.9941	19
demeton	5	6	5 - 200	0.9997	23
demeton-S-methyl	5	6	5 - 200	0.9991	21
demeton-S-methyl-sulfone	5	6	5 - 200	0.9986	17
desmedipham	n.a.	n.a.	n.a.	n.a.	n.a.
desmetryn	5	6	5 - 100	0.9975	21
diazinon	5	6	5 - 200	0.9992	25
dichlorvos	100	4	100 - 500	0.9937	21
diclobutrazol	100	4	100 - 500	0.9915	15
diethofencarb	5	6	5 - 200	0.9994	25
diethyltoluamide	5	6	5 - 100	0.9946	24
difenoconazole	5	6	5 - 200	0.9998	19
difenoxuron	5	6	5 - 200	0.9997	20
diflubenzuron	5	6	5 - 200	0.9992	22
diflufenican	5	6	5 - 200	0.9993	26
dimefox	100	4	100 - 500	0.9949	16

Analyte	LOQ [µg/kg]	Evaluated levels	Calibration range [µg/kg]	R^2	MU [%][a]
dimefuron	5	6	5 - 200	0.9996	20
dimethenamid	5	6	5 - 200	0.9997	35
dimethoate	5	6	5 - 200	0.9986	23
dimethomorph	5	6	5 - 200	0.9993	29
dimetilan	5	6	5 - 200	0.9921	13
dimoxystrobin	5	6	5 - 200	0.9971	25
dinotefuran	5	6	5 - 200	0.9996	19
disulfoton	5	6	5 - 200	0.9999	48
disulfoton-sulfone	5	6	5 - 200	0.9991	30
disulfoton-sulfoxide	5	6	5 - 200	0.9983	19
diuron	5	6	5 - 200	0.9980	19
dodine	n.a.	n.a.	n.a.	n.a.	n.a.
emamectin B1a[c]	5	6	5 - 200	0.9994	34
emamectin B1b[c]	5	6	5 - 200	0.9989	89
epoxiconazole	5	6	5 - 200	0.9997	29
ethiofencarb	5	6	5 - 200	0.9906	250
ethiofencarb-sulfone	100	4	100 - 500	0.9926	238
ethiofencarb-sulfoxide	100	4	100 - 500	0.9975	230
ethiprole	5	6	5 - 200	0.9994	32
ethofumesate	5	6	5 - 200	0.9990	40
ethofumesate-2-keto	100	4	100 - 500	0.9940	35
ethoprofos	5	6	5 - 200	0.9999	25
etofenprox	5	6	5 - 200	0.9996	31
etoxazole	5	6	5 - 200	0.9998	23
famoxadon	5	6	5 - 200	0.9998	24
fenamidone	5	6	5 - 200	0.9986	14
fenamiphos	5	6	5 - 200	0.9975	35
fenamiphos-sulfone	5	6	5 - 200	0.9989	41
fenamiphos-sulfoxide	5	6	5 - 200	0.9996	29
fenarimol	10	6	10 - 350	0.9998	36
fenazaquin	5	6	5 - 200	0.9999	8
fenbuconazole	5	6	5 - 200	0.9998	38
fenhexamid	10	6	10 - 350	0.9994	47
fenobucarb	5	6	5 - 200	0.9999	34
fenoxaprop-P-ethyl	10	6	10 - 200	0.9878	27
fenoxycarb	5	6	5 - 200	0.9979	23
fenpiclonil	5	6	5 - 200	0.9996	42
fenpropidin	5	6	5 - 200	0.9991	16
fenpropimorph	5	6	5 - 200	0.9998	21
fenpyroximate	5	6	5 - 200	0.9956	15
fensulfothion	5	6	5 - 200	0.9961	25
fensulfothion-PO-sulfone	5	6	5 - 200	0.9935	22
fensulfothion-PO-sulfoxide	5	6	5 - 200	0.9997	42
fensulfothion-PS-sulfone	5	6	5 - 200	0.9998	23
fenthion	10	6	10 - 350	0.9997	18

Analyte	LOQ [µg/kg]	Evaluated levels	Calibration range [µg/kg]	R^2	MU [%][a]
fenthion-oxon	5	6	5 - 200	0.9995	21
fenthion-PO-sulfone	5	6	5 - 200	0.9971	23
fenthion-PO-sulfoxide	5	6	5 - 200	0.9997	26
fenthion-PS-sulfone	10	6	10 - 350	0.9992	34
fenthion-PS-sulfoxide	5	6	5 - 200	0.9969	27
fenuron	100	4	100 - 500	0.9811	14
flazasulfuron	5	6	5 - 200	0.9937	23
flonicamid	5	6	5 - 200	0.9997	41
florasulam	5	6	5 - 200	0.9992	28
fluazifop-P-butyl	5	6	5 - 200	0.9990	38
fluazuron	5	6	5 - 200	0.9996	41
flucycloxuron	5	6	5 - 200	0.9991	13
fludioxonil	10	6	10 - 200	0.9930	26
flufenacet	5	6	5 - 200	0.9956	29
flufenoxuron	5	6	5 - 200	0.9999	32
fluometuron	10	6	10 - 350	0.9929	20
fluopicolide	5	6	5 - 200	0.9997	26
flurochloridone	5	6	5 - 200	0.9994	16
flurprimidol	5	6	5 - 200	0.9996	37
flusilazole	5	6	5 - 200	0.9996	18
flutriafol	100	4	100 - 500	0.9808	11
formetanate	5	6	5 - 200	0.9995	77
fosthiazate	5	6	5 - 200	0.9993	18
fuberidazole	5	6	5 - 200	0.9946	20
furathiocarb	5	6	5 - 200	0.9990	72
halofenozide	5	6	5 - 200	0.9969	22
haloxyfop	5	6	5 - 200	0.9999	37
haloxyfop-2-ethoxyethyl	5	6	5 - 200	0.9987	32
haloxyfop-methyl	5	6	5 - 100	0.9967	10
hexaconazole	5	6	5 - 200	0.9995	23
hexaflumuron	100	6	100 - 500	0.9959	17
hexazinone	5	6	5 - 200	0.9982	21
hexythiazox	5	6	5 - 200	0.9996	21
imazalil	5	6	5 - 200	0.9986	34
imibenconazole	5	6	5 - 200	0.9993	23
imidacloprid	5	6	5 - 200	0.9994	40
indoxacarb	5	6	5 - 200	0.9999	32
iodosulfuron-methyl	10	6	10 - 350	0.9948	23
iprovalicarb	5	6	5 - 200	0.9997	20
isoprocarb	10	6	10 - 350	0.9988	44
isoprothiolane	5	6	5 - 100	0.9987	35
isoproturon	10	6	10 - 200	0.9889	28
isoxaben	5	6	5 - 100	0.9986	25
isoxaflutole	n.a.	n.a.	n.a.	n.a.	n.a.
isoxathion	5	6	5 - 200	0.9966	13
lenacil	5	6	5 - 200	0.9995	25

Analyte	LOQ [µg/kg]	Evaluated levels	Calibration range [µg/kg]	R^2	MU [%][a]
linuron	5	6	5 - 200	0.9997	21
lufenuron	5	6	5 - 200	0.9999	49
malaoxon	5	6	5 - 200	0.9948	30
malathion	5	6	5 - 200	0.9997	29
mandipropamid	5	6	5 - 200	0.9995	32
mepanipyrim	100	6	100 - 500	0.9970	24
metalaxyl	5	6	5 - 200	0.9992	20
metamitron	5	6	5 - 200	0.9992	35
metazachlor	5	6	5 - 200	0.9995	25
metconazole	5	6	5 - 200	0.9991	37
methabenzthiazuron	5	6	5 - 200	0.9991	25
methacrifos	100	6	100 - 500	0.9995	24
methamidophos	10	6	10 - 350	0.9999	15
methidathion	5	6	5 - 200	0.9997	29
methiocarb	10	6	10 - 200	0.9868	229
methiocarb-sulfone	100	6	100 - 500	0.9867	222
methiocarb-sulfoxide	100	6	100 - 500	0.9963	245
metholachlor	5	6	5 - 200	0.9995	35
methomyl	5	6	5 - 200	0.9983	32
methoprotryn	5	6	5 - 200	0.9962	22
methoxyfenozide	5	6	5 - 200	0.9997	27
metobromuron	5	6	5 - 200	0.9994	22
metolcarb	100	6	100 - 500	0.9951	40
metoxuron	5	6	5 - 200	0.9996	26
metrafenone	5	6	5 - 200	0.9999	30
metribuzin	5	6	5 - 200	0.9996	22
metsulfuron-methyl	5	6	5 - 200	0.9972	50
molinate	100	6	100 - 500	0.9984	23
monocrotophos	5	6	5 - 200	0.9998	35
monolinuron	5	6	5 - 200	0.9999	21
monuron	5	6	5 - 200	0.9996	46
napropamide	5	6	5 - 200	0.9995	41
neburon	5	6	5 - 200	0.9995	27
nicosulfuron	5	6	5 - 200	0.9982	26
novaluron	5	6	5 - 200	0.9998	47
nuarimol	10	6	10 - 350	0.9999	31
ofurace	5	6	5 - 200	0.9981	19
omethoate	5	6	5 - 200	0.9999	18
orbencarb	5	6	5 - 200	0.9999	39
oxadixyl	5	6	5 - 200	0.9999	17
oxamyl	5	6	5 - 200	0.9995	15
oxamyl-oxime	5	6	5 - 200	0.9997	18
oxydemeton-methyl	5	6	5 - 200	0.9995	26
paclobutrazol	10	6	10 - 200	0.9852	18
paraoxon-ethyl	5	6	5 - 200	0.9944	28
paraoxon-methyl	10	6	10 - 350	0.9982	34
penconazole	5	6	5 - 200	0.9981	23

Analyte	LOQ [µg/kg]	Evaluated levels	Calibration range [µg/kg]	R^2	MU [%][a]
pencycuron	5	6	5 - 200	0.9993	24
pendimethalin	100	6	100 - 500	0.9987	18
pentanochlor	10	6	10 - 350	0.9914	19
phenmedipham	n.a.	n.a.	n.a.	n.a.	n.a.
phorate	100	6	100 - 500	0.9999	18
phorate-sulfone	5	6	5 - 200	0.9995	34
phorate-sulfoxide	5	6	5 - 200	0.9991	31
phosmet	10	6	10 - 350	0.9932	24
phosphamidon	5	6	5 - 200	0.9999	34
phoxim	5	6	5 - 200	0.9999	30
picoxystrobin	5	6	5 - 200	0.9932	19
piperonyl butoxide	5	6	5 - 500	0.9995	37
pirimicarb	5	6	5 - 200	0.9908	14
pirimicarb-desmethyl	5	6	5 - 200	0.9913	22
pirimicarb-desmethylformamido	100	6	100 - 500	0.9860	17
primisulfuron-Methyl	5	6	5 - 200	0.9996	36
prochloraz	5	6	5 - 200	0.9992	23
promecarb	5	6	5 - 200	0.9999	37
prometon	5	6	5 - 200	0.9980	28
prometryn	5	6	5 - 200	0.9926	20
propamocarb	5	6	5 - 200	0.9994	34
propargite	5	6	5 - 200	0.9999	8
propazine	5	6	5 - 200	0.9948	27
propham	100	6	100 - 500	0.9960	18
propiconazole	5	6	5 - 200	0.9997	28
propoxur	5	6	5 - 200	0.9990	78
propoxycarbazone	10	6	10 - 350	0.9989	47
proquinazid	5	6	5 - 200	0.9989	16
prosulfocarb	5	6	5 - 200	0.9999	20
prosulfuron	5	6	5 - 200	0.9998	22
pymetrozine	100	6	100 - 500	0.9621	75
pyraclostrobin	5	6	5 - 200	0.9998	13
pyraflufen-ethyl	5	6	5 - 200	0.9999	29
pyrethrin I	10	6	10 - 350	0.9996	18
pyrethrin II	10	6	10 - 350	0.9918	48
pyridate	n.a.	n.a.	n.a.	n.a.	n.a.
pyridate-metabol (6-chloro-3-phenylpyridazin-4-ol)	5	6	5 - 200	0.9966	27
pyrimethanil	5	6	5 - 200	0.9999	28
pyrimidifen	5	6	5 - 200	0.9995	25
pyriproxyfen	5	6	5 - 200	0.9924	19
quizalofop-ethyl	5	6	5 - 200	0.9982	15
rabenzazole	5	6	5 - 200	0.9997	34
rimsulfuron	5	6	5 - 200	0.9998	35
rotenone	5	6	5 - 200	0.9999	16
sebuthylazine	5	6	5 - 200	0.9939	29

Analyte	LOQ [µg/kg]	Evaluated levels	Calibration range [µg/kg]	R^2	MU [%][a]
sethoxydim	5	6	5 - 200	0.9996	42
silafluofen	5	6	5 - 200	0.9999	12
simazine	5	6	5 - 200	0.9993	39
simazine-desethyl	100	4	100 - 500	0.9805	15
simeconazol	100	4	100 - 500	0.9945	16
spinosyn A[d]	100	4	100 - 500	0.9778	11
spinosyn D[d]	100	4	100 - 500	0.9999	11
spirodiclofen	5	6	5 - 200	0.9998	103
spiromesifen	10	6	10 - 350	0.9994	27
spirotetramat	5	6	5 - 200	0.9965	23
spiroxamine	5	6	5 - 200	0.9976	28
tebuconazole	10	6	10 - 350	0.9990	23
tebufenozide	5	6	5 - 200	0.9992	26
tebufenpyrad	5	6	5 - 200	0.9997	37
teflubenzuron	10	6	10 - 350	0.9995	31
tetraethyl diphosphate	5	6	5 - 200	0.9946	23
terbacil	10	6	10 - 350	0.9982	34
terbufos	10	6	10 - 350	0.9995	26
terbufos-sulfone	5	6	5 - 200	0.9999	37
terbufos-sulfoxide	5	6	5 - 200	0.9976	21
terbuthylazine	5	6	5 - 200	0.9927	28
terbuthylazine-desethyl	5	6	5 - 200	0.9922	17
terbutryn	5	6	5 - 100	0.9981	13
tetraconazole	5	6	5 - 200	0.9999	30
thiabendazole	5	6	5 - 200	0.9960	26
thiabendazole-5-hydroxy	5	6	5 - 200	0.9967	18
thiacloprid	5	6	5 - 200	0.9912	13
thiametoxam	5	6	5 - 200	0.9990	20
thiazafluron	100	4	100 - 500	0.9855	10
thifensulfuron-methyl	5	6	5 - 200	0.9971	26
thiodicarb	100	4	100 - 500	0.9847	29
thiofanox	100	4	100 - 500	0.9934	42
thiofanox-sulfone	10	6	10 - 350	0.9999	33
thiofanox-sulfoxide	100	4	100 - 500	0.9993	16
thiometon	100	4	100 - 500	0.9879	28
thionazin	5	6	5 - 200	0.9997	24
thiophanate-ethyl	n.a.	n.a.	n.a.	n.a.	n.a.
thiophanate-methyl	n.a.	n.a.	n.a.	n.a.	n.a.
tiocarbazil	100	4	100 - 500	0.9988	17
triadimefon	5	6	5 - 200	0.9998	21
triadimenol	5	6	5 - 200	0.9999	18
triamiphos	5	6	5 - 200	0.9992	31
triasulfuron	5	6	5 - 200	0.9962	21
triazamate	10	6	10 - 350	0.9927	31
triazophos	5	6	5 - 200	0.9981	32
tribenuron-methyl	5	6	5 - 200	0.9976	31

Analyte	LOQ [µg/kg]	Evaluated levels	Calibration range [µg/kg]	R^2	MU [%][a]
trichlorfon	n.a.	n.a.	n.a.	n.a.	n.a.
tricyclazole	5	6	5 - 200	0.9920	20
tridemorph	100	4	100 - 500	0.9989	17
trietazine	5	6	5 - 200	0.9991	19
trifloxystrobin	5	6	5 - 200	0.9998	15
trifloxysulfuron	5	6	5 - 200	0.9999	23
triflumizole	5	6	5 - 200	0.9959	19
triflumizole-metabole FM-6-1	5	6	5 - 200	0.9996	28
triflumuron	5	6	5 - 200	0.9999	25
triflusulfuron-methyl	5	6	5 - 200	0.9952	25
triforine	5	6	5 - 200	0.9996	31
triticonazole	5	6	5 - 200	0.9996	40
uniconazole	100	4	100 - 500	0.9964	17
vamidothion	5	6	5 - 100	0.9963	25
vamidothion-sulfone	5	6	5 - 200	0.9948	31
vamidothion-sulfoxide	5	6	5 - 200	0.9999	8
zoxamide	5	6	5 - 200	0.9968	26

[a]Evaluated at spiking level of LOQ.

[b]Mixture of B1a and B1b. Isomeric ratio: B1a 93.3%, B1b 6.6%.

[c]Mixture of B1a and B1b. Isomeric ratio unknown.

[d]Mixture of A and B. Isomeric ratio: A 84%, B 16%.

Table S7 Recoveries (R_E, n = 6), Repeatabilities (RSDr), and Within-Laboratory Reproducibilities (RSDR) for Pesticides at 5 Spiking Levels in Wheat.

Analyte	Spiking level 1			Spiking level 2			Spiking level 3			Spiking level 7			Spiking level 8		
	R_E (RSD) [%]	RSDr [%]	RSD R [%]	R_E (RSD) [%]	RSDr [%]	RSD R [%]	R_E (RSD) [%]	RSDr [%]	RSD R [%]	R_E (RSD) [%]	RSDr [%]	RSD R [%]	R_E (RSD) [%]	RSDr [%]	RSD R [%]
3,4,5-trimethacarb	114 (8)	8.56	12.2	117 (12)	12.1	12.1	107 (4)	6.31	12.0	113 (4)	6.99	4.88	106 (5)	4.04	7.03
acephate	-	-	-	-	-	-	99 (3)	5.66	6.39	92 (5)	3.79	4.89	92 (5)	5.06	7.01
acetamiprid	100 (6)	8.16	6.09	84 (6)	7.26	8.20	103 (3)	6.94	6.11	96 (2)	6.46	2.55	105 (3)	4.82	4.03
acetochlor	-	-	-	-	-	-	103 (3)	3.37	11.3	100 (6)	11.4	7.72	97 (4)	18.6	5.32
alachlor	-	-	-	-	-	-	-	-	-	93 (5)	6.62	5.56	106 (2)	4.96	5.64
aldicarb	103 (8)	7.53	8.24	113 (9)	9.61	10.6	97 (4)	7.73	5.65	95 (3)	8.09	4.62	98 (3)	8.52	4.13
aldicarb-sulfoxide	91 (7)	10.8	5.55	106 (3)	8.37	7.70	96 (9)	6.82	6.48	99 (3)	3.37	3.80	98 (1)	3.98	3.90
aldoxycarb	98 (7)	6.17	8.85	94 (3)	5.79	4.93	94 (7)	6.71	8.63	103 (4)	6.75	5.07	110 (4)	5.39	3.64
ametoctradin	106 (8)	12.7	12.6	93 (7)	13.6	8.28	117 (5)	11.6	8.06	100 (5)	6.91	7.47	106 (4)	3.98	4.95
ametryn	95 (5)	8.10	5.39	108 (3)	8.13	7.66	99 (5)	6.59	6.83	107 (4)	4.58	5.63	98 (4)	4.34	3.76
amidosulfuron	92 (5)	7.37	8.04	101 (5)	4.62	9.14	102 (7)	6.26	7.12	90 (5)	8.83	5.85	93 (3)	8.38	3.98
aminocarb	102 (3)	6.87	10.2	109 (6)	6.84	10.2	104 (2)	6.10	11.4	110 (5)	5.43	6.34	110 (1)	5.04	5.99
aminopyralid	-	-	-	-	-	-	-	-	-	65 (5)	12.3	5.07	65 (2)	9.28	5.37
amitraz	-	-	-	-	-	-	-	-	-	24 (17)	59.0	30.4	25 (8)	65.5	27.6
amitraz-amide	-	-	-	-	-	-	101 (7)	8.52	7.95	102 (6)	6.61	4.46	110 (2)	3.10	5.72
amitraz-amidin	109 (5)	19.0	7.73	107 (5)	22.3	8.24	105 (7)	21.8	9.38	105 (5)	13.5	13.2	89 (14)	10.5	11.2
ancymidol	-	-	-	-	-	-	105 (5)	8.43	9.15	86 (5)	4.77	7.25	94 (4)	4.39	4.69
atrazine	95 (4)	10.3	5.22	110 (3)	5.20	7.97	101 (5)	6.09	5.31	96 (7)	6.88	5.54	100 (5)	6.82	3.70
atrazine-desethyl-desisopropyl	96 (7)	6.35	6.34	103 (4)	4.23	4.54	105 (5)	5.32	8.47	93 (3)	5.99	3.57	96 (3)	3.16	4.02
avermectin B1a	-	-	-	-	-	-	-	-	-	107 (13)	24.9	14.7	119 (10)	46.7	15.2
avermectin B1b	n.a.	n.a.	n.a.	n.a.	n.a.	n.a.	n.a.	n.a.	n.a.	n.a.	n.a.	n.a.	n.a.	n.a.	n.a.
azaconazole	102 (5)	6.76	9.94	92 (4)	8.21	7.66	101 (8)	6.02	7.19	99 (8)	7.14	6.94	97 (3)	4.34	3.38
azamethiphos	110 (6)	6.19	10.2	118 (4)	4.88	10.4	109 (5)	4.80	10.4	97 (4)	4.42	4.72	96 (3)	3.33	6.49
aziprotryne	105 (3)	16.0	14.0	102 (3)	12.9	6.62	112 (4)	6.84	6.42	110 (4)	6.57	7.24	107 (2)	5.21	4.11

Analyte	Spiking level 1			Spiking level 2			Spiking level 3			Spiking level 7			Spiking level 8		
	*R*E (RSD) [%]	RSDr [%]	RSD R [%]	*R*E (RSD) [%]	RSDr [%]	RSD R [%]	*R*E (RSD) [%]	RSDr [%]	RSD R [%]	*R*E (RSD) [%]	RSDr [%]	RSD R [%]	*R*E (RSD) [%]	RSDr [%]	RSD R [%]
azoxystrobin	92 (4)	7.77	4.34	107 (5)	5.55	6.37	99 (4)	5.88	3.67	93 (4)	4.53	6.26	100 (4)	4.80	3.82
benalaxyl	99 (3)	7.63	4.66	101 (7)	8.75	8.84	118 (3)	7.94	13.1	96 (4)	4.16	4.00	102 (3)	4.23	4.17
bendiocarb	-	-	-	-	-	-	42 (9)	11.8	30.4	425 (6)	10.1	41.5	436 (2)	4.69	41.7
benfuracarb	-	-	-	-	-	-	31 (6)	36.5	26.8	26 (13)	38.0	23.4	29 (6)	46.9	27.2
benodanil	99 (5)	7.53	8.48	111 (3)	11.0	8.31	105 (5)	7.40	7.74	95 (6)	8.15	6.53	95 (6)	4.25	4.99
benomyl	n.a.	n.a.	n.a.	n.a.	n.a.	n.a.	n.a.	n.a.	n.a.	n.a.	n.a.	n.a.	n.a.	n.a.	n.a.
bensulfuron-methyl	103 (4)	5.14	8.05	94 (5)	6.17	6.63	93 (3)	5.80	4.84	98 (5)	5.66	5.18	101 (3)	3.13	3.68
benthiavalicarb-isopropyl	102 (8)	11.3	9.70	105 (6)	7.92	8.94	103 (3)	5.66	5.06	97 (4)	5.68	3.89	94 (3)	4.93	4.83
bitertanol	-	-	-	-	-	-	102 (6)	12.8	12.0	97 (8)	11.4	10.7	98 (5)	8.25	8.21
boscalid	97 (8)	7.86	8.54	104 (5)	5.80	6.96	110 (5)	7.98	8.76	97 (7)	6.04	5.61	103 (2)	3.26	5.02
bromacil	102 (3)	5.92	7.83	110 (5)	7.28	8.65	134 (5)	7.29	20.2	99 (3)	6.30	2.36	101 (2)	3.51	3.11
bromuconazole	-	-	-	-	-	-	99 (8)	7.38	6.34	94 (4)	6.29	7.26	97 (4)	2.88	4.75
bupirimate	96 (8)	8.29	9.35	99 (5)	6.64	5.86	112 (4)	5.84	8.50	99 (5)	5.59	5.27	102 (3)	5.07	3.66
buprofezin	105 (5)	9.14	7.79	108 (7)	9.23	7.06	104 (6)	6.68	8.83	102 (6)	5.49	5.95	102 (3)	5.44	4.02
butachlor	-	-	-	-	-	-	111 (6)	6.04	12.4	92 (5)	8.92	6.19	102 (4)	14.0	6.48
butocarboxim	95 (7)	7.57	7.66	106 (7)	7.72	9.94	105 (6)	5.02	6.36	96 (3)	6.48	4.71	92 (2)	9.64	4.42
butocarboxim-sulfoxide	98 (4)	6.24	5.58	107 (3)	6.14	7.06	101 (3)	3.72	8.68	94 (3)	3.96	3.12	98 (3)	3.52	3.58
butoxycarboxim	97 (8)	7.80	7.38	111 (7)	7.00	8.19	103 (3)	5.42	8.68	100 (4)	4.65	5.73	103 (2)	5.41	4.99
buturon	109 (9)	7.38	11.9	110 (7)	8.59	7.20	113 (7)	6.60	11.1	104 (3)	4.54	6.02	99 (3)	2.71	3.81
cadusafos	97 (5)	7.40	8.06	108 (6)	8.07	10.0	110 (5)	6.56	9.75	97 (2)	4.18	5.24	106 (4)	4.40	3.93
carbaryl	39 (6)	9.44	71.3	35 (14)	13.2	60.8	29 (4)	9.53	47.0	1,088 (8)	6.95	22.3	1,094 (6)	5.81	17.8
carbendazim	88 (1)	5.77	4.22	107 (3)	5.18	6.75	95 (4)	5.79	6.07	88 (5)	5.36	5.41	92 (2)	2.20	4.47
carbofuran	111 (5)	7.94	22.5	111 (6)	7.91	19.7	109 (3)	8.34	17.9	170 (4)	4.57	7.01	163 (2)	2.04	8.45
carbofuran-3-hydroxy	91 (6)	9.99	66.3	100 (13)	10.7	67.5	84 (8)	7.74	66.8	389 (6)	6.09	8.84	376 (9)	6.55	10.1
carbosulfan	11 (16)	37.7	35.4	12 (8)	41.3	36.3	14 (6)	46.0	22.1	10 (15)	50.5	40.0	10 (8)	67.9	46.6

Analyte	Spiking level 1			Spiking level 2			Spiking level 3			Spiking level 7			Spiking level 8		
	R_E (RSD) [%]	RSDr [%]	RSD R [%]	R_E (RSD) [%]	RSDr [%]	RSD R [%]	R_E (RSD) [%]	RSDr [%]	RSD R [%]	R_E (RSD) [%]	RSDr [%]	RSD R [%]	R_E (RSD) [%]	RSDr [%]	RSD R [%]
carboxin	93 (4)	7.69	5.47	102 (3)	6.27	4.05	109 (5)	7.25	10.1	104 (4)	4.48	6.59	106 (2)	3.72	3.72
chlorantraniliprole	105 (12)	9.57	9.23	107 (10)	10.6	11.7	110 (8)	7.25	6.77	108 (4)	6.75	4.16	100 (3)	3.48	5.34
chlorbromuron	90 (5)	9.22	6.21	116 (6)	7.50	14.1	110 (10)	7.61	12.5	97 (5)	5.77	4.83	99 (4)	3.65	5.33
chlorfluazuron	97 (8)	8.96	10.2	107 (4)	8.54	6.03	113 (5)	9.64	10.5	97 (5)	9.68	6.63	95 (4)	6.51	4.63
chloridazon	105 (4)	8.93	11.7	107 (10)	8.33	10.6	113 (3)	4.83	11.5	94 (10)	8.15	7.08	99 (2)	3.11	3.88
chloroxuron	99 (14)	12.8	10.7	101 (6)	9.45	10.4	105 (11)	12.4	11.6	89 (5)	7.73	7.32	113 (7)	7.24	7.60
chlorpropham	-	-	-	-	-	-	-	-	-	100 (6)	8.15	11.0	93 (7)	7.58	8.21
chlorsulfuron	90 (6)	9.11	9.95	93 (12)	7.81	12.1	114 (6)	6.46	8.61	91 (4)	7.33	4.34	99 (2)	8.36	10.6
chlortoluron	102 (2)	8.08	4.77	104 (2)	7.68	4.40	100 (5)	5.44	7.11	100 (3)	4.97	3.71	95 (3)	3.38	3.56
cinidon-ethyl	-	-	-	-	-	-	109 (2)	6.81	8.26	95 (2)	6.16	10.8	94 (1)	1.69	9.61
cinosulfuron	104 (3)	4.95	6.98	98 (7)	6.28	8.53	99 (3)	6.40	8.07	98 (4)	7.00	5.82	97 (5)	3.93	4.47
clethodim	111 (10)	9.07	9.81	113 (9)	11.7	9.22	100 (12)	10.6	10.6	100 (7)	5.65	6.56	95 (8)	6.30	8.07
clodinafop-propargyl	100 (5)	7.35	5.66	103 (2)	5.91	4.30	114 (5)	7.40	9.84	97 (4)	4.92	3.99	101 (2)	2.65	3.02
clofentezine	110 (10)	9.74	9.80	105 (3)	8.98	9.91	97 (3)	8.94	8.51	104 (5)	7.29	5.54	101 (4)	3.77	4.23
clomazone	99 (4)	8.24	10.7	108 (4)	7.82	6.09	102 (7)	6.99	6.06	101 (7)	6.34	5.63	101 (1)	4.01	3.48
clothianidin	-	-	-	-	-	-	98 (6)	6.64	8.49	88 (8)	6.26	9.61	95 (4)	4.12	3.86
cyanazine	88 (12)	12.5	13.3	93 (5)	7.05	8.59	113 (9)	8.26	8.58	102 (5)	6.06	4.54	97 (3)	4.34	4.09
cyazofamid	-	-	-	-	-	-	106 (5)	8.54	7.53	94 (5)	6.60	8.69	108 (4)	5.48	5.37
cymoxanil	-	-	-	-	-	-	-	-	-	204 (5)	7.87	21.5	197 (7)	6.63	18.0
cyproconazole	100 (4)	5.85	5.85	110 (4)	3.79	9.06	114 (5)	4.55	12.0	100 (3)	5.44	4.53	103 (3)	4.49	3.81
cyprodinil	-	-	-	-	-	-	105 (7)	9.38	9.44	105 (4)	6.03	6.07	98 (3)	6.77	3.62
cyprofuram	102 (5)	7.77	7.16	105 (6)	11.2	7.59	95 (9)	10.1	6.49	96 (4)	6.11	5.36	99 (2)	3.84	4.80
cyromazine	99 (10)	14.8	16.2	87 (6)	13.7	7.26	84 (6)	16.6	12.4	85 (10)	10.9	9.18	81 (5)	9.65	4.56
daminozide	-	-	-	-	-	-	-	-	-	84 (5)	6.20	6.66	84 (3)	3.32	12.9
demeton	95 (9)	11.0	8.05	107 (2)	10.4	9.61	93 (5)	8.61	5.69	95 (4)	5.52	5.32	106 (4)	5.98	6.55
demeton-S-methyl	104 (9)	8.77	7.38	97 (6)	7.33	7.42	95 (6)	7.16	4.46	93 (4)	7.13	5.96	93 (4)	4.19	4.91

Analyte	Spiking level 1			Spiking level 2			Spiking level 3			Spiking level 7			Spiking level 8		
	R_E (RSD) [%]	RSDr [%]	RSD R [%]	R_E (RSD) [%]	RSDr [%]	RSD R [%]	R_E (RSD) [%]	RSDr [%]	RSD R [%]	R_E (RSD) [%]	RSDr [%]	RSD R [%]	R_E (RSD) [%]	RSDr [%]	RSD R [%]
demeton-S-methyl-sulfone	96 (6)	6.65	5.96	108 (7)	5.19	7.93	105 (4)	3.32	11.3	93 (5)	4.99	7.36	96 (2)	5.17	6.50
desmedipham	n.a.	n.a.	n.a.	n.a.	n.a.	n.a.	n.a.	n.a.	n.a.	n.a.	n.a.	n.a.	n.a.	n.a.	n.a.
desmetryn	99 (6)	6.77	7.44	107 (4)	5.76	6.29	103 (4)	6.18	5.97	98 (3)	4.56	4.04	103 (2)	3.15	3.14
diazinon	95 (3)	7.59	8.74	91 (6)	8.19	6.72	103 (5)	6.14	7.08	98 (3)	6.55	3.46	103 (4)	6.92	6.43
dichlorvos	-	-	-	-	-	-	-	-	-	92 (4)	5.78	7.26	104 (2)	2.39	3.14
diclobutrazol	-	-	-	-	-	-	-	-	-	102 (5)	7.18	5.26	106 (2)	3.64	5.87
diethofencarb	100 (6)	16.3	8.71	103 (5)	12.4	9.49	101 (13)	14.2	9.20	97 (10)	9.76	9.60	89 (7)	5.54	7.07
diethyltoluamide	101 (3)	12.7	8.57	99 (6)	11.2	5.43	99 (4)	8.88	6.07	104 (4)	4.38	6.14	100 (1)	2.67	3.07
difenoconazole	94 (6)	7.62	6.78	111 (6)	8.58	7.63	109 (5)	10.7	7.60	93 (10)	8.88	6.49	96 (6)	6.76	5.93
difenoxuron	92 (5)	13.1	7.05	87 (4)	10.3	8.08	99 (3)	6.35	4.76	91 (7)	4.94	5.97	109 (5)	6.02	6.63
diflubenzuron	97 (3)	7.44	7.79	112 (8)	7.73	7.60	108 (6)	8.07	9.88	99 (5)	6.20	4.15	102 (3)	3.03	4.07
diflufenican	110 (5)	15.4	9.31	113 (8)	11.3	10.1	115 (9)	11.8	12.3	98 (4)	8.24	5.96	101 (5)	7.18	4.23
dimefox	-	-	-	-	-	-	-	-	-	97 (5)	6.12	5.58	100 (5)	4.42	7.19
dimefuron	96 (4)	9.74	7.05	99 (4)	7.20	9.88	92 (3)	11.2	6.47	99 (6)	7.41	5.63	103 (5)	7.51	6.96
dimethenamid	111 (6)	8.41	12.4	112 (8)	7.49	12.8	109 (4)	4.80	9.26	100 (4)	4.59	4.81	102 (2)	6.13	4.73
dimethoate	102 (4)	6.13	8.21	102 (5)	6.01	4.14	107 (4)	5.37	7.04	97 (3)	5.47	3.78	105 (3)	4.20	3.75
dimethomorph	110 (5)	9.40	10.2	104 (3)	10.1	3.99	112 (3)	7.22	12.3	103 (4)	6.44	5.23	103 (2)	3.40	5.61
dimetilan	94 (3)	9.67	4.64	105 (4)	8.58	9.36	104 (6)	6.66	11.2	96 (5)	4.54	3.83	103 (2)	3.14	3.02
dimoxystrobin	107 (4)	5.77	9.00	108 (8)	8.04	7.52	102 (7)	9.25	10.2	97 (3)	6.84	4.79	101 (2)	3.90	3.52
dinotefuran	99 (5)	7.60	6.55	107 (8)	8.39	8.24	103 (5)	5.77	9.20	100 (4)	4.63	5.08	96 (2)	3.13	3.20
disulfoton	89 (5)	15.1	17.0	102 (10)	11.7	8.15	111 (11)	13.0	14.1	97 (3)	6.37	3.63	101 (3)	5.23	8.23
disulfoton-sulfone	100 (6)	10.0	10.5	103 (7)	7.38	10.8	101 (10)	7.83	11.2	90 (4)	5.96	5.78	97 (3)	5.35	8.72
disulfoton-sulfoxide	107 (4)	8.49	6.72	109 (5)	6.51	8.44	109 (2)	4.87	11.3	91 (4)	5.48	8.03	109 (3)	6.32	6.20
diuron	98 (9)	8.43	6.78	98 (7)	8.54	6.99	100 (5)	7.54	7.08	94 (5)	5.83	5.12	102 (3)	3.94	5.40
dodine	n.a.	n.a.	n.a.	n.a.	n.a.	n.a.	n.a.	n.a.	n.a.	n.a.	n.a.	n.a.	n.a.	n.a.	n.a.
emamectin B1a	94 (13)	8.14	11.8	112 (6)	8.85	14.8	97 (18)	18.2	10.5	109 (19)	25.2	14.6	84 (13)	44.2	16.6
emamectin B1b	96 (20)	18.0	31.5	104 (14)	16.7	23.4	127 (21)	23.7	20.7	99 (22)	25.4	19.0	87 (17)	44.3	30.1

Analyte	Spiking level 1			Spiking level 2			Spiking level 3			Spiking level 7			Spiking level 8		
	R_E (RSD) [%]	RSDr [%]	RSD R [%]	R_E (RSD) [%]	RSDr [%]	RSD R [%]	R_E (RSD) [%]	RSDr [%]	RSD R [%]	R_E (RSD) [%]	RSDr [%]	RSD R [%]	R_E (RSD) [%]	RSDr [%]	RSD R [%]
epoxiconazole	94 (9)	9.54	10.4	113 (2)	7.29	13.0	85 (8)	10.3	10.1	94 (5)	6.24	4.46	103 (3)	3.10	3.82
ethiofencarb	84 (15)	13.2	88.2	96 (12)	14.3	87.7	100 (7)	9.63	88.9	594 (6)	6.59	34.3	544 (6)	4.45	21.1
ethiofencarb-sulfone	-	-	-	-	-	-	-	-	-	322 (2)	7.93	84.3	337 (5)	6.82	80.4
ethiofencarb-sulfoxide	-	-	-	-	-	-	-	-	-	783 (3)	5.44	81.4	876 (4)	6.33	81.7
ethiprole	107 (9)	12.2	11.5	100 (5)	7.79	7.94	103 (3)	7.84	8.60	96 (5)	7.95	5.12	99 (4)	4.59	4.90
ethofumesate	106 (14)	11.3	14.1	103 (5)	11.3	6.25	96 (9)	7.07	8.34	96 (5)	6.94	6.02	100 (3)	6.21	2.82
ethofumesate-2-keto	-	-	-	-	-	-	-	-	-	90 (7)	7.37	12.5	100 (4)	5.33	4.79
ethoprofos	105 (7)	11.5	8.85	101 (9)	10.4	6.80	98 (3)	7.62	5.47	97 (8)	5.40	6.54	97 (2)	3.27	4.89
etofenprox	103 (10)	16.5	10.8	100 (7)	15.8	7.87	106 (7)	16.4	9.86	106 (10)	9.41	8.31	106 (4)	7.11	5.21
etoxazole	97 (8)	8.96	7.97	103 (3)	8.43	4.14	99 (6)	8.46	6.61	97 (4)	5.01	4.37	95 (4)	3.14	3.67
famoxadon	107 (7)	9.56	8.55	115 (11)	9.82	12.2	119 (6)	7.70	15.2	97 (4)	5.97	6.72	95 (3)	2.94	7.21
fenamidone	99 (4)	5.28	4.88	99 (3)	7.41	4.45	104 (3)	7.30	8.38	103 (5)	5.15	7.36	101 (6)	4.78	4.08
fenamiphos	104 (7)	8.05	12.4	109 (4)	8.10	5.92	113 (4)	5.48	13.3	96 (5)	6.13	5.95	98 (2)	3.69	3.70
fenamiphos-sulfone	116 (9)	10.4	14.4	97 (7)	5.93	11.1	112 (4)	7.41	10.9	97 (4)	5.77	8.12	101 (2)	5.00	5.81
fenamiphos-sulfoxide	94 (12)	11.4	10.4	115 (5)	9.35	15.7	106 (5)	7.54	7.61	94 (3)	5.93	6.38	102 (4)	6.39	5.47
fenarimol	-	-	-	-	-	-	105 (5)	5.73	12.7	95 (3)	5.05	3.96	93 (1)	4.04	4.31
fenazaquin	98 (3)	11.3	2.85	108 (3)	10.7	8.07	119 (2)	9.40	11.5	98 (4)	6.22	3.69	101 (2)	2.25	4.29
fenbuconazole	105 (7)	13.7	13.5	104 (6)	9.67	8.07	110 (6)	7.85	12.8	102 (6)	6.17	5.01	103 (3)	3.38	8.26
fenhexamid	-	-	-	-	-	-	110 (8)	7.91	16.8	110 (9)	7.57	9.14	97 (4)	4.56	5.66
fenobucarb	106 (6)	13.8	11.9	96 (9)	13.2	9.19	102 (5)	9.01	11.5	116 (6)	8.68	6.69	116 (3)	4.06	9.05
fenoxaprop-P-ethyl	-	-	-	-	-	-	116 (3)	5.46	9.56	93 (5)	5.60	4.36	101 (1)	3.30	2.86
fenoxycarb	103 (7)	9.77	8.29	96 (7)	7.86	6.37	104 (7)	9.70	8.65	96 (2)	6.55	3.65	97 (3)	2.77	3.99
fenpiclonil	103 (8)	8.92	15.0	90 (7)	9.67	9.08	90 (12)	8.06	8.24	92 (5)	7.23	6.97	101 (5)	6.59	6.86
fenpropidin	99 (6)	11.8	5.59	106 (3)	10.5	3.55	103 (5)	11.3	10.6	105 (3)	9.26	5.34	98 (3)	3.76	4.57
fenpropimorph	99 (4)	12.2	7.56	98 (4)	13.0	3.63	111 (5)	10.1	10.4	105 (5)	5.82	5.68	102 (2)	3.37	3.39

Analyte	Spiking level 1			Spiking level 2			Spiking level 3			Spiking level 7			Spiking level 8		
	R_E (RSD) [%]	RSDr [%]	RSD R [%]	R_E (RSD) [%]	RSDr [%]	RSD R [%]	R_E (RSD) [%]	RSDr [%]	RSD R [%]	R_E (RSD) [%]	RSDr [%]	RSD R [%]	R_E (RSD) [%]	RSDr [%]	RSD R [%]
fenpyroximate	102 (2)	10.3	5.31	100 (2)	9.80	5.59	118 (3)	16.1	8.82	98 (3)	7.04	3.12	102 (1)	2.57	2.14
fensulfothion	109 (8)	7.07	8.85	106 (7)	5.70	7.54	98 (3)	4.83	5.46	96 (2)	5.33	4.31	102 (3)	4.47	4.55
fensulfothion-PO-sulfone	99 (5)	6.35	7.75	103 (4)	5.24	4.45	100 (4)	4.17	5.86	94 (3)	5.92	3.17	100 (3)	4.39	2.95
fensulfothion-PO-sulfoxide	96 (7)	10.6	14.8	95 (4)	8.75	7.11	101 (7)	6.46	8.55	95 (6)	6.40	4.96	97 (5)	6.30	5.92
fensulfothion-PS-sulfone	103 (4)	4.39	8.10	91 (5)	5.27	6.16	115 (4)	3.80	14.6	101 (3)	6.08	4.71	102 (1)	6.00	3.80
fenthion	-	-	-	-	-	-	106 (7)	10.4	6.30	97 (4)	7.89	6.96	107 (5)	3.79	5.95
fenthion-oxon	99 (6)	7.61	7.27	93 (4)	5.78	8.64	103 (4)	5.65	6.96	97 (5)	5.10	4.17	103 (4)	6.79	4.55
fenthion-PO-sulfone	90 (8)	9.15	8.23	99 (8)	10.3	15.4	101 (8)	7.22	7.64	94 (5)	7.91	4.93	100 (4)	7.06	4.84
fenthion-PO-sulfoxide	99 (10)	9.85	9.08	99 (6)	7.35	8.22	103 (5)	6.77	8.03	100 (6)	7.92	5.50	96 (3)	6.89	5.16
fenthion-PS-sulfone	-	-	-	-	-	-	95 (12)	11.0	11.9	93 (5)	7.69	6.67	106 (3)	5.75	8.14
fenthion-PS-sulfoxide	106 (5)	7.42	9.43	113 (4)	4.85	8.49	112 (4)	4.22	8.23	92 (4)	4.35	5.41	99 (2)	5.32	5.56
fenuron	-	-	-	-	-	-	-	-	-	100 (4)	4.56	4.81	101 (1)	2.16	2.58
flazasulfuron	92 (8)	6.82	8.17	100 (8)	6.53	7.25	97 (4)	6.77	6.44	89 (5)	4.85	7.25	93 (5)	4.42	5.60
flonicamid	113 (15)	12.2	14.4	93 (6)	9.51	6.15	110 (2)	10.6	9.32	91 (4)	5.34	7.34	97 (3)	2.58	4.61
florasulam	92 (10)	7.25	9.83	116 (6)	6.13	11.3	107 (8)	7.32	11.8	106 (13)	8.25	9.96	94 (4)	6.56	6.22
fluazifop-P-butyl	104 (5)	8.26	13.4	103 (6)	8.85	6.02	109 (8)	7.00	9.20	96 (6)	6.61	6.66	99 (2)	3.39	4.59
fluazuron	116 (13)	15.1	14.6	101 (5)	17.3	13.8	103 (11)	20.5	8.96	94 (9)	15.1	12.3	114 (4)	13.6	9.26
flucycloxuron	102 (3)	7.71	4.48	110 (5)	7.78	5.00	113 (4)	7.79	7.85	98 (7)	7.35	5.85	101 (3)	2.39	3.33
fludioxonil	-	-	-	-	-	-	110 (4)	9.12	9.05	95 (5)	6.11	5.76	94 (5)	4.15	8.59
flufenacet	102 (6)	7.13	10.1	100 (4)	6.71	5.36	103 (6)	6.34	6.83	104 (7)	6.91	6.02	102 (4)	3.86	6.93
flufenoxuron	103 (13)	11.9	11.4	119 (6)	16.4	12.8	109 (15)	13.3	13.7	88 (8)	12.5	8.66	108 (5)	7.85	13.0
fluometuron	-	-	-	-	-	-	109 (3)	7.88	7.23	101 (9)	6.75	6.88	100 (1)	2.62	2.55
fluopicolide	102 (8)	8.35	9.15	105 (6)	7.02	6.87	114 (6)	7.30	11.2	96 (5)	5.12	4.69	102 (4)	2.61	4.67
flurochloridone	99 (5)	7.75	5.58	115 (4)	6.58	10.3	102 (4)	4.52	9.85	90 (3)	5.56	6.82	100 (2)	4.33	6.54

Analyte	Spiking level 1			Spiking level 2			Spiking level 3			Spiking level 7			Spiking level 8		
	R_E (RSD) [%]	RSDr [%]	RSD R [%]	R_E (RSD) [%]	RSDr [%]	RSD R [%]	R_E (RSD) [%]	RSDr [%]	RSD R [%]	R_E (RSD) [%]	RSDr [%]	RSD R [%]	R_E (RSD) [%]	RSDr [%]	RSD R [%]
flurprimidol	84 (10)	8.27	13.0	110 (11)	8.31	11.5	102 (7)	6.62	7.60	92 (7)	6.71	6.10	93 (3)	3.07	5.56
flusilazole	96 (4)	5.57	6.40	105 (4)	6.99	10.5	104 (4)	7.08	7.38	96 (5)	5.40	5.21	104 (4)	3.35	4.42
flutriafol	-	-	-	-	-	-	-	-	-	96 (5)	5.76	3.91	95 (3)	4.45	3.27
formetanate	105 (8)	9.93	27.1	108 (7)	8.20	26.6	91 (11)	11.1	18.9	170 (11)	9.92	11.7	183 (6)	6.74	8.58
fosthiazate	100 (6)	5.62	6.31	101 (3)	6.36	6.32	100 (2)	4.45	5.75	99 (4)	5.02	4.04	102 (4)	5.05	3.99
fuberidazole	103 (7)	6.02	7.02	99 (5)	4.44	4.76	107 (3)	4.67	8.95	101 (7)	5.70	6.63	105 (2)	3.38	5.06
furathiocarb	215 (6)	9.50	25.3	214 (7)	9.43	22.3	205 (5)	11.0	20.4	173 (10)	7.54	9.27	169 (3)	4.07	5.55
halofenozide	96 (4)	5.42	7.72	112 (8)	6.90	12.3	104 (6)	5.58	6.81	94 (7)	6.20	8.97	93 (3)	8.59	4.87
haloxyfop	89 (18)	19.9	13.2	113 (11)	21.7	13.2	106 (9)	23.2	10.3	93 (4)	5.51	9.33	93 (2)	6.09	5.47
haloxyfop-2-ethoxyethyl	99 (7)	9.81	11.2	107 (5)	11.7	7.07	109 (6)	8.45	7.68	100 (5)	7.60	5.97	103 (3)	3.37	4.46
haloxyfop-methyl	94 (5)	5.95	3.69	104 (4)	5.93	5.53	117 (2)	13.3	10.6	97 (3)	3.61	3.13	101 (1)	1.69	2.75
hexaconazole	99 (8)	7.94	7.98	99 (6)	6.80	8.41	95 (6)	7.95	5.83	99 (4)	5.71	4.81	102 (3)	2.92	4.40
hexaflumuron	-	-	-	-	-	-	-	-	-	95 (3)	8.86	6.09	117 (9)	13.4	12.0
hexazinone	103 (6)	8.88	7.30	103 (4)	9.71	5.33	102 (4)	6.95	5.60	102 (3)	4.18	4.65	104 (2)	3.25	3.93
hexythiazox	98 (6)	8.28	7.52	109 (4)	8.78	8.16	112 (4)	9.03	10.7	97 (6)	7.14	4.59	98 (3)	3.93	2.87
imazalil	109 (9)	9.08	12.1	113 (6)	7.91	11.5	118 (5)	5.88	9.13	97 (6)	6.91	6.08	106 (3)	6.09	3.45
imibenconazole	94 (10)	9.03	8.24	110 (2)	12.7	8.99	109 (8)	8.36	8.53	90 (6)	7.60	7.54	98 (4)	7.65	6.78
imidacloprid	108 (14)	11.4	14.2	119 (12)	7.95	15.9	105 (4)	7.43	13.3	93 (6)	9.75	6.39	102 (4)	4.04	6.07
indoxacarb	84 (5)	8.99	11.2	114 (8)	8.97	10.7	113 (8)	9.98	11.2	92 (1)	7.76	3.98	101 (6)	5.27	5.14
iodosulfuron-methyl	-	-	-	-	-	-	97 (7)	6.55	8.14	95 (4)	6.86	5.71	92 (3)	4.58	6.67
iprovalicarb	100 (8)	10.8	6.92	98 (7)	11.0	6.23	108 (4)	10.6	10.7	107 (7)	8.08	7.11	102 (3)	2.54	4.07
isoprocarb	-	-	-	-	-	-	120 (7)	7.77	15.5	108 (6)	9.71	11.5	112 (2)	5.91	8.67
isoprothiolane	110 (4)	5.07	12.2	101 (7)	7.78	4.78	91 (3)	3.26	2.89	100 (5)	5.01	4.58	97 (2)	4.12	2.76
isoproturon	-	-	-	-	-	-	115 (7)	8.23	9.87	107 (6)	3.97	5.09	100 (1)	3.06	2.71
isoxaben	86 (5)	9.51	8.71	104 (2)	8.38	8.48	98 (3)	8.70	5.96	96 (5)	5.81	5.21	98 (3)	5.16	4.66
isoxaflutole	n.a.	n.a.	n.a.	n.a.	n.a.	n.a.	n.a.	n.a.	n.a.	n.a.	n.a.	n.a.	n.a.	n.a.	n.a.
isoxathion	95 (3)	10.6	4.44	106 (5)	7.62	7.48	98 (4)	6.45	5.24	95 (3)	5.61	4.72	104 (2)	3.97	3.99

Analyte	Spiking level 1			Spiking level 2			Spiking level 3			Spiking level 7			Spiking level 8		
	R_E (RSD) [%]	RSDr [%]	RSD R [%]	R_E (RSD) [%]	RSDr [%]	RSD R [%]	R_E (RSD) [%]	RSDr [%]	RSD R [%]	R_E (RSD) [%]	RSDr [%]	RSD R [%]	R_E (RSD) [%]	RSDr [%]	RSD R [%]
lenacil	109 (4)	9.65	8.67	111 (2)	11.1	10.0	100 (6)	11.2	7.74	99 (3)	5.29	5.60	95 (3)	4.84	6.27
linuron	100 (4)	12.5	7.60	103 (10)	9.81	8.42	92 (9)	7.43	7.85	93 (6)	5.77	5.09	102 (2)	4.40	4.87
lufenuron	108 (13)	11.3	17.4	110 (14)	19.9	15.6	111 (12)	17.6	11.4	88 (12)	13.8	12.1	99 (6)	14.0	12.5
malaoxon	100 (4)	6.13	10.7	115 (9)	8.07	8.57	107 (1)	4.99	10.4	104 (6)	4.86	6.19	102 (3)	2.86	2.79
malathion	109 (6)	7.09	10.3	107 (11)	8.30	8.46	102 (3)	7.62	5.01	99 (4)	5.87	5.74	107 (4)	4.32	5.14
mandipropamid	103 (5)	7.24	11.3	99 (6)	6.37	7.55	102 (4)	9.67	9.41	100 (7)	7.40	6.63	107 (4)	5.64	6.42
mepanipyrim	-	-	-	-	-	-	-	-	-	108 (5)	6.28	8.66	96 (1)	5.65	5.14
metalaxyl	102 (3)	6.45	7.09	112 (6)	7.92	9.43	99 (7)	7.88	5.79	94 (6)	5.05	4.11	102 (3)	5.53	4.94
metamitron	104 (12)	12.0	12.3	113 (7)	6.76	14.9	105 (9)	8.07	16.9	105 (5)	6.17	7.17	103 (6)	4.17	5.68
metazachlor	106 (4)	4.80	8.98	113 (5)	4.65	10.8	104 (5)	4.65	10.8	98 (5)	6.50	4.81	97 (3)	6.64	3.29
metconazole	84 (9)	17.5	13.2	90 (13)	13.0	15.1	112 (8)	12.7	17.4	103 (6)	5.09	5.77	103 (2)	3.83	4.87
methabenzthiazuron	87 (6)	9.13	8.94	104 (3)	7.77	5.78	103 (7)	7.94	8.75	98 (4)	6.09	4.50	102 (2)	3.95	3.95
methacrifos	-	-	-	-	-	-	-	-	-	107 (6)	8.04	8.43	102 (4)	3.96	5.00
methamidophos	-	-	-	-	-	-	94 (1)	5.67	5.14	90 (2)	8.07	3.34	92 (1)	8.15	2.56
methidathion	97 (10)	9.64	10.3	118 (5)	5.69	13.1	103 (6)	6.79	8.98	92 (6)	8.38	5.53	101 (3)	6.49	3.46
methiocarb	-	-	-	-	-	-	53 (3)	8.78	81.0	1,279 (6)	7.32	13.9	1,121 (4)	4.97	5.78
methiocarb-sulfone	-	-	-	-	-	-	-	-	-	332 (5)	5.88	78.4	348 (3)	4.98	80.6
methiocarb-sulfoxide	-	-	-	-	-	-	-	-	-	923 (7)	10.5	86.8	785 (6)	9.28	74.0
metholachlor	112 (5)	9.54	12.3	111 (7)	7.06	6.03	108 (5)	7.15	9.80	94 (6)	5.84	4.19	100 (5)	5.54	5.05
methomyl	91 (10)	22.8	11.3	83 (8)	17.9	10.7	92 (6)	15.4	9.98	81 (11)	10.8	9.97	75 (6)	6.20	4.99
methoprotryn	102 (5)	7.55	7.87	104 (4)	7.35	7.41	99 (7)	6.75	6.80	97 (4)	5.47	3.38	99 (3)	4.34	5.75
methoxyfenozide	100 (6)	14.4	9.69	108 (7)	11.8	9.64	105 (4)	8.39	11.6	110 (6)	7.51	10.2	93 (4)	4.29	5.19
metobromuron	88 (11)	9.48	7.82	102 (8)	9.82	7.41	102 (8)	6.69	7.34	89 (8)	6.78	7.02	99 (3)	4.76	6.45
metolcarb	-	-	-	-	-	-	-	-	-	221 (6)	7.70	14.2	247 (3)	4.72	9.06
metoxuron	95 (5)	11.8	9.04	114 (10)	11.2	13.3	98 (9)	12.3	9.26	102 (7)	5.34	5.80	101 (2)	7.75	3.42
metrafenone	105 (4)	7.24	10.6	107 (3)	7.41	6.64	113 (5)	5.67	12.0	102 (5)	6.47	5.55	104 (2)	3.72	4.70

Analyte	Spiking level 1			Spiking level 2			Spiking level 3			Spiking level 7			Spiking level 8		
	R_E (RSD) [%]	RSDr [%]	RSD R [%]	R_E (RSD) [%]	RSDr [%]	RSD R [%]	R_E (RSD) [%]	RSDr [%]	RSD R [%]	R_E (RSD) [%]	RSDr [%]	RSD R [%]	R_E (RSD) [%]	RSDr [%]	RSD R [%]
metribuzin	101 (5)	7.38	7.64	97 (12)	10.1	10.3	107 (8)	9.69	13.4	87 (4)	5.04	6.42	101 (3)	2.93	4.73
metsulfuron-methyl	100 (19)	13.5	17.6	112 (9)	9.47	10.9	113 (12)	13.0	11.5	103 (5)	5.90	9.55	91 (6)	6.47	8.22
molinate	-	-	-	-	-	-	-	-	-	105 (5)	7.49	7.98	106 (6)	7.01	4.90
monocrotophos	111 (6)	20.5	12.5	103 (5)	19.5	4.77	95 (3)	17.2	3.10	116 (10)	9.41	11.1	107 (3)	4.51	4.76
monolinuron	97 (3)	8.17	7.60	116 (6)	6.78	7.83	100 (6)	6.96	7.36	95 (4)	5.08	3.96	97 (6)	5.38	5.00
monuron	108 (15)	12.4	16.2	91 (11)	16.4	12.0	87 (13)	11.7	12.2	94 (4)	5.23	6.01	97 (6)	7.18	5.91
napropamide	116 (5)	6.50	14.5	106 (6)	9.39	5.46	106 (7)	7.10	10.1	103 (5)	5.54	5.16	108 (3)	4.56	6.04
neburon	106 (12)	10.7	9.57	120 (9)	7.92	13.5	119 (9)	7.44	13.1	95 (4)	4.68	5.60	99 (4)	5.88	3.87
nicosulfuron	110 (6)	6.47	9.19	101 (4)	3.96	6.04	97 (4)	4.43	7.42	86 (7)	7.55	6.28	93 (6)	6.83	5.78
novaluron	118 (9)	10.5	16.5	104 (5)	10.4	16.5	110 (7)	18.7	9.48	95 (5)	11.0	8.89	113 (4)	9.94	8.17
nuarimol	-	-	-	-	-	-	102 (6)	7.98	10.9	102 (6)	6.01	7.13	105 (4)	5.51	4.49
ofurace	94 (6)	6.97	6.83	105 (10)	9.12	9.03	99 (5)	4.08	9.28	106 (5)	7.13	5.41	106 (2)	5.71	5.90
omethoate	97 (3)	6.08	6.34	105 (4)	4.39	7.84	100 (3)	3.56	6.99	96 (2)	3.53	3.45	98 (3)	3.44	4.41
orbencarb	82 (13)	14.8	13.9	103 (6)	9.37	8.63	119 (4)	8.34	16.7	97 (5)	5.90	4.92	101 (2)	3.86	4.10
oxadixyl	100 (7)	9.08	6.13	119 (3)	7.39	9.10	99 (4)	7.68	6.18	108 (9)	10.8	12.4	116 (10)	11.1	15.3
oxamyl	107 (3)	6.64	5.45	113 (3)	6.59	9.89	102 (2)	9.36	8.13	93 (3)	5.73	9.34	92 (3)	5.71	8.27
oxamyl-oxime	101 (9)	11.9	6.20	87 (7)	12.1	17.4	93 (9)	7.86	14.4	108 (7)	6.28	5.15	111 (2)	4.22	3.30
oxydemeton-methyl	108 (5)	18.0	9.23	100 (4)	17.0	4.41	98 (3)	14.3	3.99	102 (8)	7.64	7.25	102 (2)	2.82	3.94
paclobutrazol	-	-	-	-	-	-	108 (4)	4.55	6.38	101 (2)	5.42	4.60	95 (4)	3.84	3.28
paraoxon-ethyl	88 (5)	6.98	9.98	111 (4)	5.61	9.12	109 (8)	5.89	9.04	98 (4)	6.06	6.19	106 (3)	3.97	3.72
paraoxon-methyl	-	-	-	-	-	-	103 (4)	6.40	12.1	97 (3)	7.38	4.92	94 (1)	7.63	6.51
penconazole	95 (11)	7.91	7.97	100 (3)	5.79	10.8	100 (2)	5.93	8.47	93 (6)	6.03	4.03	100 (3)	3.63	4.99
pencycuron	99 (8)	8.92	8.60	102 (5)	7.22	5.91	120 (6)	7.89	13.9	97 (4)	4.77	4.34	100 (3)	4.44	3.69
pendimethalin	-	-	-	-	-	-	-	-	-	93 (6)	7.59	6.50	94 (2)	3.27	4.50
pentanochlor	-	-	-	-	-	-	103 (4)	7.47	6.89	100 (5)	5.66	5.47	95 (3)	2.82	4.44
phenmedipham	n.a.	n.a.	n.a.	n.a.	n.a.	n.a.	n.a.	n.a.	n.a.	n.a.	n.a.	n.a.	n.a.	n.a.	n.a.
phorate	-	-	-	-	-	-	-	-	-	96 (7)	9.31	6.46	105 (6)	7.73	5.68

Analyte	Spiking level 1			Spiking level 2			Spiking level 3			Spiking level 7			Spiking level 8		
	R_E (RSD) [%]	RSDr [%]	RSD R [%]	R_E (RSD) [%]	RSDr [%]	RSD R [%]	R_E (RSD) [%]	RSDr [%]	RSD R [%]	R_E (RSD) [%]	RSDr [%]	RSD R [%]	R_E (RSD) [%]	RSDr [%]	RSD R [%]
phorate-sulfone	88 (6)	7.09	12.1	102 (6)	8.37	9.19	100 (8)	6.76	7.81	95 (6)	7.09	5.83	104 (4)	6.76	5.02
phorate-sulfoxide	106 (7)	8.00	11.0	104 (5)	6.02	8.46	98 (6)	6.00	8.04	96 (4)	6.70	5.00	97 (3)	5.25	7.59
phosmet	-	-	-	-	-	-	106 (3)	7.14	8.41	93 (5)	6.70	7.52	100 (2)	3.59	10.0
phosphamidon	98 (3)	7.49	12.2	114 (5)	6.14	13.6	95 (6)	7.15	6.65	94 (5)	5.89	4.75	104 (5)	6.06	6.66
phoxim	103 (7)	10.4	10.7	119 (5)	7.85	13.0	108 (6)	7.80	10.8	102 (5)	5.83	5.77	106 (3)	4.36	5.41
picoxystrobin	98 (5)	8.05	6.80	112 (3)	8.60	3.98	114 (5)	8.05	13.6	92 (4)	5.34	4.81	101 (4)	3.59	4.25
piperonyl butoxide	103 (9)	15.8	13.2	97 (6)	14.6	7.52	106 (6)	11.6	7.83	105 (6)	6.71	5.36	102 (3)	4.00	3.26
pirimicarb	93 (5)	5.81	4.86	106 (4)	10.0	5.08	102 (3)	7.51	6.93	104 (6)	4.31	5.32	103 (2)	2.99	3.72
pirimicarb-desmethyl	105 (7)	8.55	7.90	100 (3)	9.61	5.71	105 (3)	6.12	7.09	98 (4)	4.36	3.23	96 (1)	2.92	4.21
pirimicarb-desmethylformamido	-	-	-	-	-	-	-	-	-	100 (5)	6.33	6.01	107 (5)	9.10	9.41
primisulfuron-methyl	91 (14)	11.9	12.8	108 (2)	12.4	13.8	87 (10)	11.3	11.1	101 (7)	5.99	6.67	93 (8)	5.50	8.12
prochloraz	107 (2)	6.41	8.31	104 (4)	6.41	5.67	116 (5)	6.46	13.3	97 (4)	6.12	3.82	101 (3)	3.26	3.61
promecarb	97 (4)	10.5	13.1	110 (6)	14.2	9.76	98 (6)	9.39	14.4	147 (9)	6.58	7.96	159 (3)	5.07	4.11
prometon	113 (10)	8.10	9.74	97 (9)	12.5	9.30	103 (7)	10.4	9.35	98 (4)	4.98	3.92	95 (4)	4.47	7.29
prometryn	102 (5)	7.86	7.19	104 (5)	10.5	7.29	103 (7)	7.14	7.96	104 (4)	4.53	5.99	99 (2)	4.05	2.01
propamocarb	105 (14)	12.6	12.2	101 (11)	9.79	9.88	101 (17)	12.2	15.3	88 (10)	7.87	7.17	101 (2)	6.05	3.59
propargite	98 (3)	10.7	2.66	101 (4)	10.8	4.79	98 (7)	10.5	6.03	100 (3)	6.85	4.64	98 (3)	3.76	4.21
propazine	92 (6)	7.89	9.62	98 (6)	8.18	4.35	101 (8)	6.80	9.68	90 (2)	4.76	5.93	100 (2)	3.42	7.03
propham	-	-	-	-	-	-	-	-	-	97 (5)	6.29	6.45	101 (5)	8.26	8.83
propiconazole	107 (6)	6.88	9.84	110 (5)	6.20	7.90	98 (7)	6.44	6.50	95 (3)	4.75	4.09	99 (3)	3.60	3.39
propoxur	107 (3)	9.05	27.4	104 (2)	9.73	16.8	108 (4)	9.13	20.1	188 (6)	6.93	8.90	178 (1)	1.98	8.64
propoxycarbazone	-	-	-	-	-	-	107 (17)	14.7	16.6	90 (4)	9.05	8.34	116 (8)	7.35	12.4
proquinazid	98 (4)	7.70	5.65	113 (2)	6.35	9.20	108 (4)	6.83	7.62	98 (5)	6.55	3.87	98 (2)	2.45	2.90
prosulfocarb	97 (10)	8.07	7.07	90 (4)	8.18	7.28	105 (3)	6.54	6.81	103 (5)	6.44	5.89	99 (3)	3.31	4.11
prosulfuron	92 (2)	5.98	7.69	101 (6)	5.71	6.80	103 (7)	7.94	8.87	99 (4)	5.18	6.13	98 (5)	5.35	4.42

Analyte	Spiking level 1			Spiking level 2			Spiking level 3			Spiking level 7			Spiking level 8		
	R_E (RSD) [%]	RSDr [%]	RSD R [%]	R_E (RSD) [%]	RSDr [%]	RSD R [%]	R_E (RSD) [%]	RSDr [%]	RSD R [%]	R_E (RSD) [%]	RSDr [%]	RSD R [%]	R_E (RSD) [%]	RSDr [%]	RSD R [%]
pymetrozine	-	-	-	-	-	-	-	-	-	106 (23)	18.2	26.5	78 (15)	11.6	36.8
pyraclostrobin	94 (3)	6.46	4.52	102 (6)	8.11	6.91	108 (4)	7.20	8.70	93 (7)	5.64	5.60	97 (3)	3.74	3.88
pyraflufen-ethyl	105 (7)	7.65	10.3	102 (9)	8.64	8.70	115 (4)	5.22	9.40	97 (5)	6.75	5.51	107 (4)	4.11	5.96
pyrethrin I	-	-	-	-	-	-	105 (3)	9.87	6.40	107 (5)	5.72	6.38	104 (4)	3.74	4.82
pyrethrin II	-	-	-	-	-	-	122 (11)	28.2	17.0	102 (9)	11.0	9.77	108 (2)	5.35	5.94
pyridate	n.a.	n.a.	n.a.	n.a.	n.a.	n.a.	n.a.	n.a.	n.a.	n.a.	n.a.	n.a.	n.a.	n.a.	n.a.
pyridate-metabol (6-chloro-3-phenylpyridazin-4-ol)	101 (7)	9.85	9.65	99 (7)	7.58	4.56	95 (1)	4.46	5.61	92 (5)	6.86	5.28	95 (3)	4.36	3.65
pyrimethanil	105 (10)	9.98	9.90	102 (6)	7.99	8.84	104 (4)	5.31	6.10	97 (4)	5.80	5.25	100 (4)	5.34	5.06
pyrimidifen	93 (13)	12.6	8.89	104 (6)	12.2	5.28	104 (7)	10.1	8.10	107 (3)	5.76	9.54	94 (5)	3.98	5.39
pyriproxyfen	106 (1)	7.14	6.78	100 (3)	6.24	6.67	103 (4)	7.39	6.77	97 (5)	3.65	3.32	103 (1)	2.09	2.85
quizalofop-ethyl	98 (3)	7.44	5.45	103 (4)	9.07	4.65	97 (6)	8.32	6.55	95 (4)	8.10	4.93	96 (2)	4.29	3.56
rabenzazole	102 (8)	8.39	11.9	99 (13)	11.3	13.3	101 (7)	8.96	9.17	92 (6)	6.69	5.90	100 (1)	5.97	7.39
rimsulfuron	101 (4)	5.06	12.3	95 (5)	6.70	11.5	98 (8)	6.56	14.0	88 (8)	6.93	5.80	105 (6)	6.16	10.8
rotenone	94 (4)	9.26	5.70	114 (7)	9.15	7.48	109 (3)	9.54	7.14	96 (6)	7.20	5.02	96 (2)	3.52	2.75
sebuthylazine	103 (5)	5.05	10.1	108 (7)	5.71	7.29	108 (5)	5.38	9.37	103 (3)	6.46	4.57	97 (2)	4.66	2.58
sethoxydim	118 (4)	8.10	14.9	108 (6)	11.9	7.41	118 (3)	11.9	8.25	106 (4)	5.04	7.17	100 (1)	3.29	3.98
silafluofen	100 (3)	8.48	4.37	101 (2)	7.94	3.04	107 (4)	5.90	8.29	98 (3)	4.24	3.74	96 (1)	3.33	4.67
simazine	102 (8)	5.98	13.8	92 (7)	8.90	6.61	115 (2)	9.18	12.7	97 (5)	6.07	4.21	100 (3)	4.42	4.52
simazine-desethyl	-	-	-	-	-	-	-	-	-	85 (3)	5.20	5.46	94 (3)	2.61	3.10
simeconazol	-	-	-	-	-	-	-	-	-	102 (7)	6.75	5.79	97 (4)	3.34	5.02
spinosyn A	-	-	-	-	-	-	-	-	-	100 (5)	9.15	3.83	100 (4)	18.3	6.96
spinosyn D	-	-	-	-	-	-	-	-	-	99 (4)	10.9	3.77	98 (3)	20.0	6.36
spirodiclofen	221 (6)	12.8	36.3	240 (4)	11.6	31.6	208 (5)	13.5	34.2	131 (7)	10.6	16.1	141 (7)	6.48	14.0
spiromesifen	-	-	-	-	-	-	107 (5)	20.4	9.52	103 (4)	11.4	7.64	111 (7)	11.4	9.39
spirotetramat	105 (4)	15.1	8.31	114 (9)	10.4	10.3	115 (7)	10.5	13.2	99 (6)	6.82	5.51	97 (4)	5.45	4.76

Analyte	Spiking level 1			Spiking level 2			Spiking level 3			Spiking level 7			Spiking level 8		
	R_E (RSD) [%]	RSDr [%]	RSD R [%]	R_E (RSD) [%]	RSDr [%]	RSD R [%]	R_E (RSD) [%]	RSDr [%]	RSD R [%]	R_E (RSD) [%]	RSDr [%]	RSD R [%]	R_E (RSD) [%]	RSDr [%]	RSD R [%]
spiroxamine	108 (4)	5.95	9.77	106 (7)	5.35	7.34	108 (5)	6.56	10.7	105 (4)	4.85	5.69	105 (4)	8.50	9.34
tebuconazole	-	-	-	-	-	-	105 (3)	5.71	8.01	100 (6)	6.79	4.71	100 (3)	2.99	2.94
tebufenozide	103 (10)	11.2	9.24	107 (5)	10.6	6.49	100 (4)	10.4	6.98	97 (4)	7.00	4.53	106 (5)	5.08	5.29
tebufenpyrad	104 (6)	10.2	13.1	109 (8)	10.9	9.32	97 (8)	12.0	7.13	103 (7)	9.47	9.20	100 (5)	5.56	4.31
teflubenzuron	-	-	-	-	-	-	104 (4)	15.8	10.9	101 (4)	13.7	14.1	99 (8)	11.5	9.41
tetraethyl diphosphate	96 (6)	6.21	8.20	116 (5)	7.03	12.5	115 (5)	4.67	10.1	102 (3)	4.90	5.99	110 (2)	3.53	4.64
terbacil	-	-	-	-	-	-	121 (8)	15.7	12.2	97 (5)	6.73	5.37	97 (3)	2.73	5.04
terbufos	-	-	-	-	-	-	105 (5)	6.85	9.19	98 (3)	5.68	4.05	99 (3)	3.43	3.13
terbufos-sulfone	110 (10)	7.05	13.0	107 (3)	7.15	6.92	99 (5)	4.78	5.13	98 (3)	6.53	5.86	103 (2)	6.93	5.38
terbufos-sulfoxide	103 (3)	6.50	7.31	106 (6)	6.49	5.58	110 (5)	6.51	8.72	103 (5)	5.63	5.39	101 (4)	3.88	4.51
terbuthylazine	98 (9)	8.84	9.95	102 (4)	6.21	7.86	95 (4)	6.56	5.87	103 (3)	5.51	3.94	104 (4)	3.95	6.23
terbuthylazine-desethyl	96 (6)	4.93	6.16	97 (2)	3.70	6.01	113 (4)	4.75	12.7	98 (5)	5.65	3.57	99 (2)	3.01	3.52
terbutryn	95 (6)	7.29	4.69	100 (3)	8.33	6.29	102 (5)	8.86	5.52	100 (5)	5.99	4.24	98 (4)	3.24	3.57
tetraconazole	105 (7)	8.33	10.6	101 (8)	11.3	8.42	111 (3)	5.62	13.1	99 (6)	6.02	5.78	105 (5)	4.17	9.87
thiabendazole	93 (6)	5.84	9.34	101 (2)	3.60	3.32	103 (4)	5.27	8.56	94 (2)	5.60	4.08	103 (6)	5.14	7.17
thiabendazole-5-hydroxy	85 (7)	6.45	6.50	100 (5)	3.78	10.2	95 (7)	5.29	11.9	106 (4)	4.10	6.13	102 (3)	5.53	3.55
thiacloprid	98 (4)	5.74	4.76	102 (7)	5.17	7.07	103 (8)	5.93	7.40	88 (4)	6.52	5.50	95 (2)	2.62	3.89
thiametoxam	90 (9)	13.2	7.22	97 (5)	13.8	6.70	91 (4)	13.8	7.73	106 (6)	5.91	8.77	95 (5)	3.57	5.53
thiazafluron	-	-	-	-	-	-	-	-	-	94 (3)	5.17	3.44	105 (3)	2.41	4.86
thifensulfuron-methyl	96 (6)	9.11	9.35	96 (7)	5.58	8.50	105 (5)	6.66	8.38	100 (4)	5.74	7.08	88 (7)	5.91	8.75
thiodicarb	-	-	-	-	-	-	-	-	-	852 (5)	6.11	10.4	891 (4)	4.17	11.5
thiofanox	-	-	-	-	-	-	-	-	-	109 (9)	23.3	14.7	92 (6)	29.3	15.5
thiofanox-sulfone	-	-	-	-	-	-	86 (9)	10.5	11.6	104 (6)	5.68	6.53	107 (4)	3.86	4.29
thiofanox-sulfoxide	-	-	-	-	-	-	-	-	-	99 (5)	6.10	5.74	98 (3)	4.21	4.15
thiometon	-	-	-	-	-	-	-	-	-	94 (6)	9.83	9.92	94 (3)	5.52	12.1

Analyte	Spiking level 1			Spiking level 2			Spiking level 3			Spiking level 7			Spiking level 8		
	R_E (RSD) [%]	RSDr [%]	RSD R [%]	R_E (RSD) [%]	RSDr [%]	RSD R [%]	R_E (RSD) [%]	RSDr [%]	RSD R [%]	R_E (RSD) [%]	RSDr [%]	RSD R [%]	R_E (RSD) [%]	RSDr [%]	RSD R [%]
thionazin	93 (9)	8.02	8.58	91 (8)	8.70	8.58	94 (6)	8.67	11.0	94 (4)	7.66	4.46	97 (2)	3.10	4.04
thiophanate-ethyl	n.a.	n.a.	n.a.	n.a.	n.a.	n.a.	n.a.	n.a.	n.a.	n.a.	n.a.	n.a.	n.a.	n.a.	n.a.
thiophanate-methyl	n.a.	n.a.	n.a.	n.a.	n.a.	n.a.	n.a.	n.a.	n.a.	n.a.	n.a.	n.a.	n.a.	n.a.	n.a.
tiocarbazil	-	-	-	-	-	-	-	-	-	99 (2)	3.16	6.11	103 (4)	4.11	14.2
triadimefon	98 (7)	7.13	7.36	103 (5)	9.84	6.84	113 (6)	7.23	9.67	97 (5)	5.74	4.65	101 (3)	2.77	6.14
triadimenol	90 (3)	7.65	6.19	101 (7)	6.44	6.61	89 (5)	5.61	5.48	94 (7)	6.58	5.77	102 (3)	3.06	4.64
triamiphos	96 (9)	8.55	11.0	105 (4)	8.88	8.28	113 (8)	7.81	15.8	101 (5)	6.63	6.93	97 (2)	3.96	5.03
triasulfuron	90 (5)	7.56	7.57	98 (6)	4.73	6.64	106 (7)	8.14	7.90	97 (4)	4.38	3.92	93 (3)	3.61	5.50
triazamate	-	-	-	-	-	-	102 (7)	7.27	11.1	114 (6)	6.32	7.97	118 (4)	5.42	5.94
triazophos	92 (12)	12.0	11.1	119 (11)	9.00	11.7	114 (4)	5.71	13.6	100 (5)	6.61	5.45	115 (5)	5.61	8.33
tribenuron-methyl	88 (3)	5.84	11.0	94 (3)	4.39	7.49	84 (4)	4.10	6.61	85 (5)	6.21	4.22	85 (3)	7.84	7.13
trichlorfon	n.a.	n.a.	n.a.	n.a.	n.a.	n.a.	n.a.	n.a.	n.a.	n.a.	n.a.	n.a.	n.a.	n.a.	n.a.
tricyclazole	88 (7)	8.23	7.22	98 (2)	6.75	3.93	99 (3)	4.30	4.77	96 (3)	5.37	3.34	101 (2)	5.17	2.46
tridemorph	-	-	-	-	-	-	-	-	-	102 (9)	8.32	6.13	92 (5)	4.86	8.54
trietazine	92 (6)	8.43	6.73	109 (3)	5.69	9.57	107 (5)	6.90	7.71	96 (5)	7.19	6.59	95 (5)	6.44	3.78
trifloxystrobin	98 (4)	5.97	5.37	108 (6)	6.17	6.86	112 (4)	4.85	7.41	95 (6)	6.71	4.51	101 (2)	3.43	3.43
trifloxysulfuron	87 (8)	15.6	8.02	103 (9)	10.3	12.4	89 (8)	10.9	9.61	100 (8)	10.5	6.95	90 (3)	4.86	6.91
triflumizole	107 (5)	7.35	6.54	104 (2)	7.38	7.43	129 (4)	10.5	14.1	99 (2)	5.04	3.81	98 (2)	3.90	2.68
triflumizole-metabole FM-6-1	111 (11)	8.57	9.76	107 (6)	6.90	6.84	108 (3)	6.36	7.49	94 (4)	5.74	4.66	98 (3)	5.95	4.14
triflumuron	89 (13)	13.1	8.84	116 (11)	9.58	11.0	117 (6)	10.9	12.9	96 (6)	8.50	7.01	112 (5)	5.00	7.02
triflusulfuron-methyl	103 (7)	5.83	9.00	95 (7)	5.59	6.75	104 (3)	3.18	7.44	96 (5)	5.54	4.74	98 (1)	3.30	1.90
triforine	102 (8)	13.6	10.9	113 (9)	8.90	14.1	109 (5)	8.81	10.4	93 (6)	6.50	4.55	108 (2)	4.88	5.66
triticonazole	96 (12)	7.83	14.1	103 (13)	11.9	11.5	95 (6)	7.86	7.10	101 (4)	6.01	5.29	95 (4)	4.08	6.19
uniconazole	-	-	-	-	-	-	-	-	-	100 (3)	5.75	6.07	102 (4)	4.34	4.19
vamidothion	101 (6)	6.99	8.67	103 (6)	6.74	9.24	106 (7)	6.09	9.86	98 (1)	3.48	4.59	100 (3)	3.58	2.49
vamidothion-sulfone	153 (5)	5.28	10.9	158 (5)	5.51	10.1	152 (5)	4.43	12.0	129 (3)	6.05	26.5	129 (3)	5.03	22.4

Analyte	Spiking level 1			Spiking level 2			Spiking level 3			Spiking level 7			Spiking level 8		
	R_E (RSD) [%]	RSDr [%]	RSD R [%]	R_E (RSD) [%]	RSDr [%]	RSD R [%]	R_E (RSD) [%]	RSDr [%]	RSD R [%]	R_E (RSD) [%]	RSDr [%]	RSD R [%]	R_E (RSD) [%]	RSDr [%]	RSD R [%]
vamidothion-sulfoxide	91 (4)	6.44	2.99	106 (3)	4.75	5.29	98 (5)	4.61	4.85	92 (4)	4.26	4.77	92 (3)	2.76	7.03
zoxamide	148 (5)	11.0	9.35	131 (6)	11.2	11.2	140 (3)	8.16	4.90	112 (3)	5.56	4.34	114 (2)	4.33	6.83

Table S8 Matrix Suppression/Enhancement (MSE) Effects for Pesticides at Spiking Level no. 7 in Wheat.

Analyte	MSE [%]	Analyte	MSE [%]
3,4,5-trimethacarb	3	imibenconazole	-7
acephate	75	imidacloprid	-10
acetamiprid	3	indoxacarb	4
acetochlor	-9	iodosulfuron-methyl	6
alachlor	-8	iprovalicarb	0
aldicarb	0	isoprocarb	-2
aldicarb-sulfoxide	25	isoprothiolane	-15
aldoxycarb	-3	isoproturon	-3
ametoctradin	-7	isoxaben	-9
ametryn	-1	isoxaflutole	n.a.
amidosulfuron	-16	isoxathion	-11
aminocarb	-15	lenacil	5
aminopyralid	14	linuron	-5
amitraz	-78	lufenuron	-19
amitraz-amide	13	malaoxon	5
amitraz-amidin	-85	malathion	-8
ancymidol	-6	mandipropamid	3
atrazine	-6	mepanipyrim	-6
atrazine-desethyl-desisopropyl	-15	metalaxyl	-4
avermectin B1a	-3	metamitron	-15
avermectin B1b	n.a.	metazachlor	0
azaconazole	-9	metconazole	0
azamethiphos	-5	methabenzthiazuron	-4
aziprotryne	-3	methacrifos	-8
azoxystrobin	-5	methamidophos	19
benalaxyl	-3	methidathion	-17
bendiocarb	11	methiocarb	-2
benfuracarb	-68	methiocarb-sulfone	52
benodanil	1	methiocarb-sulfoxide	24
benomyl	n.a.	metholachlor	-2
bensulfuron-methyl	8	methomyl	-5
benthiavalicarb-isopropyl	0	methoprotryn	-1
bitertanol	11	methoxyfenozide	0
boscalid	-17	metobromuron	-9
bromacil	-5	metolcarb	-1
bromuconazole	-9	metoxuron	-3
bupirimate	-3	metrafenone	-26
buprofezin	-51	metribuzin	-3
butachlor	-39	metsulfuron-methyl	0
butocarboxim	-4	molinate	1
butocarboxim-sulfoxide	38	monocrotophos	2
butoxycarboxim	1	monolinuron	-5
buturon	-6	monuron	7
cadusafos	-10	napropamide	-5

Analyte	MSE [%]	Analyte	MSE [%]
carbaryl	1	neburon	-12
carbendazim	-9	nicosulfuron	5
carbofuran	16	novaluron	0
carbofuran-3-hydroxy	7	nuarimol	-9
carbosulfan	-73	ofurace	-8
carboxin	-7	omethoate	34
chlorantraniliprole	3	orbencarb	-9
chlorbromuron	-3	oxadixyl	-14
chlorfluazuron	2	oxamyl	0
chloridazon	-6	oxamyl-oxime	2
chloroxuron	9	oxydemeton-methyl	1
chlorpropham	9	paclobutrazol	-4
chlorsulfuron	-7	paraoxon-ethyl	-2
chlortoluron	0	paraoxon-methyl	-7
cinidon-ethyl	-9	penconazole	-19
cinosulfuron	-3	pencycuron	-3
clethodim	-7	pendimethalin	-2
clodinafop-propargyl	-3	pentanochlor	-5
clofentezine	-24	phenmedipham	n.a.
clomazone	-4	phorate	-4
clothianidin	0	phorate-sulfone	-6
cyanazine	-5	phorate-sulfoxide	-8
cyazofamid	-6	phosmet	1
cymoxanil	4	phosphamidon	2
cyproconazole	-10	phoxim	-18
cyprodinil	-11	picoxystrobin	0
cyprofuram	-1	piperonyl butoxide	-6
cyromazine	-26	pirimicarb	-2
daminozide	-52	pirimicarb-desmethyl	5
demeton	-12	pirimicarb-desmethylformamido	3
demeton-S-methyl	0	primisulfuron-Methyl	6
demeton-S-methyl-sulfone	0	prochloraz	-13
desmedipham	n.a.	promecarb	9
desmetryn	-6	prometon	2
diazinon	-11	prometryn	0
dichlorvos	3	propamocarb	-50
diclobutrazol	-37	propargite	-32
diethofencarb	3	propazine	-1
diethyltoluamide	-4	propham	-1
difenoconazole	-13	propiconazole	-9
difenoxuron	-3	propoxur	8
diflubenzuron	-6	propoxycarbazone	-13
diflufenican	-15	proquinazid	-9
dimefox	-4	prosulfocarb	-18
dimefuron	3	prosulfuron	-1
dimethenamid	-11	pymetrozine	1536
dimethoate	-9	pyraclostrobin	-35
dimethomorph	6	pyraflufen-ethyl	-3

Analyte	MSE [%]	Analyte	MSE [%]
dimetilan	0	pyrethrin I	-4
dimoxystrobin	-2	pyrethrin II	13
dinotefuran	-5	pyridate	n.a.
disulfoton	-7	pyridate-metabol (6-chloro-3-phenylpyridazin-4-ol)	-19
disulfoton-sulfone	1	pyrimethanil	0
disulfoton-sulfoxide	-9	pyrimidifen	9
diuron	-4	pyriproxyfen	-18
dodine	n.a.	quizalofop-ethyl	-32
emamectin B1a	425	rabenzazole	3
emamectin B1b	461	rimsulfuron	-1
epoxiconazole	-9	rotenone	12
ethiofencarb	18	sebuthylazine	-9
ethiofencarb-sulfone	32	sethoxydim	-6
ethiofencarb-sulfoxide	18	silafluofen	-2
ethiprole	-4	simazine	1
ethofumesate	-3	simazine-desethyl	-17
ethofumesate-2-keto	-1	simeconazol	-4
ethoprofos	0	spinosyn A	318
etofenprox	-33	spinosyn D	286
etoxazole	-30	spirodiclofen	82
famoxadon	-12	spiromesifen	-38
fenamidone	-15	spirotetramat	14
fenamiphos	-7	spiroxamine	412
fenamiphos-sulfone	1	tebuconazole	0
fenamiphos-sulfoxide	-3	tebufenozide	-6
fenarimol	-6	tebufenpyrad	-16
fenazaquin	-16	teflubenzuron	-6
fenbuconazole	-4	tetraethyl diphosphate	-1
fenhexamid	-10	terbacil	1
fenobucarb	3	terbufos	-23
fenoxaprop-P-ethyl	-20	terbufos-sulfone	-8
fenoxycarb	2	terbufos-sulfoxide	-6
fenpiclonil	-3	terbuthylazine	-7
fenpropidin	514	terbuthylazine-desethyl	-10
fenpropimorph	-6	terbutryn	-6
fenpyroximate	-3	tetraconazole	-28
fensulfothion	-2	thiabendazole	-4
fensulfothion-PO-sulfone	-1	thiabendazole-5-hydroxy	693
fensulfothion-PO-sulfoxide	-3	thiacloprid	-1
fensulfothion-PS-sulfone	-3	thiametoxam	-6
fenthion	-12	thiazafluron	-2
fenthion-oxon	-5	thifensulfuron-methyl	29
fenthion-PO-sulfone	-11	thiodicarb	16
fenthion-PO-sulfoxide	-2	thiofanox	-4
fenthion-PS-sulfone	-10	thiofanox-sulfone	12
fenthion-PS-sulfoxide	5	thiofanox-sulfoxide	-2
fenuron	-3	thiometon	-20

Analyte	MSE [%]	Analyte	MSE [%]
flazasulfuron	-4	thionazin	-2
flonicamid	-12	thiophanate-ethyl	n.a.
florasulam	0	thiophanate-methyl	n.a.
fluazifop-P-butyl	-35	tiocarbazil	-72
fluazuron	-23	triadimefon	1
flucycloxuron	6	triadimenol	-3
fludioxonil	2	triamiphos	5
flufenacet	-10	triasulfuron	0
flufenoxuron	5	triazamate	-9
fluometuron	-6	triazophos	-5
fluopicolide	-28	tribenuron-methyl	-22
flurochloridone	-5	trichlorfon	n.a.
flurprimidol	-6	tricyclazole	0
flusilazole	-6	tridemorph	-37
flutriafol	-10	trietazine	-4
formetanate	-59	trifloxystrobin	-53
fosthiazate	-3	trifloxysulfuron	-14
fuberidazole	-5	triflumizole	-5
furathiocarb	-9	triflumizole-metabole FM-6-1	-8
halofenozide	-6	triflumuron	-4
haloxyfop	33	triflusulfuron-methyl	-7
haloxyfop-2-ethoxyethyl	-24	triforine	2
haloxyfop-methyl	-5	triticonazole	-21
hexaconazole	-6	uniconazole	-17
hexaflumuron	-18	vamidothion	-4
hexazinone	-2	vamidothion-sulfone	40
hexythiazox	2	vamidothion-sulfoxide	187
imazalil	-11	zoxamide	29

Table S9 Recoveries (R_E, n = 6), Repeatabilities (RSDr), and Within-Laboratory Reproducibilities (RSDR) for Mycotoxins, Plant Growth Regulators, and Tropane Alkaloids at 4 Spiking Levels in Wheat.

Analyte	Spiking level 2			Spiking level 3			Spiking level 7			Spiking level 8		
	R_E (RSD) [%]	RSDr [%]	RSDR [%]	R_E (RSD) [%]	RSDr [%]	RSDR [%]	R_E (RSD) [%]	RSDr [%]	RSDR [%]	R_E (RSD) [%]	RSDr [%]	RSDR [%]
aflatoxin B1	106 (3)	7.10	13.3	115 (6)	5.88	11.0	97 (3)	5.17	3.65	93 (2)	5.70	2.83
aflatoxin B2	109 (2)	9.98	8.97	98 (3)	4.50	5.56	98 (6)	5.99	5.17	101 (3)	4.80	5.55
aflatoxin G1	113 (9)	10.6	9.75	110 (8)	6.10	5.77	107 (3)	5.65	3.50	105 (4)	8.46	3.85
aflatoxin G2	95 (10)	9.61	10.3	100 (6)	6.05	10.6	97 (4)	5.08	4.41	96 (3)	4.43	4.09
altenuene	108 (12)	10.6	14.9	96 (10)	10.21	15.0	95 (6)	7.05	6.34	97 (2)	3.28	6.35
alternariol	99 (7)	7.41	9.16	97 (9)	5.94	11.2	96 (3)	6.19	4.25	95 (2)	6.80	5.98
alternariol monomethyl ether	101 (7)	6.07	6.92	105 (3)	4.19	7.50	94 (4)	5.77	3.48	101 (2)	5.84	3.22
citrinin	63 (12)	9.72	16.7	54 (6)	9.41	12.12	60 (7)	7.66	7.80	62 (4)	6.98	10.0
deoxynivalenol	102 (6)	12.3	11.2	97 (10)	12.6	11.9	93 (6)	9.96	6.36	101 (3)	9.79	7.13
deoxynivalenol-3-glucoside	89 (4)	6.87	8.67	91 (2)	7.69	5.31	83 (2)	5.75	2.83	-	-	-
15-acetyldeoxynivalenol	104 (9)	7.96	12.4	93 (8)	7.41	6.31	93 (6)	6.28	8.68	106 (2)	2.53	5.92
3-acetyldeoxynivalenol	109 (8)	7.91	14.6	113 (5)	11.9	17.0	104 (7)	8.59	9.25	99 (4)	6.42	6.58
diacetoxyscirpenol	94 (10)	7.59	8.26	92 (5)	6.77	5.63	98 (6)	5.49	4.61	97 (4)	5.04	4.16
fumonisin B1	82 (27)	17.5	93.0	74 (6)	9.72	69.6	290 (6)	14.0	72.6	281 (7)	19.1	271
hydrolyzed fumonisin B1	95 (7)	8.95	13.8	92 (16)	13.3	12.6	96 (11)	10.7	17.0	88 (11)	12.8	12.4
fumonisin B2	104 (8)	6.11	77.1	120 (6)	7.01	74.9	300 (4)	10.3	61.4	349 (7)	18.1	206
hydrolyzed fumonisin B2	97 (3)	8.67	5.28	99 (1)	6.78	7.25	107 (4)	8.77	5.69	99 (3)	4.45	5.47
fumonisin B3	108 (7)	7.08	68.4	120 (11)	8.74	63.9	283 (4)	14.2	60.4	312 (3)	16.8	194
fusarenon X	104 (16)	10.7	11.7	102 (9)	6.96	10.2	90 (2)	4.31	4.00	98 (2)	3.28	5.45
HT-2 toxin	96 (3)	5.88	8.34	95 (11)	7.93	9.69	99 (6)	5.02	5.66	105 (4)	4.25	5.49
neosolaniol	90 (11)	11.6	12.5	112 (9)	9.22	13.2	97 (5)	5.93	7.00	104 (3)	3.94	6.26
nivalenol	93 (4)	6.79	12.9	90 (11)	10.1	10.6	81 (12)	9.50	13.8	98 (6)	6.71	10.0

Analyte	Spiking level 2			Spiking level 3			Spiking level 7			Spiking level 8		
	R_E (RSD) [%]	RSDr [%]	RSDR [%]	R_E (RSD) [%]	RSDr [%]	RSDR [%]	R_E (RSD) [%]	RSDr [%]	RSDR [%]	R_E (RSD) [%]	RSDr [%]	RSDR [%]
ochratoxin A	103 (9)	7.17	9.98	111 (4)	7.14	5.69	94 (2)	5.73	3.97	104 (2)	4.09	4.23
sterigmatocystin	104 (5)	5.95	8.83	107 (3)	12.0	7.25	97 (4)	5.99	4.84	99 (4)	4.23	4.07
T-2 toxin	81 (7)	9.62	13.9	101 (9)	8.17	8.91	93 (6)	5.48	6.77	101 (1)	3.32	5.12
tentoxin	109 (3)	9.14	7.45	104 (3)	8.26	8.65	100 (4)	5.27	5.04	95 (6)	5.03	6.48
zearalenone	104 (10)	9.71	7.14	106 (3)	2.91	6.14	97 (1)	4.48	4.02	103 (3)	5.00	3.56
zearalenone-14-glucoside	94 (9)	6.66	6.35	89 (7)	8.02	6.06	94 (3)	5.90	6.86	93 (4)	3.74	8.27
zearalenone-14-sulfate	84 (3)	4.88	6.94	96 (2)	3.54	9.43	85 (6)	5.30	4.66	85 (5)	6.79	4.04
zearalenone-14,16-disulfate	8 (7)	8.70	9.56	8 (11)	14.1	14.1	7 (4)	11.3	7.60	7 (1)	18.3	9.06
α-zearalenol	110 (5)	4.14	7.11	108 (4)	4.01	5.23	105 (4)	5.42	3.49	108 (1)	3.67	3.71
α-zearalenol-14-glucoside	105 (12)	7.68	10.8	95 (14)	14.7	13.1	92 (13)	13.6	11.5	89 (6)	9.42	11.8
α-zearalenol-14-sulfate	84 (3)	6.61	7.17	87 (1)	2.97	6.52	83 (6)	6.22	6.22	90 (3)	6.03	6.83
β-zearalenol	103 (4)	6.59	6.92	96 (6)	5.77	7.55	102 (5)	5.79	4.68	102 (3)	2.58	4.40
β-zearalenol-14-glucoside	88 (12)	13.4	10.6	93 (17)	13.1	14.0	92 (13)	9.75	10.8	89 (6)	8.50	11.1
β-zearalenol-14-sulfate	77 (4)	8.83	8.80	75 (3)	3.00	7.21	65 (11)	8.00	9.00	88 (5)	5.73	9.03
zearalanone	103 (7)	5.00	6.24	94 (4)	7.30	5.35	99 (2)	3.89	2.84	102 (2)	2.23	2.76
zearalanone-14-glucoside	113 (8)	7.03	12.5	93 (9)	9.32	9.24	95 (4)	7.43	4.80	92 (6)	4.80	10.6
α-zearalanol	99 (2)	3.74	3.37	99 (4)	5.08	8.40	90 (5)	8.30	9.59	97 (5)	6.04	6.80
β-zearalanol	100 (7)	9.22	10.9	103 (6)	14.6	11.0	100 (4)	11.2	7.18	99 (9)	10.7	5.60
chlormequat	101 (4)	6.46	5.03	96 (6)	7.19	7.04	95 (5)	4.97	4.11	95 (2)	2.98	5.71
mepiquat	98 (5)	5.41	4.29	97 (3)	6.16	2.94	93 (3)	4.13	2.55	92 (2)	6.97	3.22
atropine	99 (4)	11.1	6.50	99 (5)	12.0	4.57	105 (6)	7.15	5.86	104 (4)	4.55	4.12
scopolamine	101 (3)	7.99	6.80	98 (7)	15.2	7.62	96 (8)	7.94	6.78	95 (7)	7.73	8.91

Table S10 Overall Validation Results [Recoveries (R_E), Repeatabilities (RSDr), and Within-Laboratory Reproducibilities (RSDR) Assessed at the Respective Limits of Quantification for Mycotoxins, Plant Growth Regulators, Tropane Alkaloids, and Spiking Level no. 3 and 4 for Pesticides; Measurement Uncertainty (MU)].

Analyte	Validation	Analyte	Validation
aflatoxin B1	ok	flucycloxuron	ok
aflatoxin B2	ok	fludioxonil	ok
aflatoxin G1	ok	flufenacet	ok
aflatoxin G2	ok	flufenoxuron	ok
altenuene	ok	fluometuron	ok
alternariol	ok	fluopicolide	ok
alternariol monomethyl ether	ok	flurochloridone	ok
citrinin	R_E	flurprimidol	ok
deoxynivalenol	ok	flusilazole	ok
deoxynivalenol-3-glucoside	ok	flutriafol	ok
15-acetyldeoxynivalenol	ok	formetanate	MU
3-acetyldeoxynivalenol	ok	fosthiazate	ok
diacetoxyscirpenol	ok	fuberidazole	ok
fumonisin B1	RSDR, MU	furathiocarb	R_E, RSDR, MU
hydrolyzed fumonisin B1	ok	halofenozide	ok
fumonisin B2	RSDR, MU	haloxyfop	RSDr
hydrolyzed fumonisin B2	ok	haloxyfop-2-ethoxyethyl	ok
fumonisin B3	RSDR, MU	haloxyfop-methyl	ok
fusarenon X	ok	hexaconazole	ok
HT-2 toxin	ok	hexaflumuron	ok
neosolaniol	ok	hexazinone	ok
nivalenol	ok	hexythiazox	ok
ochratoxin A	ok	imazalil	ok
sterigmatocystin	ok	imibenconazole	ok
T-2 toxin	ok	imidacloprid	ok
tentoxin	ok	indoxacarb	ok
zearalenone	ok	iodosulfuron-methyl	ok
zearalenone-14-glucoside	ok	iprovalicarb	ok
zearalenone-14-sulfate	ok	isoprocarb	ok
zearalenone-14,16-disulfate	R_E	isoprothiolane	ok
α-zearalenol	ok	isoproturon	ok
α-zearalenol-14-glucoside	ok	isoxaben	ok
α-zearalenol-14-sulfate	ok	isoxaflutole	n.a.
β-zearalenol	ok	isoxathion	ok
β-zearalenol-14-glucoside	ok	lenacil	ok
β-zearalenol-14-sulfate	ok	linuron	ok
zearalanone	ok	lufenuron	ok
zearalanone-14-glucoside	ok	malaoxon	ok
α-zearalanol	ok	malathion	ok
β-zearalanol	ok	mandipropamid	ok
chlormequat	ok	mepanipyrim	ok

Analyte	Validation	Analyte	Validation
mepiquat	ok	metalaxyl	ok
atropine	ok	metamitron	ok
scopolamine	ok	metazachlor	ok
3,4,5-trimethacarb	ok	metconazole	ok
acephate	ok	methabenzthiazuron	ok
acetamiprid	ok	methacrifos	ok
acetochlor	ok	methamidophos	ok
alachlor	ok	methidathion	ok
aldicarb	ok	methiocarb	R_E, RSDR, MU
aldicarb-sulfoxide	ok	methiocarb-sulfone	R_E, RSDR, MU
aldoxycarb	ok	methiocarb-sulfoxide	R_E, RSDR, MU
ametoctradin	ok	metholachlor	ok
ametryn	ok	methomyl	ok
amidosulfuron	ok	methoprotryn	ok
aminocarb	ok	methoxyfenozide	ok
aminopyralid	R_E	metobromuron	ok
amitraz	R_E, RSDr, RSDR, MU	metolcarb	R_E
amitraz-amide	ok	metoxuron	ok
amitraz-amidin	RSDr	metrafenone	ok
ancymidol	ok	metribuzin	ok
atrazine	ok	metsulfuron-methyl	MU
atrazine-desethyl-desisopropyl	ok	molinate	ok
avermectin B1ab	RSDr	monocrotophos	ok
avermectin B1bb	n.a.	monolinuron	ok
azaconazole	ok	monuron	ok
azamethiphos	ok	napropamide	ok
aziprotryne	ok	neburon	ok
azoxystrobin	ok	nicosulfuron	ok
benalaxyl	ok	novaluron	ok
bendiocarb	R_E, RSDR, MU	nuarimol	ok
benfuracarb	R_E, RSDr, RSDR, MU	ofurace	ok
benodanil	ok	omethoate	ok
benomyl	n.a.	orbencarb	ok
bensulfuron-methyl	ok	oxadixyl	ok
benthiavalicarb-isopropyl	ok	oxamyl	ok
bitertanol	ok	oxamyl-oxime	ok
boscalid	ok	oxydemeton-methyl	ok
bromacil	R_E, RSDR	paclobutrazol	ok
bromuconazole	ok	paraoxon-ethyl	ok
bupirimate	ok	paraoxon-methyl	ok
buprofezin	ok	penconazole	ok
butachlor	ok	pencycuron	ok
butocarboxim	ok	pendimethalin	ok
butocarboxim-sulfoxide	ok	pentanochlor	ok
butoxycarboxim	ok	phenmedipham	n.a.

Analyte	Validation	Analyte	Validation
buturon	ok	phorate	ok
cadusafos	ok	phorate-sulfone	ok
carbaryl	R_E, RSDR, MU	phorate-sulfoxide	ok
carbendazim	ok	phosmet	ok
carbofuran	MU	phosphamidon	ok
carbofuran-3-hydroxy	RSDR, MU	phoxim	ok
carbosulfan	R_E, RSDr, RSDR, MU	picoxystrobin	ok
carboxin	ok	piperonyl butoxide	ok
chlorantraniliprole	ok	pirimicarb	ok
chlorbromuron	ok	pirimicarb-desmethyl	ok
chlorfluazuron	ok	pirimicarb-desmethylformamido	ok
chloridazon	ok	primisulfuron-Methyl	ok
chloroxuron	ok	prochloraz	ok
chlorpropham	ok	promecarb	ok
chlorsulfuron	ok	prometon	ok
chlortoluron	ok	prometryn	ok
cinidon-ethyl	ok	propamocarb	ok
cinosulfuron	ok	propargite	ok
clethodim	ok	propazine	ok
clodinafop-propargyl	ok	propham	ok
clofentezine	ok	propiconazole	ok
clomazone	ok	propoxur	RSDR, MU
clothianidin	ok	propoxycarbazone	ok
cyanazine	ok	proquinazid	ok
cyazofamid	ok	prosulfocarb	ok
cymoxanil	R_E, RSDR, MU	prosulfuron	ok
cyproconazole	ok	pymetrozine	RSDR, MU
cyprodinil	ok	pyraclostrobin	ok
cyprofuram	ok	pyraflufen-ethyl	ok
cyromazine	ok	pyrethrin I	ok
daminozide	ok	pyrethrin II	R_E, RSDr
demeton	ok	pyridate	n.a.
demeton-S-methyl	ok	pyridate-metabol (6-chloro-3-phenylpyridazin-4-ol)	ok
demeton-S-methyl-sulfone	ok	pyrimethanil	ok
desmedipham	n.a.	pyrimidifen	ok
desmetryn	ok	pyriproxyfen	ok
diazinon	ok	quizalofop-ethyl	ok
dichlorvos	ok	rabenzazole	ok
diclobutrazol	ok	rimsulfuron	ok
diethofencarb	ok	rotenone	ok
diethyltoluamide	ok	sebuthylazine	ok
difenoconazole	ok	sethoxydim	ok
difenoxuron	ok	silafluofen	ok
diflubenzuron	ok	simazine	ok
diflufenican	ok	simazine-desethyl	ok
dimefox	ok	simeconazol	ok

Analyte	Validation	Analyte	Validation
dimefuron	ok	spinosyn Ad	ok
dimethenamid	ok	spinosyn Dd	ok
dimethoate	ok	spirodiclofen	R_E, RSDR, MU
dimethomorph	ok	spiromesifen	RSDr
dimetilan	ok	spirotetramat	ok
dimoxystrobin	ok	spiroxamine	ok
dinotefuran	ok	tebuconazole	ok
disulfoton	ok	tebufenozide	ok
disulfoton-sulfone	ok	tebufenpyrad	ok
disulfoton-sulfoxide	ok	teflubenzuron	ok
diuron	ok	tetraethyl diphosphate	ok
dodine	n.a.	terbacil	R_E
emamectin B1ac	ok	terbufos	ok
emamectin B1bc	R_E, RSDr, RSDR, MU	terbufos-sulfone	ok
epoxiconazole	ok	terbufos-sulfoxide	ok
ethiofencarb	RSDR, MU	terbuthylazine	ok
ethiofencarb-sulfone	R_E, RSDR, MU	terbuthylazine-desethyl	ok
ethiofencarb-sulfoxide	R_E, RSDR, MU	terbutryn	ok
ethiprole	ok	tetraconazole	ok
ethofumesate	ok	thiabendazole	ok
ethofumesate-2-keto	ok	thiabendazole-5-hydroxy	ok
ethoprofos	ok	thiacloprid	ok
etofenprox	ok	thiametoxam	ok
etoxazole	ok	thiazafluron	ok
famoxadon	ok	thifensulfuron-methyl	ok
fenamidone	ok	thiodicarb	R_E
fenamiphos	ok	thiofanox	RSDr
fenamiphos-sulfone	ok	thiofanox-sulfone	ok
fenamiphos-sulfoxide	ok	thiofanox-sulfoxide	ok
fenarimol	ok	thiometon	ok
fenazaquin	ok	thionazin	ok
fenbuconazole	ok	thiophanate-ethyl	n.a.
fenhexamid	ok	thiophanate-methyl	n.a.
fenobucarb	ok	tiocarbazil	ok
fenoxaprop-P-ethyl	ok	triadimefon	ok
fenoxycarb	ok	triadimenol	ok
fenpiclonil	ok	triamiphos	ok
fenpropidin	ok	triasulfuron	ok
fenpropimorph	ok	triazamate	ok
fenpyroximate	ok	triazophos	ok
fensulfothion	ok	tribenuron-methyl	ok
fensulfothion-PO-sulfone	ok	trichlorfon	n.a.
fensulfothion-PO-sulfoxide	ok	tricyclazole	ok
fensulfothion-PS-sulfone	ok	tridemorph	ok
fenthion	ok	trietazine	ok
fenthion-oxon	ok	trifloxystrobin	ok

Analyte	Validation	Analyte	Validation
fenthion-PO-sulfone	ok	trifloxysulfuron	ok
fenthion-PO-sulfoxide	ok	triflumizole	R_E
fenthion-PS-sulfone	ok	triflumizole-metabole FM-6-1	ok
fenthion-PS-sulfoxide	ok	triflumuron	ok
fenuron	ok	triflusulfuron-methyl	ok
flazasulfuron	ok	triforine	ok
flonicamid	ok	triticonazole	ok
florasulam	ok	uniconazole	ok
fluazifop-P-butyl	ok	vamidothion	ok
fluazuron	RSDr	vamidothion-sulfone	R_E
flucycloxuron	ok	vamidothion-sulfoxide	ok
fludioxonil	ok	zoxamide	R_E

Table S11 Detailed Survey Results of Contaminants and Pesticides in Cereal Samples.

No.	Matrix	Concentration [µg/kg]																											
		AOH	AME	DON	D3G	HT-2	NIV	OTA	STG	T-2	TEN	ZEN	CCC	AZS	CBD	CYA	DEET	DFB	EPO	IMD	MAL	PBO	PRO	PYS	TBA	TFB	THO	TCA	TFM
1	wheat																												
2	wheat												6.7																
3	barley					59				19															27				
4	wheat										5.5																		
5	barley																					11							
6	wheat												47																
7	barley						453																						
8	wheat												213						5.7										
9	wheat													7.4											32				
10	oats	46	5.7			52		5.1	> LIN		19	21																	
11	rye										24		34																
12	oats																												
13	rye	42	2.9	171							8.2																		
14	spelt	> LIN	> LIN								11	25																	
15	wheat	1.1																											
16	wheat													9.9											42				
17	rye										12						27												
18	wheat										18																		
19	rye																												
20	rye	7	13	153					3.4			38										6.2			20			20	
21	wheat	1.1		640	60																44	461							
22	barley	8.2		672	111						15				41								8.6	5.5	20				18
23	barley	16	3.3	1034	179						13	17			17							5.7	11		11				16
24	wheat		4.7										59									32			17				
25	barley	13	3.2		226						15	22			44			7.6				11	8.8	5.8	13				18
26	barley	7.9	2.2	808	252						15				42			7.7	5.7			10	8.2		19				24

No.	Matrix	Concentration [µg/kg]																											
		AOH	AME	DON	D3G	HT-2	NIV	OTA	STG	T-2	TEN	ZEN	CCC	AZS	CBD	CYA	DEET	DFB	EPO	IMD	MAL	PBO	PRO	PYS	TBA	TFB	THO	TCA	TFM
27	wheat										5.6																		
28	wheat																												
29	wheat																												
30	oats												23																
31	wheat										5.6																		
32	rice								1.1					5.4		89			16	31			22			11	7.5		
33	oats	6.5	1.1				521			17	21																		
34	wheat							10	18																				
35	wheat				259						9.9																		
36	wheat		1.3		114						27											6.2							

Abbreviations: AOH – alternariol; AME – alternariol monomethyl ether; DON – deoxynivalenol; D3G – deoxynivalenol-3-glucoside; HT-2 – HT-2 toxin; NIV – nivalenol; OTA – ochratoxin A; STG – sterigmatocystin; T-2 – T-2 toxin; TEN – tentoxin; ZEN – zearalenone; CCC – chlormequat; AZS – azoxystrobin; CBD – carbendazim; CYA – cyproconazole; DEET – diethyltoluamide; DFB – diflubenzuron; EPO – epoxiconazole; IMD – imidacloprid; MAL – malathion; PBO – piperonyl butoxide; PRO – propiconazole; PYS – pyraclostrobin; TBA – tebuconazole; TFB – teflubenzuron; THO – thiametoxam; TCA – tricyclazole; TFM - triflumuron
